# BAIT: Organizing genomes and mapping rearrangements in single cells

**DOI:** 10.1186/gm486

**Published:** 2013-09-13

**Authors:** Mark Hills, Kieran O’Neill, Ester Falconer, Ryan Brinkman, Peter M Lansdorp

**Affiliations:** 1 Terry Fox Laboratory, BC Cancer Agency, Vancouver, BC V5Z 1L3, Canada; 2 Division of Hematology, Department of Medicine, University of British Columbia, 675 West 10th Avenue, Vancouver, BC V5Z 1L3, Canada; 3 European Research Institute for the Biology of Ageing, University of Groningen, University Medical Centre Groningen, A. Deusinglaan 1, NL-9713 AV Groningen, The Netherlands

## Abstract

Strand-seq is a single-cell sequencing technique to finely map sister chromatid exchanges (SCEs) and other rearrangements. To analyze these data, we introduce BAIT, software which assigns templates and identifies and localizes SCEs. We demonstrate BAIT can refine completed reference assemblies, identifying approximately 21 Mb of incorrectly oriented fragments and placing over half (2.6 Mb) of the orphan fragments in mm10/GRCm38. BAIT also stratifies scaffold-stage assemblies, potentially accelerating the assembling and finishing of reference genomes. BAIT is available at http://sourceforge.net/projects/bait/.

## Background

**Figure 1:**
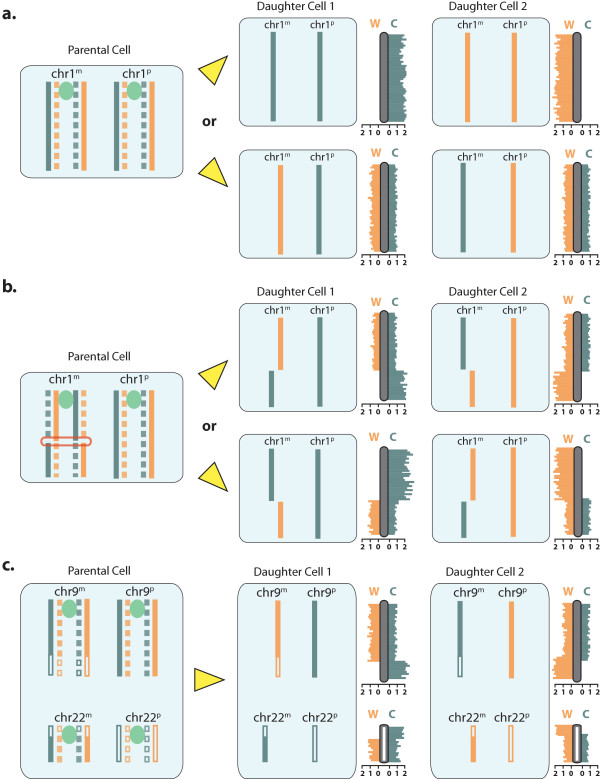
**Strand-seq involves sequencing of template strands only.** Newly formed DNA strands containing BrdU (dashed lines) in parental cells (left panels), are removed in daughter cells after cell division, hence only the original template-strand DNA is sequenced (solid lines, right panels). One template is derived from the Watson (W) strand (shown in orange), and the other template is derived from the Crick (C) strand (shown in blue); centromeres are shown in green. **(a)** Identification of template strands by Strand-seq. Daughter cells inherit two template strands because there is a maternal (m) and paternal (p) copy of each chromosome (chromosome 1 shown). Chromatids segregate either with both Watson strands inherited into one daughter and both Crick strands in the other (top panel), or with one Watson and one Crick strand in each daughter cell (bottom panel). Sequence read density is plotted onto ideograms (gray bars) representing the template state of each chromosome; the template-strand 'dose’ is inferred from W and C read counts (scale bar shown at bottom of ideograms). **(b)** Sister chromatid exchange (SCE) results in changes to templates on chromosomes. An SCE event (red outline) has reads aligning to different template strands on either side of it. These events are reciprocal between daughter cells, and will always be seen as a change from a WC state to either a CC or WW state. **(c)** Translocations and inversions are identified by Strand-seq. Translocations will align in the direction of the template strand of the chromosome to which they translocated, but still map to their original chromosome location. For example, for the Philadelphia translocation between chr9 and chr22, sequence reads from the translocated portion of chr22 will still map to chr22, but will have the template inheritance pattern of chr9 (chr9 fragments shown as solid boxes, chr22 fragments shown as open boxes).

We recently described a sequencing technique called Strand-seq for directional sequencing of DNA template strands in single cells [1,2]. To generate Strand-seq data, cells are cultured with the thymidine analogue 5-bromo-2′-deoxyuridine (BrdU) for one round of DNA replication. The newly formed DNA strands incorporate BrdU, and are selectively removed prior to library amplification, resulting in directional libraries consisting of only template strands. Sequencing of these libraries on an Illumina platform results in reads that map either to the 'Crick’ strand (plus or top strand) or the 'Watson’ strand (minus or bottom strand) of the reference genome. Because most eukaryotic genomes are diploid, the template strands from both chromosomal copies are represented, and the resultant directional reads can be output in the form of a chromosome ideogram (Figure 1a). Thus if a chromosome has reads mapping solely to the Watson strand, the cell has inherited a Watson template from each of the parental homologues (WW), whereas if it has reads mapping to both Watson and Crick, the cell has inherited one Crick-template and one Watson-template parental homologue (WC). This ability to discern which template strands were inherited by dividing cells can be used for a number of important applications including the high-resolution mapping of SCEs, analysis of genomic rearrangements, and refining of reference assemblies.

SCEs are the outcome of the repair of double strand breaks, and their accumulation is an early indicator of genomic instability [3]. Strand-seq data allows the identification and mapping of these events at unprecedented resolution [1]. The frequency of SCEs has been used as a surrogate for assessing the toxicity of mutagens [4], and as a diagnostic marker for disorders such as Bloom’s syndrome, which have a characteristically high frequency of SCEs [5]. Stand-seq can also detect translocations, inversions, deletions, and amplifications. Deletions and amplifications present as a loss or gain of reads over particular regions, and will locate to the same region across all libraries, making them easy to identify. Translocations and inversions appear identical to SCE events in individual libraries (Figure 1c), but can be resolved when the event locations are compiled across multiple libraries, as they will all occur over the same region. Preliminary data suggests that this approach works well in identifying and localizing chromosomal abnormalities (manuscript in preparation). It is further possible to apply Strand-seq to estimate the frequency of genomic rearrangements in a heterogeneous population of cells.

We showed previously that Strand-seq also has an application in correcting incorrectly oriented portions of the mouse reference assemblies [1]. Reference assemblies have become essential tools for aligning sequences and identifying variations, and thus, the need for a complete and accurate reference genome for any organism of interest is essential [6]. At present, a variety of organisms have been targeted for genome sequencing projects [7], and more established genomes are being continually updated. For example, the mouse reference genome was first published in 2002 [8], and has been periodically updated with more complete and corrected assembly versions. In most such iterations of reference assemblies, there are both gaps of unknown length within the sequence (typically regions difficult to sequence), and 'orphan scaffolds’ that have yet to be mapped to particular chromosomes or regions on specific chromosomes (likely to map within gaps, and lacking the tiling to form contiguous sequences). Although PCR-based approaches [9], forms of restriction mapping [10,11] and optical mapping [12] can be used to bridge these gaps or connect orphan scaffolds, there are still currently 628 gaps and 44 orphan scaffolds in the latest mouse reference assembly (GRCm38/mm10), and 357 gaps and 65 orphan scaffolds in the latest iteration of the human assembly (GRCh37/hg19). Many of the gaps are unbridged, representing spaces in the genome build of unknown length, and importantly, the relative orientation of sequences on either side of these gaps are also unknown. Furthermore, there are many early-build genome projects underway, most of which remain at the contig stage, consisting of thousands of contiguous sequences that are unplaced with respect to each other, and not localized to any chromosomes. With recent efforts aiming to rapidly generate reference genomes from 10,000 organisms [13,14], the need for alternate approaches to build the thousands of contigs from scaffold-level genomes into useable reference assemblies is paramount, and here we show that Strand-seq can perform a pivotal role in this.

Strand-seq has many applications for the study of tumor heterogeneity and evolution, and for genome instability in diseases of aging, as well as an enormous potential for rapidly building and refining the growing repertoire of reference assemblies. It is also an efficient technique, with the ability to sequence up to 200 indexed libraries simultaneously on a single lane. However, in order to analyze Strand-seq features across these large datasets, the technique needed an intuitive software package that could automate this process. Here we describe new open source software, Bioinformatic Analysis of Inherited Templates (BAIT), which builds upon our previously described plotting function [1] and enables high-throughput analysis of Strand-seq data. BAIT is a command line-driven application for UNIX platforms, available under the two-clause Berkeley Software Distribution (BSD) license [15].

## Implementation

### Data management and processing

BAIT provides a core framework for Strand-seq analysis, including functionality to plot W and C template strands, count aneuploid chromosomes, and map and enumerate SCE events (see Additional file 1: Figure S1). Extending these core functions for genome assembly, BAIT leverages strand-inheritance data to identify misoriented contigs, localize orphan scaffolds to specific chromosome regions on late-build genomes, and assemble early-build genomes *de novo* from non-overlapping fragments, using only one lane of sequencing containing up to 200 indexed libraries. In concert with Strand-seq, BAIT has major applications in detecting SCEs, analyzing sister chromatid segregation, and building and finishing genome assemblies.

BAIT accepts sequencing data in BAM format and parses it with SAMtools [16] to remove duplicate reads, threshold for quality, and discern read direction. These data are then fed to multiple R scripts (incorporating packages from Bioconductor [17]), which bin the data (200 kb windows by default), and compute strand inheritance, perform SCE analysis and plot chromosome ideograms showing read density, directionality, and predicted SCE events (Figure 1). Additional options in the command line allow alternate forms of output, additional plotting parameters, and the ability to convert data into BED files that are auto-formatted for UCSC genome browser upload using the BEDtools package [18].

The ability of BAIT to accurately assess SCE events and genome build analyses can be confounded by technical variability from the Strand-seq protocol, including spurious or constant low-background reads, or variable read depths. Much of this variability is presumably engendered by BrdU uptake by the cell, and the subsequent successful removal of the BrdU-incorporated (non-template) strand from the pre-amplified library. In order to aid decisions to remove low-quality libraries from further analysis, BAIT calculates this metric by first performing an unfiltered prediction of strand inheritance, then computing library background as the average frequency of spurious non-template-strand reads (C reads on chromosomes when homozygous W template strands were inherited, and *vice versa*). This value is expressed as a background percentage on each library ideogram.

A summary file is also generated (see Additional file 2: Supplemental Data File 1), including the frequency of WW, WC, and CC template inheritance for each intact chromosome for the analysis of sister chromatid segregation. The distributions of template strands are presented as pie charts, showing *P*-value significance from χ^2^ analysis after Holm correction [19]. BAIT also plots the template inheritance across each bin of every chromosome (see Additional file 2: Supplemental Data File 1), and creates BED files of the locations of all SCE events, which is useful for all subsequent analysis of Strand-seq data, such as mapping SCEs and genomic rearrangements.

The 62 Strand-seq libraries used in this study are publically available from the Sequence Read Archive SRA055924, and have been published previously [1]. BAIT took 81 minutes to process these libraries, with an average of 3,235,111 reads each, using a single core of an Intel i7-870 2.93 GHz processor on a computer with 16 Gb of RAM.

### Detection of sister chromatid exchanges, misorientations, and genomic rearrangements

**Figure 2:**
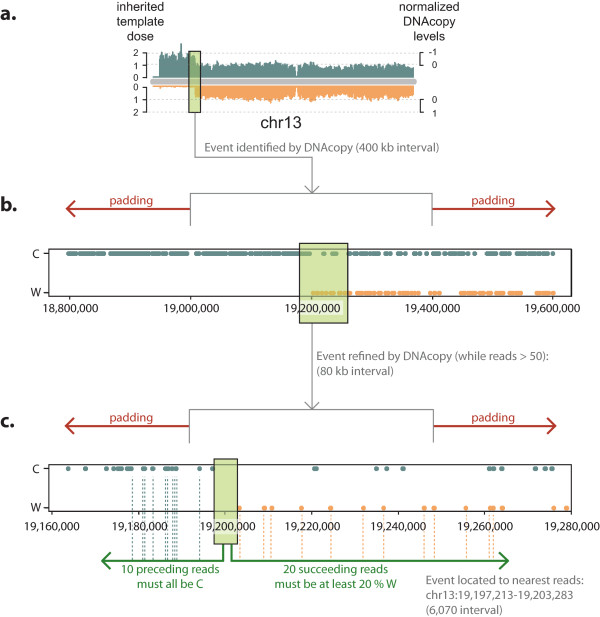
**Automated identification of sister chromatid exchange (SCE) from Strand-seq data. (a)** Gross directional mapping data are thresholded to remove bins with unexpectedly high or low read numbers, and analyzed using DNAcopy. Inherited template numbers are converted to a value between 1 and -1 for DNAcopy to make only one of three calls: WW, WC, or CC. DNAcopy defines an interval across two bins, so with a bin size set to 200 kb, the SCE event will be located to within 400 kb. **(b)** Localization is then iterated by subdividing the identified region into bins one-fifth of the original size (80 kb on first iteration), and re-running DNAcopy. A single bin size is used as padding to aid detection of SCE events at bin boundaries. The iterations of re-running DNAcopy continue until less than 50 reads remain within the interval. **(c)** A second algorithm identifies the first read to map in a different direction (W read at chr13:19,203,283), then performs a check that the 10 preceding reads are all in the expected direction (10 C reads), and at least 20% of succeeding reads are in the other direction. The interval is refined to a distance between two reads. Abbreviations: C, Crick; W, Watson.

SCEs are visualized on the chromosome ideograms as regions where reads switch from a homozygous template state (WW or CC) to a heterozygous template state (WC). Although the overall read depth is unchanged across an SCE, the proportion of directional reads will change from two copies in the homozygous state to one in the heterozygous state (Figure 1). BAIT exploits the similarity of the change in template copy number to copy number variation (CNV) analysis in order to locate and characterize all SCE events. It does this by calculating the ratio of Watson and Crick reads within each bin, using [(W-C)/(W + C)], and normalizing to the nearest integer. This gives a value of 1 when all reads map to the Watson strand (WW strand inheritance), -1 when all reads map to the Crick strand (CC), and 0 for an equal number of both (WC) (Figure 2a). A change in this ratio along the length of a chromosome corresponds to the location of an SCE event (Figure 2a), which is first localized to neighboring bins. For example, using the default bin size of 200 kb, a switch from a CC template-strand state in one bin (ratio = -1) to a WC template-strand state in a neighboring bin (ratio = 0) indicates that an SCE event occurred somewhere within the 400 kb interval encompassing those two bins (Figure 2a).

BAIT first makes gross event calls by utilizing the circular binary segmentation algorithm [20] implemented in the CNV Bioconductor package DNAcopy [21] to locate the SCE event to the two-bin interval. It then recalculates the template-strand ratio by segmenting this interval into five new bins (80 kb each using default bin size), narrowing the location of the SCE interval further. BAIT applies this binning-based DNA-copy detection method iteratively, decreasing the bin size by a factor of five each time (Figure 2b), until the read density is no longer sufficient to make accurate calls (determined to be when an interval has less than 50 reads, or when DNAcopy can no longer predict a single event (Figure 2c). In order to identify SCE events on the boundary of bins, BAIT pads each interval with one-half of the interval length in each direction (Figure 2b,c; red arrows).

BAIT then refines the gross interval by incorporating a simple walker algorithm that analyzes reads starting from the homozygous state, and reports the first read on the opposite template that represents a switch to a heterozygous state (Figure 2c; green box). From this refined interval, the walker checks that the 10 preceding reads map to the homozygous state, and that at least 4 of the 20 following reads map to the opposite template state (Figure 2c). If these criteria are not met, as may be the case where the background is high, BAIT continues to analyze the across the interval until they are met. These checks improved the localization of SCE events (see Additional file 3: Figure S2), and varying these thresholds did little to change the data. Through this two-step process, BAIT automatically detects and localizes SCEs with a high degree of confidence, plots them on ideograms, and creates a UCSC-formatted BED file of all SCE event intervals.

BAIT amalgamates all called SCE events across libraries to identify any locations that have multiple SCE events associated with them. It reports any SCE-like event that occurs over the same interval in more than one library, treating them as a potential structural (genomic rearrangement) event, and calculating the number of occurrences. Events occurring in the same location over multiple libraries either are regions of recurrent SCE, or represent translocations, deletions, or inversions (Figure 1c). In addition, duplications are identified using the CNV function across each chromosome, and chromosomal anueploidy is calculated by comparing the read depth of each chromosome to the average read depth within the (diploid) library. A chromosomal read depth of half the library average corresponds to a single copy (monosome), whereas 1.5× the library average corresponds to three copies (triploid).

Although SCEs show a transition from a homozygous to a heterozygous template state (WW to WC, or CC to WC) in Strand-seq libraries, transitions between two homozygous template states (WW to CC and CC to WW) are identified as misoriented fragments in the reference genome. Previously, we manually identified and localized these events to unbridged gaps, and confirmed a subset of misorientations by hybridization of directional probes [1]. BAIT distinguishes these events from SCEs, and writes the locations of these data to a separate CSV file. Invariably, misorientations in the reference genome will present as a template-strand switch in every Strand-seq library, so BAIT also computes the concordance across all libraries as a measure of robustness of the misorientation call. Because BAIT already calculates chromosomal aneuploidy, an SCE event in a monosome chromosome (W to C or C to W) will not be erroneously called as a misorientation (WW to CC or CC to WW).

### Stratification of early-build genome assemblies

Early-build genome assemblies consist of many contigs, which are effectively unanchored and unordered. However, performing Strand-seq on cells derived from organisms with early assemblies will yield directional strand information for each contig, and any contigs residing on the same chromosome will inherit the same templates. Contigs from different chromosomes will inherit template strands independently, and by chance, the templates will be the same in only half of all libraries. Conversely, adjacent contigs will inherit the same template strands across all libraries. By comparing all contigs together, it is possible to cluster them into putative chromosomes based on the concordance between them.

BAIT initially excludes libraries where every contig has inherited WC templates (probably a failed Strand-seq library), as well as individual contigs that have inherited WC templates in all libraries (probably a contig with degenerate sequences that cannot be placed). It then uses a two-stage approach to assemble the remaining contigs into a putative assembly. First, it clusters all contigs with highly similar template inheritance into linkage groups that represent individual chromosomes. It does this by comparing the two contigs represented across the most libraries, and assessing template-strand concordance between them; if they share a high concordance, they are classified together in a single linkage group, otherwise they are classified into separate linkage groups. Each remaining contig in the assembly is individually compared with the groups already assigned, and is then either added to a linkage group if it shares a high similarity with that group, or is classified into a new linkage group if it does not. This process continues until all contigs have been stratified into linkage groups or classified as single unlinked contigs. Ideally, the number of linkage groups is equal to double the number of chromosomes within the organism (a plus-strand and minus-strand linkage group for each chromosome).

**Figure 3:**
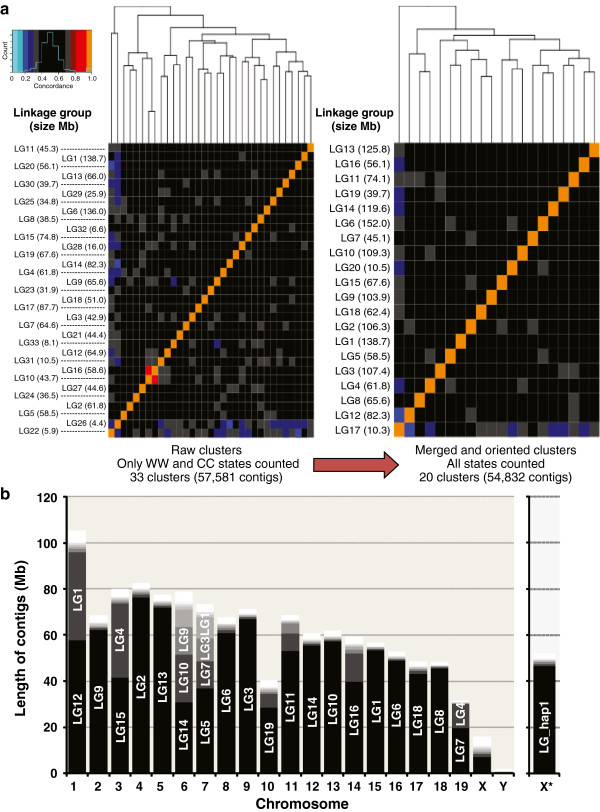
**Clustering contigs into linkage groups for early-assembly genomes.** Using template strand directionality as a unique signature, all contigs in the early mouse assembly MGSCv3 were compared with each other across all 62 Strand-seq libraries. All contigs with similar (>85%) template inheritance patterns were stratified into linkage groups (LGs). **(a)** Heat plots of all BAIT-called LGs show limited similarity between groups. Through analysis of homozygous template states only (WW and CC, left panel) 57,581 contigs cluster into 33 LGs, with the association between linkage groups appearing as yellow points if groups are in the same orientation, or blue points if the groups are in opposite orientations. The LGs are then reanalyzed after merging and reorientation of associated clusters, resulting in only 20 linkage groups consisting of 54,832 contigs. **(b)** Histogram of the number of fragments within a linkage group that map to a particular chromosome. The LG with the largest number of contigs are shown at the bottom in dark gray, with groups that contain the next largest numbers of contigs shown in progressively lighter grays. Most LGs contain contigs that belong to the same chromosome (see Additional file 4: Figure S3), and in general, most chromosomes are represented by one or two linkage groups. Note: contigs derived from sex chromosomes in male libraries can be distinguished as they are haploid, and are not computed as an initial heat plot. Any contigs derived from haploid chromosomes are separated and clustered independently. Almost all contigs clustered into this linkage group mapped to the X chromosome (right histogram). Abbreviations: C, Crick; W, Watson.

To distinguish contig orientation, BAIT generates an initial contig dissimilarity matrix using only chromosomes that have inherited homozygous WW and CC templates (but excluding WC), in such as way that misoriented linkage groups derived from the same chromosome are highly dissimilar (Figure 3a, left panel). BAIT then uses a simple greedy algorithm to reorient the misoriented linkage groups, iteratively inverting the most dissimilar, and recomputing the distance matrix until a reorientation causes no increase in the summed concordance of all groups (Figure 3a, right panel; see Additional file 4: Figure S3). Linkage groups with high similarity are merged in the recomputed data, and BAIT visualizes this as a distance-matrix heat plot of linkage group concordance (Figure 3a, right panel; see Additional file 4: Figure S3).

The second stage in BAIT scaffolding is performed individually on each linkage group/putative chromosome, by analyzing the contigs within each group. These contigs are compared with each other, and a relative order is computed based on template-strand concordance. If a chromosome had no SCEs in any libraries analyzed, every contig from that chromosome will share an identical template-strand inheritance, and their order cannot be determined. However, because SCEs switch template-strand inheritance along chromosomes, every SCE event will switch template strands along linkage groups (LGs), and therefore stratify the contigs within it. A single SCE event will split LGs into a cluster of contigs with homozygous WW or CC template inheritance to one side of the SCE event, and a cluster of contigs with heterozygous WC templates to the other side of the SCE event. In this way, the cumulative SCEs on any particular chromosome can be compiled across all libraries to help order contigs within the LG.

Similar to how meiotic recombination is used to create a genetic linkage map between loci [22], SCE events along the chromosome can be used to determine a genetic distance between contigs on the same chromosome, allowing them to be arranged and ordered. Adjacent contigs will have a lower probability of an SCE between them and a higher chance of inheriting the same template strands across all the libraries compared with contigs at opposite ends of the chromosome, which will be far more likely to have an SCE event between them. BAIT uses template-strand inheritance and SCE localization to build an inter-contig distance matrix for each linkage group. Then, using a traveling salesman algorithm (similar to finding the shortest route to take for traveling to multiple destinations only once) [23], BAIT calculates the shortest path through the distance matrix on each chromosome, thereby inferring the relative order of contigs within a linkage group.

### Stratification of late-build genome assemblies

**Figure 4:**
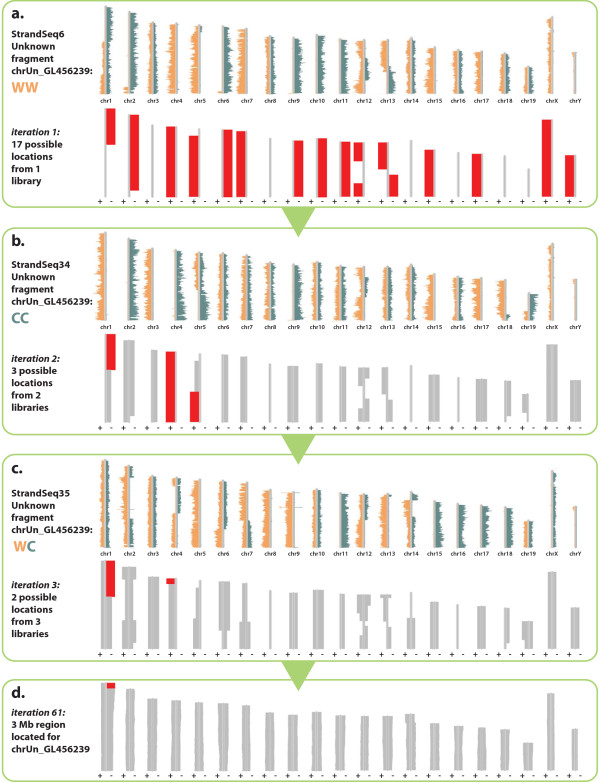
**Bioinformatic Analysis of Inherited Templates (BAIT) localizes unplaced scaffolds in late-version assemblies.** Orphan scaffolds can be correctly oriented and localized relative to the rest of the genome by comparing template-strand inheritance. The orientation of an orphan scaffold is arbitrary, because it is not anchored to the rest of the genome, so it can be correctly oriented with respect its located chromosome, or misoriented. **(a)** For a single library where the unplaced scaffold GL456239.1 is WW, BAIT maps its potential location (shown in red) to both WW genomic regions (correctly oriented), and CC genomic regions (misoriented). If only one library is analyzed, all locations map with 100% concordance. Note that a WW scaffold will not locate to a WC chromosome, so chr8, chr14, chr16, chr18, and chr19 are 0% concordant. **(b)** BAIT iterates over a second library where GL456239.1 is CC. The results of the two libraries combined reduce the number of potential mapping locations from 17 to only 3 that map with 100% concordance. Because chr8, chr14, and chr16 are WC in this library also, these chromosomes map with 0% concordance. **(c)** BAIT iterates over a third library where GL456239.1 is WC, and thus maps to all chromosomes that are WC. The result of the three combined libraries reduces the number of potential mapping locations to 2: the centromeric tips of chr1 and chr4. **(d)** The combined results after iteration of all 62 libraries refine the location of GL456239.1 to the first 10 Mb of chr1 in the reverse orientation (with a concordance of 91%). The fragment was further refined to an unbridged gap occupying the first 3 Mb of chr1. Abbreviations: C, Crick; chr, chromosome; W, Watson.

Using scaffold-level and chromosome-level assemblies to generate functional reference assemblies is valuable, but it is important to note that 'completed’ assemblies also contain a large number of contigs that remain unmapped. Assigning locations for these orphan scaffolds in a chromosome context is a high-priority endeavor for sequencing centers, and there are very few techniques that are available for this task [24]. However, provided that the orphan scaffold has sufficient read coverage, Strand-seq can be used to determine the strand-inheritance pattern, which will be the same as the chromosome on which it is present. For example, an orphan scaffold inheriting WC template strands must locate to a WC chromosome in that particular library. If an orphan scaffold inherits WW template strands, it will locate to a WW chromosome if both sequences are in the same orientation, or to a CC chromosome if it is misoriented with respect to the chromosome. On average, using just a single library, half of the chromosomes can be excluded as possible locations for these orphan scaffolds (Figure 4a).

By comparing these locations across a batch of libraries, BAIT localizes these scaffolds to particular chromosomes. For each orphan scaffold with sufficient reads, BAIT assigns a template state, compares this against the template state of each chromosome within a particular library, and then iterates this process to compute the concordance across all libraries. Concordance is never 100% in practice, owing to libraries with high background, orphan scaffolds with too few reads to accurately call strands, SCE events within gaps between the scaffolds, and the 5 to 10% error rate of BAIT in SCE detection. Nevertheless, BAIT is still able to achieve high-quality predictions of scaffold location by taking the highest-concordance chromosome. Chromosomes are further split based on SCE locations, allowing for localization of orphan scaffolds to particular chromosomal regions (Figure 4). Because orphan scaffolds are likely to be located within gap regions rather than within contiguous sequence, BAIT can use a provided BED-format gap file to cross-reference all mapped orphan scaffold locations to gaps within the same interval. BAIT outputs in a BED file both the best predicted region for each fragment and any candidate gaps within that region.

## Results and discussion

### Accurate localization and mapping of SCEs

To assess the ability to computationally identify SCE events, BAIT predictions were compared with 528 SCE events from 62 murine embryonic stem cell Strand-seq libraries that had previously been identified manually [1]. Manual processing of SCE events involved uploading BED-formatted Strand-seq data into the UCSC genome browser [25], and identifying the interval at which the templates switch. Initial comparisons showed that although BAIT identified over 97% of SCEs called manually, it also displayed a high false-discovery rate. To reduce this rate, a user-changeable threshold was incorporated, which excludes any bins that deviate from the average read depth, and thus have fewer or greater reads than expected.

**Figure 5:**
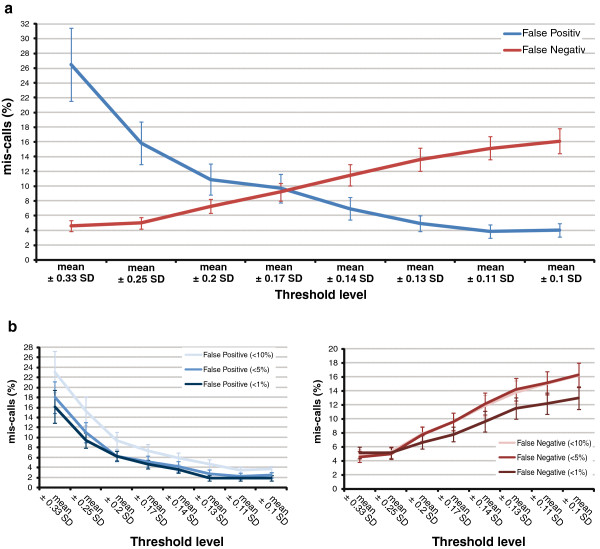
**Accuracy of automated sister chromatid exchange (SCE) detection by Bioinformatic Analysis of Inherited Templates (BAIT). (a)** By comparing the number of SCE events identified by BAIT to those determined manually, we calculated the percentage of computational calls that were incorrect (false positives) or not detected (false negatives). Filtering the data by only including bins that deviated minimally from the mean changed the results, with highly conservative filtering increasing the level of false negatives, and very broad filtering increasing the level of false positives. **(b)** The frequency of (left) false positives and (right) false negatives with respect to library background. Cleaner, high-quality libraries with < 1% of reads mapping incorrectly had a lower false-positive rate than libraries with medium background (<5% incorrectly mapped reads), and an even lower rate than libraries with high background (<10% incorrectly mapped reads). Error bars are ± standard deviation.

By comparing the BAIT SCE calling to the manually processed SCEs, we found the optimal threshold for these data was to exclude bins with read counts of ±0.2 standard deviations from the mean, which gave a sensitivity of 0.93 (10.9% false positives), and a specificity of 0.89 (7.2% false negatives) (Figure 5a). When only those libraries with a low background metric (<5%) were included, the specificity improved to 0.94, while the sensitivity remained almost the same at 0.92 (Figure 5b). Of the false-negative calls, 72.9% were SCEs within 5 Mb of the start or end of the chromosome, indicating that terminal regions of chromosomes are under-represented by BAIT’s SCE localization. In addition, three of the SCE events predicted by BAIT but absent in the manual analysis were determined to be correct upon further analysis. One event was less than 2 Mb from the distal telomere of chromosome 1, while the remaining two events were 5 Mb from each other on chromosome 13. These SCE events were difficult to detect by eye from a BAIT ideogram output of Strand-seq data. Furthermore, because BAIT identifies SCE locations directly on ideograms with an arrowhead, both false-positive and false-negative SCEs can be rapidly scanned and validated from the ideogram output files.

Of the correctly identified SCE events, a comparison of the location of the SCE interval between automated and manual calls showed a median difference of just 34 bp (see Additional file 3: Figure S2). Almost two-thirds (65.8%) of the predictions were within 100 bp of the manual calls, with 74.7% of predictions within 10 kb. A summary of SCE distribution across all libraries was plotted, together with a histogram reporting the distance between events, helping to identify significant clustering of SCEs (see Additional file 2: Supplemental Data File 1). The accurate identification of SCEs is also important for the functions of BAIT which assemble and refine reference genomes (see sections below).

BAIT facilitates SCE analyses by rapidly counting and locating events, presenting a pipeline that can be incorporated into high-throughput strategies. BAIT accurately refines the interval between reads in which the template switch occurs, allowing regions with a high propensity to undergo SCE to be identified (for example, fragile sites [26] or sites of recurrent DNA damage). Accurate interval identification is also important in looking for genomic rearrangements such as translocations, and BAIT is able to detect these and assign a frequency of the rearrangement within the pool of libraries, requiring a far lower read depth than conventional split-pair read sequencing [27]. A caveat to these analyses is that SCEs and genomic rearrangements are more difficult to detect on chromosomes that have more than two copies within a cell, potentially limiting its use in highly polyploid cancer cells. Taken together, our results show that BAIT is very accurate and efficient at predicting SCE intervals, and will be indispensable for future high-throughput analysis of Strand-seq data.

### Improving early-stage reference genome builds

To test the ability of BAIT to build genomes *de novo*, we realigned our libraries to the first build of the mouse genome (MGSCv3). Of the 224,713 contigs in this assembly version, we included in the analysis the 77,258 that were over 10 kb, representing 2,006 Mb of DNA (81.0% of total assembly). After remerging and reorienting similar clusters, BAIT assigned 54,832 contigs, representing 1,742 Mb (64.9%) of the assembly, into 20 primary LGs (Figure 3a). Allosomes in these male-derived ESCs are effectively monosome, and so contigs derived from the sex chromosomes can be separately identified, as they only inherit a single W or C template strand, never both. After cross-referencing the locations of MGSCv3 contigs to GRCm38/mm10 coordinates, the majority of LGs clustered to only one chromosome (see Additional file 4: Figure S3), and the majority of chromosomes consisted of only one linkage group (Figure 3b). When more than one chromosome was attributed to the same linkage group, these groups could be split into two subclusters (see Additional file 4: Figure S3).

Similar results were seen when we simulated an early-stage reference by splitting the GRCm38/mm10 genome into a scaffold of the 403 chromosomal Giemsa bands (based on coordinates from the UCSC genome browser [28]), and realigned our libraries to this new reference version (see Additional file 5: Figure S4). Using disrupted concordance from SCEs as a genetic distance indicator, it was further possible to infer the relative orders of the contigs present in each linkage group.

The accuracy of ordering fragments is dependent on the frequency of SCEs, the number of libraries used in the analysis, and the level of library background (high-background libraries are more likely to have incorrect template calls). If the template strands of contigs are identical in all libraries (because no SCE events have occurred between them) their relative order remains unknown.

Taken together, these data show that with only a single lane of sequencing and just 62 Strand-seq libraries, BAIT can aid in the rough draft assembly of a scaffold-level reference genome. Importantly, preliminary sequencing efforts in lesser-studied organisms suffer from fewer resources spent on deep sequencing and subsequent curating and refining of the reference genome assemblies. With several ambitious sequencing projects in development [13], there is an increasing need for rapid and cost-effective construction of accurate and useful reference genomes. Arranging contigs to facilitate building chromosome-level and genome-level hierarchy represents an attractive advance toward this goal, especially in conjunction with existing technologies. We have shown that BAIT can effectively 'stitch’ contigs together based on shared template inheritance, and rapidly construct a useful skeleton assembly that can be built upon, and believe this technique will be widely adopted in standard genome assembly pipelines.

### Refining and finishing completed reference assemblies

We have previously shown using Strand-seq that over 20 Mb of the MGSCv37/mm9 *Mus musculus* reference assembly is misoriented, involving 17 regions flanked by unbridged gaps [1]. In the more recent GRCm38/mm10 build of the genome, 35% (7,079.49 kb) of these identified misorientations were subsequently corrected, validating Strand-seq with other approaches to correct orientation issues. In order to identify misorientations in the newest GRCm38/mm10 assembly, we repeated these analyses using the automated function of BAIT, identifying a total of 15 misoriented regions and 5 autosomal misorientations, with the remaining 10 located to the X chromosome (see Additional file 6: Table S1). Because the X chromosome only exists as one copy (monosomy) in the male embryonic stem cells (ESCs) of our dataset, misorientations appear indistinguishable from SCEs, and were identified by the intersection of events occurring over the same region across all libraries (see Additional file 2: Supplemental Data File 1). In this way, using just a single lane of sequencing, we were able to orient the majority of contigs (those larger than 10 kb with minimal segmental duplications) with respect to flanking contigs. Thus, using Strand-seq and BAIT with relatively low-coverage sequencing, the relative orientation of all reference contigs can be determined, effectively bridging all gaps in an assembly.

**Figure 6:**
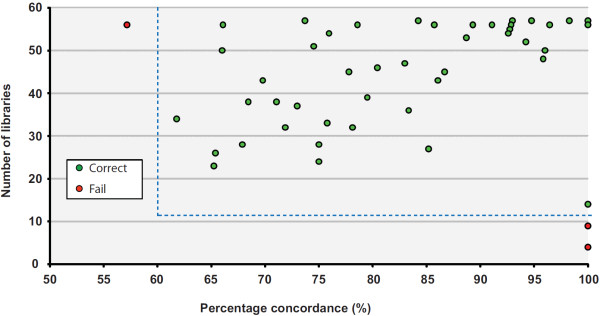
**Validation of using Strand-seq to map unplaced scaffolds to built genomes.** To confirm that Bioinformatic Analysis of Inherited Templates (BAIT) can successfully locate orphan scaffolds, the reads were aligned to MGSCv37/mm9, which has 202 orphan scaffolds, of which 60 can be mapped to a specific location in GRCm38/mm10. We used BAIT to locate these scaffolds in MGSCv37/mm9, and then cross-referenced these locations to the actual location in the GRCm38/mm10 assembly version. BAIT correctly located all regions in which there were more than 10 libraries to analyze, and where the percentage concordance was above 68%. Green points indicate correctly mapped fragments, and red points indicate incorrectly mapped fragments. Dashed lines show the minimum number of libraries and minimal concordance needed to make confident calls.

To validate the ability of BAIT to map scaffolds that have yet to be localized to regions on reference assemblies, we used it to predict the localization of all orphan scaffolds in an earlier assembly of the mouse reference (MGSCv37/mm9), and compared those predictions with the actual known locations in the current assembly (GRCm38/mm10). MGSCv37/mm9 has 60 useable orphan scaffolds that can be lifted to a single specific coordinate on GRCm38/mm10 [28]. Of these, 57 were located by BAIT to an interval coincident with the correct location on GRCm38/mm10 (Figure 6). From the three fragments that could not be correctly placed, two had fewer than 10 libraries with sufficient read counts to analyze, and the remaining fragment mapped with a low concordance (57.1%). These data suggest reasonable thresholds for BAIT to map orphan scaffolds: more than 10 libraries and greater than 60% concordance. More importantly, they confirm that using data from the same single lane of sequencing as used for contig orientation, BAIT and Strand-seq can correctly map a large proportion of orphan scaffolds in a late assembly version.

There remain 44 orphan scaffolds in GRCm38/mm10, accounting for 5,334,105 bp, and containing 41 known genes. Of these, 23 contained sufficient reads to analyze, and we were able to subsequently place all of them to their matching chromosomes to within narrow intervals (Table 1; see Additional file 7: Supplemental Data File 2). By intersecting these locations to gaps in the contiguous genome build, BAIT further refined the scaffold locations (Table 1). Fragments were assumed to locate within either unbridged gaps or to bridged gaps in which gap size exceeded the fragment size, Analyzing 62 mouse libraries, 54.5% of these orphan scaffolds could be mapped to a particular chromosome, of which 54.2% could be mapped to a single contig gap (Table 1). BAIT also correctly oriented these fragments with respect to the chromosome to which they were mapped. For established and well-studied genomes, finishing builds by additional sequencing yields diminishing returns, and novel, targeted and highly sequence-efficient methodologies such as Strand-seq and BAIT can play a crucial role in completing these genomes. BAIT includes a utility to create a new FASTA reference genome by reverse complementing misoriented regions and incorporating orphan scaffolds that map to a defined gap.

**Table 1:** **Locations of unplaced scaffolds on GRCm38/mm10**^**a**^

**Accession number**^**b**^	**Scaffold size, kb**	**Localizes to: ,Mb**^**b**^	**Strand direction**^**b**^	**% Conc**^**c**^	**Libraries**^**c**^**, n**	**Gaps**^**d**^		**Gap location**^**e**^	
						**(u), n**	**(b), n**	**Primary**	**Alternate**
GL456382.1	23.2	chrX:0–57.6	+	100	18	14	26		
GL456379.1	72.4	chrX:0–57.6	-	97.8	46	14	26		
GL456233.1	336.9	chrX:0–57.6	-	96.1	51	14	26		
JH584299.1	953.0	chr5:90.4-96.6	+	94.2	52	0	1	chr5:94,088,336-94,138,335	
GL456239.1	40.1	chr1:0–12.6	-	91.1	56	3	1	chr1:0–3,000,000	
GL456367.1	42.1	chrX:0–57.6	-	90.0	40	14	26		
GL456381.1	25.9	chrX:0–57.6	-	90.0	50	14	26		
GL456393.1	55.7	chr3:28.6-31.6	+	89.3	56	0	0	chr3:40,550,618-40,650,617	
GL456359.1	23.0	chr4:136.4-156.2	-	88.7	53	1	19	chr4:156,408,117-156,508,116	chr4:130,393,226-130,516,309
GL456354.1	196.0	chr5:90.4-100.6	-	88.6	35	0	1	chr5:94,088,336-94,138,335	
GL456385.1	35.2	chr13:0–6.8	-	87.3	55	3	0	chr13:1–3,000,000	
GL456360.1	31.7	chr15:88.4-103.8	+	87.0	54	1	3	chr15:103,943,686-104,043,685	
GL456366.1	47.1	chr15:62.4-103.8	+	85.5	55	1	3	chr15:103,943,686-104,043,685	
GL456216.1	66.7	chr4:136.4-156.2	+	80.4	51	2	19	chr4:156,408,117-156,508,116	chr4:130,393,226-130,516,309
JH584296.1	199.4	chr5:83.6-113.2	-	80.0	10	1	1	chr5:113,521,975-113,535,974	
JH584297.1	205.8	chr5:88.6-100.6	-	77.8	18	0	1	chr5:94,088,336-94,138,335	
GL456368.1	20.2	chr4:129.2-156.2	-	76.2	42	2	19	chr4:130,393,226-130,516,309	chr4:156,408,117-156,508,116
GL456221.1	207.0	chr1:79.8-123.2	+	73.7	57	2	4	chr1:85,347,104-85,447,103	chr1:75,055,557-75,121,556
GL456392.1	23.6	chr2:0–8.2	-	73.5	34	4	0	chr2:0–3,050,000	
JH584292.1	14.9	chr4:107.8-108.6	+	73.5	49	0	1	chr4:99,842,111-99,876,234	
GL456372.1	28.7	chr1:127.2-146.2	-	69.2	52	1	1	chr1:156,118,744-156,168,743	
GL456389.1	28.8	chrX:0–57.6	-	63.6	33	14	26		
GL456370.1	26.8	chr4:67.6-68.8	-	62.0	50	0	3	chr4:61,344,177-61,394,176	

^a^Of the 44 orphan scaffolds, 23 had enough reads to determine their genomic location by calculating mapping concordance.

^b^The scaffold accession numbers and BAIT-determined locations are given, together with the strand direction, which gives the relative orientation of the scaffolds with respect to the genome.

^c^The percentage concordance (% conc) and the number of libraries with enough information to make a concordance call are also given.

^d^Finally, BAIT cross-referenced these locations to unbridged and bridged gaps falling over the interval (gap (u) and gap (b) respectively.

^e^Primary and alternate gap locations are given. To ensure no regions were missed, gaps were included within 10 Mb away from the determined interval.

## Conclusions

BAIT provides the functionality to realize several powerful and exciting applications of Strand-seq: strand inheritance, SCE analysis, genomic rearrangements, and finishing genomes. With a robust strand-inheritance analysis tool and accurate SCE calling, BAIT is able to interrogate Strand-seq data to follow template-strand segregation patterns, and is currently the most informative technique for testing such patterns [29-32]. In being able to identify SCE events to a kilobase resolution in one cell division (compared with a megabase resolution and two cell divisions for standard cytogenetic analysis [33,34]), Strand-seq offers a unique tool to examine regions of recurrent damage, and enumerates events in cells that have differing genetic backgrounds or have been subjected to different damaging agents. Crucially, these events can be independently assayed and mapped in individual chromosomes at a very high resolution without relying on cytogenetic expertise. In addition, we present here a novel use of template-strand analysis to localize fragments and orient contigs, which has yielded a more refined mouse reference assembly with 20.8 Mb of contigs corrected (see Additional file 6: Table S1) and 2.7 Mb of orphan scaffolds localized to specific regions (Table 1). The ability to refine assemblies can be expanded to systematically stratify the thousands of scaffolds that make up early-version reference genome endeavors without the need for overlapping contigs to determine orientation or relative order. Taken together, BAIT will be indispensable for future Strand-seq studies, and we foresee its widespread adoption in a number of applications, most notably for refining and finishing assemblies at various levels of completeness.

## Availability and requirements

• **Project name:** BAIT.

• **Project homepage:** See reference [15].

• **Operating system:** Linux.

• **Programming language:** BASH and R.

• **Other requirements:** SAMtools version 1.17 or higher, BEDtools version 2.17.0 or higher, R version 3.0 or higher, DNAcopy R package, gplots R package.

• **License:** Two-clause BSD.

• **Restrictions for non-academics:** license needed.

## Abbreviations

BAIT: Bioinformatic Analysis of Inherited Templates; BAM: Binary alignment map; BED: Browser Extensible Data; BrdU: 5-bromo-2′-deoxyuridine; BSD: Berkeley Software Distribution; CNV: Copy number variation; CSV: Comma-separated values; ESC: Embryonic stem cell; GRC: Genome Reference Consortium; LG: Linkage group; NCBI: National Center for Biotechnology Information; SCE: Sister chromatid exchange; UCSC: University of California Santa Cruz.

## Competing interests

The authors declare that they have no competing interests.

## Authors’ contributions

MH wrote the paper, designed the figures, performed the analysis. and wrote the software. KO incorporated the SCE-identification function and the unknown fragment plotter into BAIT, and wrote the software. EF developed Strand-seq and helped to write the paper. RB helped with selection of computational approaches. PML conceived of the study and helped to write the paper. All authors read and approved the final manuscript.

## Supplementary Material

Additional file 1: Figure S1 Flow chart of Bioinformatic Analysis of Inherited Templates (BAIT) pipeline. BAIT consists of a central Bash script involved in executing command line options and processing directional information from sequence data to be read into downstream R-scripts. Central decision options include generating contig orders from early build genomes, executing sister chromatid exchange (SCE) analysis and scanning the data for orphan scaffold alignment. Command-line options are shown as green labels adjacent to the arrows, with the input/output files represented as black rounded rectangles, and the graphical output files represented by grey rounded rectangles. Click here for file

Additional file 2 **Bioinformatic Analysis of Inherited Templates (BAIT) summary files, highlighting the locations of sister chromatid exchanges (SCEs) and misorientation events, as well as the minimal distances between SCE events across libraries.** For interpreting template strand inheritance, pie charts and bin-level histograms are generated across all libraries being analyzed. Click here for file

Additional file 3: Figure S2 Optimization of the sister chromatid exchange (SCE) interval detector function. After DNAcopy has identified the smallest region in which SCE events have occurred, Bioinformatic Analysis of Inherited Templates (BAIT) executes a function that scans the region from the homozygous template direction until it identifies the first read mapping to the opposite strand. These 'unfiltered calls’ were similar to the manual calls, but were subject to low level background reads interfering with accurate localization (red line). To circumvent this, we added to the function that checks the 10 preceding reads to ensure they are all the same state, which yielded more accurate calls (blue line). Finally, we added a further check to ensure that the succeeding 20 reads (which are supposed to be Watson and Crick (WC)) mapped to the opposite strand at least 20% of the time (green line). Click here for file

Additional file 4: Figure S3 Contig locations in Bioinformatic Analysis of Inherited Templates (BAIT)-compiled linkage groups. Strand-seq data was aligned to the MGSCv3 assembly, and was stratified solely on strand inheritance patterns. The resulting data were compared directly to the known locations of the MGSCv3 contigs in the current GRCm38/mm10 assembly. **(a)** Linkage groups determined by BAIT predominantly contain contigs derived from a single chromosome. The linkage groups (denoted as LG# under each histogram) contain different numbers of clustered contigs, with the total length of each linkage group shown (*y*-axis) but tend to map to only one chromosome. **(b)** Of the linkage groups that map to more than one chromosome, a heatmap plot shows that the linkage group should be subdivided. Linkage group 1 (green highlight) contains contigs from chromosome 1 (chr1), chr15, and chr7, but generates three distinct clusters (left panel) where each cluster contains contigs derived from one chromosome (colours beneath dendrogram). An example of a linkage group with contigs mapping to a single locus (LG11, green highlight) shows that the majority of contigs within this group cluster tightly together (right panel). Click here for file

Additional file 5: Figure S4 Validation of Strand-seq to cluster fragments to unanchored genomes. By measuring template concordance between mouse GRCm38/mm10 fragmented by chromosome banding pattern, a heat map was generated comparing the 403 scaffolds, of which 400 had enough reads across all libraries to accurately assign a template state. Scaffolds that inherited identical templates across all 62 libraries had a 'concordant distance’ of 1.0 and are displayed as orange blocks. Because sister chromatid exchange (SCE) events change the template state in some libraries, the 'concordant distance’ between contigs decreases, indicating that they are further apart. Scaffold orientation was determined through a greedy algorithm, and concordances were adjusted prior to plotting. To confirm that fragments were clustering correctly, their locations on the heat map were compared with the known chromosome (numbers below plot), showing a near-perfect clustering of scaffolds derived from the same chromosome. Three fragments had template states that appeared to occur independently of all the others (green boxes). Click here for file

Additional file 6: Table S1 Identification of all misoriented fragments in GRCm38/mm10. The genomic regions that were incorrectly oriented in the latest assembly version of the mouse genome were calculated by Bioinformatic Analysis of Inherited Templates (BAIT). The location and lengths of these regions, which should all be present in the reverse complement in the reference assembly, are shown. Misorientations were identified in every informative library. Click here for file

Additional file 7 **Supplemental Data File 2 is the Bioinformatic Analysis of Inherited Templates (BAIT) output of correlative data for all GRCm38/mm10 unlocalized scaffolds.** Standard output attempts to localize every genomic orphan scaffold by calculating the concordance of template strand inheritance across all libraries within each dataset (for an example, see Figure 4 for mapping of chrUn_GL456239). Each ideogram plot shows the likeliest location of the scaffold (red region), the percentage concordance (agreement between libraries), and the number of libraries used in the analysis. Click here for file
