# Supplementary material for: BAIT: Organizing genomes and mapping rearrangements in single cells
Source: Genome Med. 2013 Sep 13;5(9):82. doi: 10.1186/gm486 (PMC3971352; doi:10.1186/gm486)
Supplement: Additional file 1: Figure S1 — Flow chart of Bioinformatic Analysis of Inherited Templates (BAIT) pipeline. BAIT consists of a central Bash script involved in executing command line options and processing directional information from sequence data to be read into downstream R-scripts. Central decision options include generating contig orders from early build genomes, executing sister chromatid exchange (SCE) analysis and scanning the data for orphan scaffold alignment. Command-line options are shown as green labels adjacent to the arrows, with the input/output files represented as black rounded rectangles, and the graphical output files represented by grey rounded rectangles. [file gm486-S1.ppt]

## Slide 1
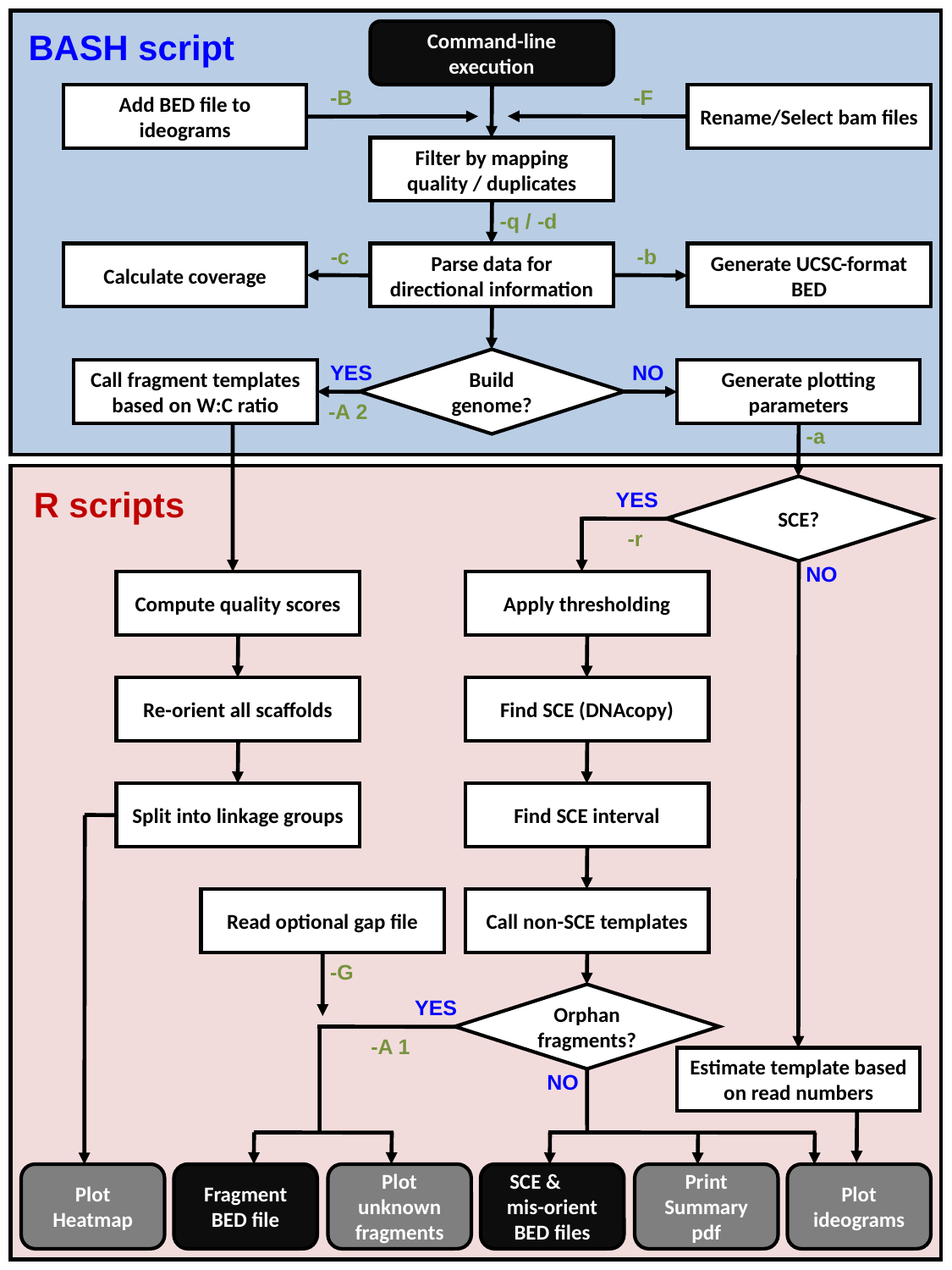

BASH script
Command-line execution
-B
-F
Add BED file to ideograms
Rename/Select bam files
Filter by mapping quality / duplicates
-q / -d
-c
-b
Calculate coverage
Parse data for directional information
Generate UCSC-format BED
Build genome?
YES
NO
Call fragment templates based on W:C ratio
Generate plotting parameters
-A 2
-a
R scripts
SCE?
YES
-r
NO
Compute quality scores
Apply thresholding
Re-orient all scaffolds
Find SCE (DNAcopy)
Split into linkage groups
Find SCE interval
Read optional gap file
Call non-SCE templates
-G
Orphan fragments?
YES
-A 1
Estimate template based on read numbers
NO
Plot Heatmap
Fragment BED file
Plot unknown fragments
SCE & mis-orient BED files
Print Summary pdf
Plot ideograms
