# Supplementary material for: BAIT: Organizing genomes and mapping rearrangements in single cells
Source: Genome Med. 2013 Sep 13;5(9):82. doi: 10.1186/gm486 (PMC3971352; doi:10.1186/gm486)
Supplement: Additional file 2 — Bioinformatic Analysis of Inherited Templates (BAIT) summary files, highlighting the locations of sister chromatid exchanges (SCEs) and misorientation events, as well as the minimal distances between SCE events across libraries. For interpreting template strand inheritance, pie charts and bin-level histograms are generated across all libraries being analyzed. [file gm486-S2.pdf]

SCE EVENTS PLOT FOR FINAL (200000 window)

SCE events= 505

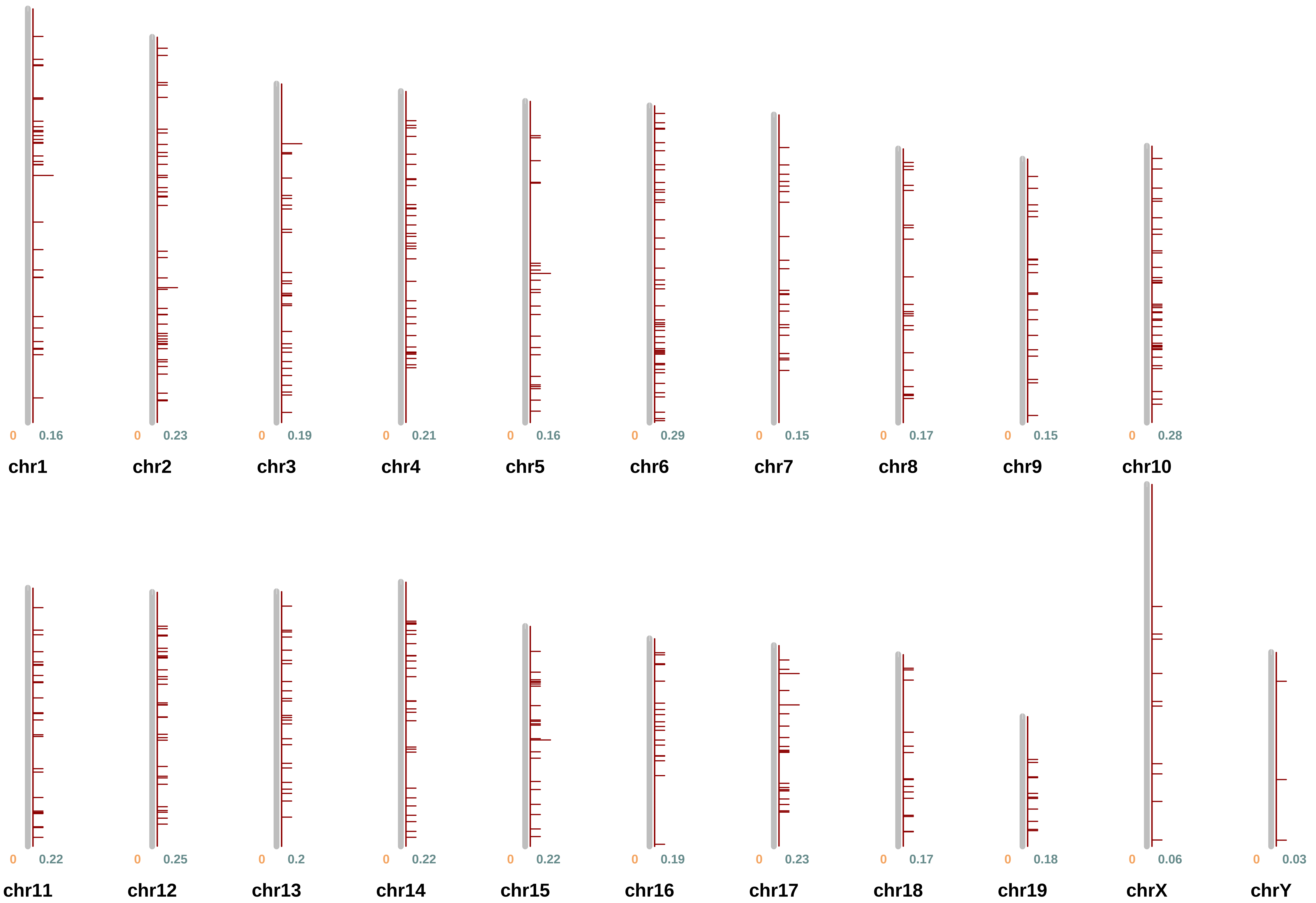

Quality filter=20 Organism=Mus_musculus

Aneuploid chromosomes 0

Normalized counts / Mb = 0.2 Scale = 0.12

SCE EVENTS PLOT FOR FINAL (1e+06 window)

SCE events= 505

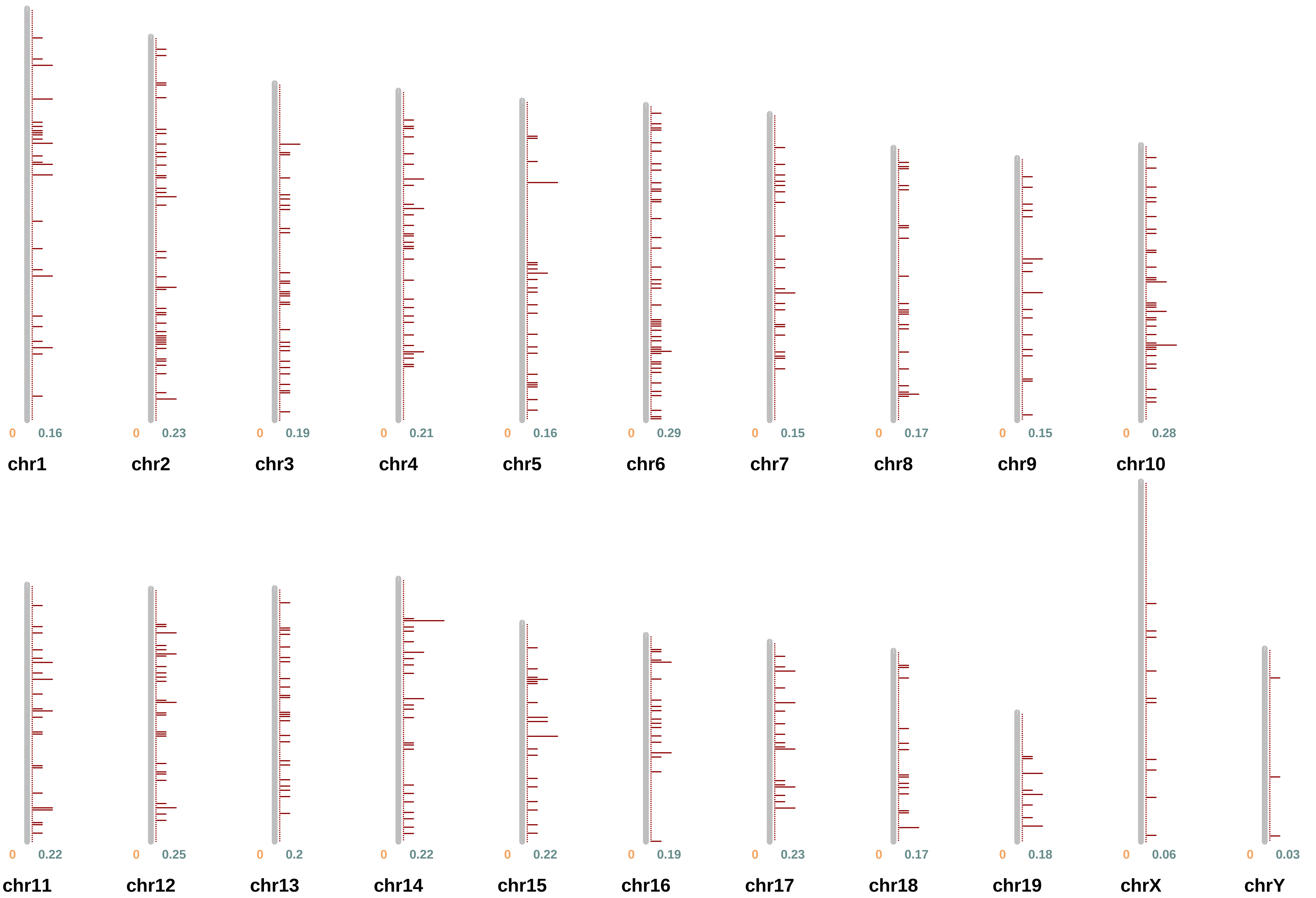

Quality filter=20 Organism=Mus_musculus

Aneuploid chromosomes 0 Normalized counts / Mb = 0.2 Scale = 0.12

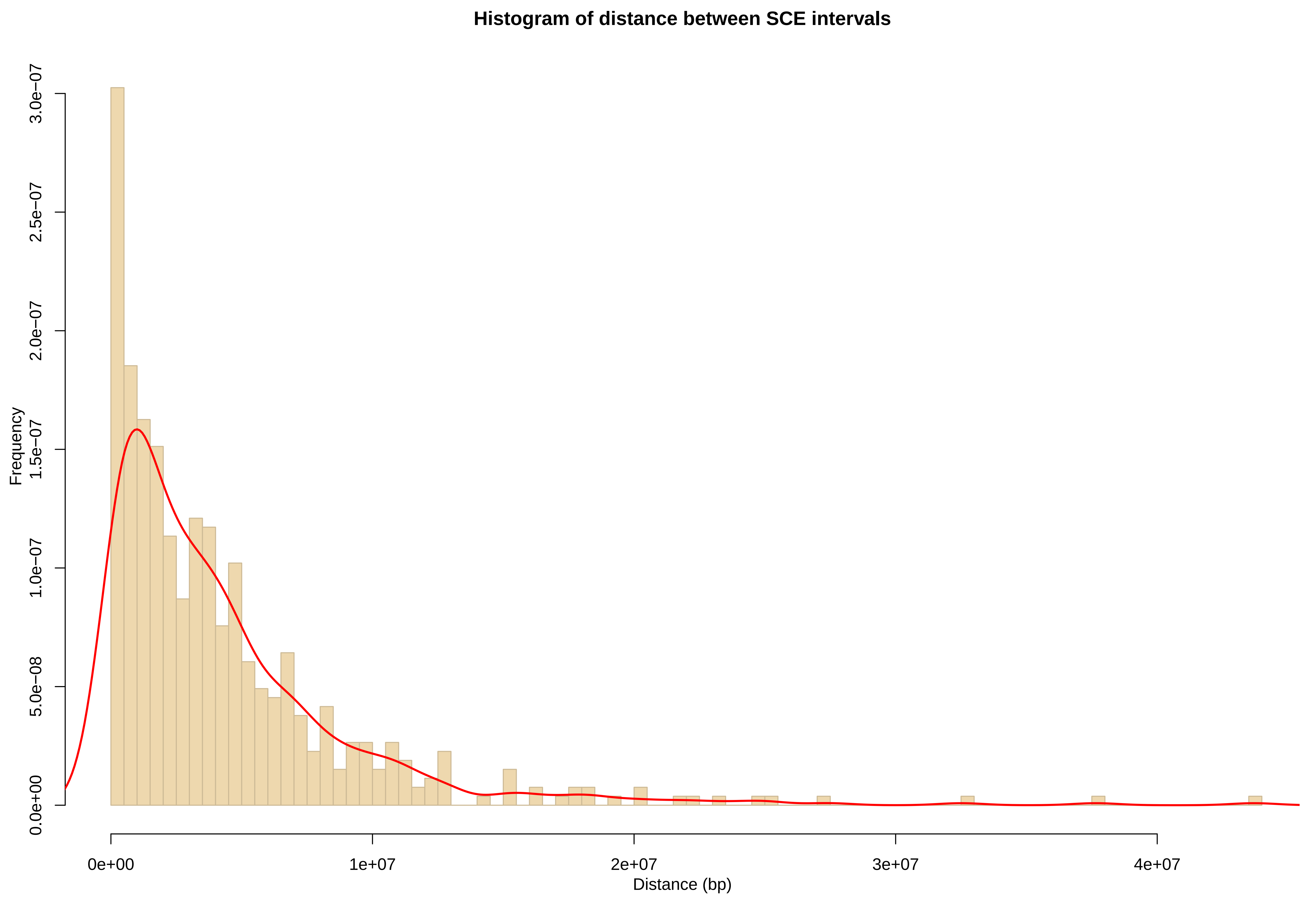

Histogram of distance between SCE intervals
Frequency
0.0e+00
5.0e−08
1.0e−07
1.5e−07
2.0e−07
2.5e−07
3.0e−07
0e+00
1e+07
2e+07
3e+07
4e+07
Distance (bp)

SWITCH EVENTS PLOT FOR FINAL Organism=Mus_musculus

Switch events= 45

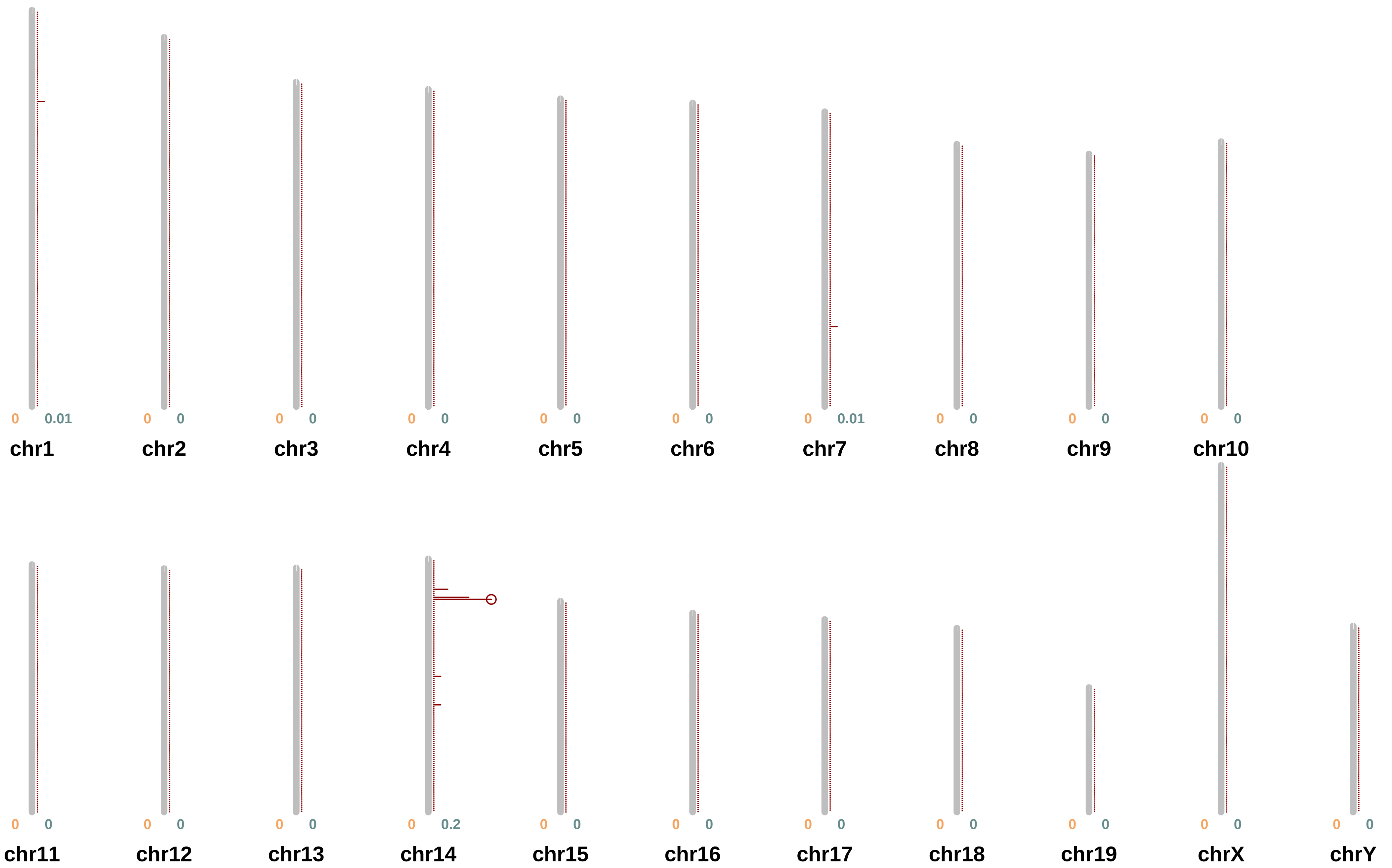

Quality filter=20

Aneuploid chromosomes 0

Normalized counts / Mb = 0.01 Scale = 0.19

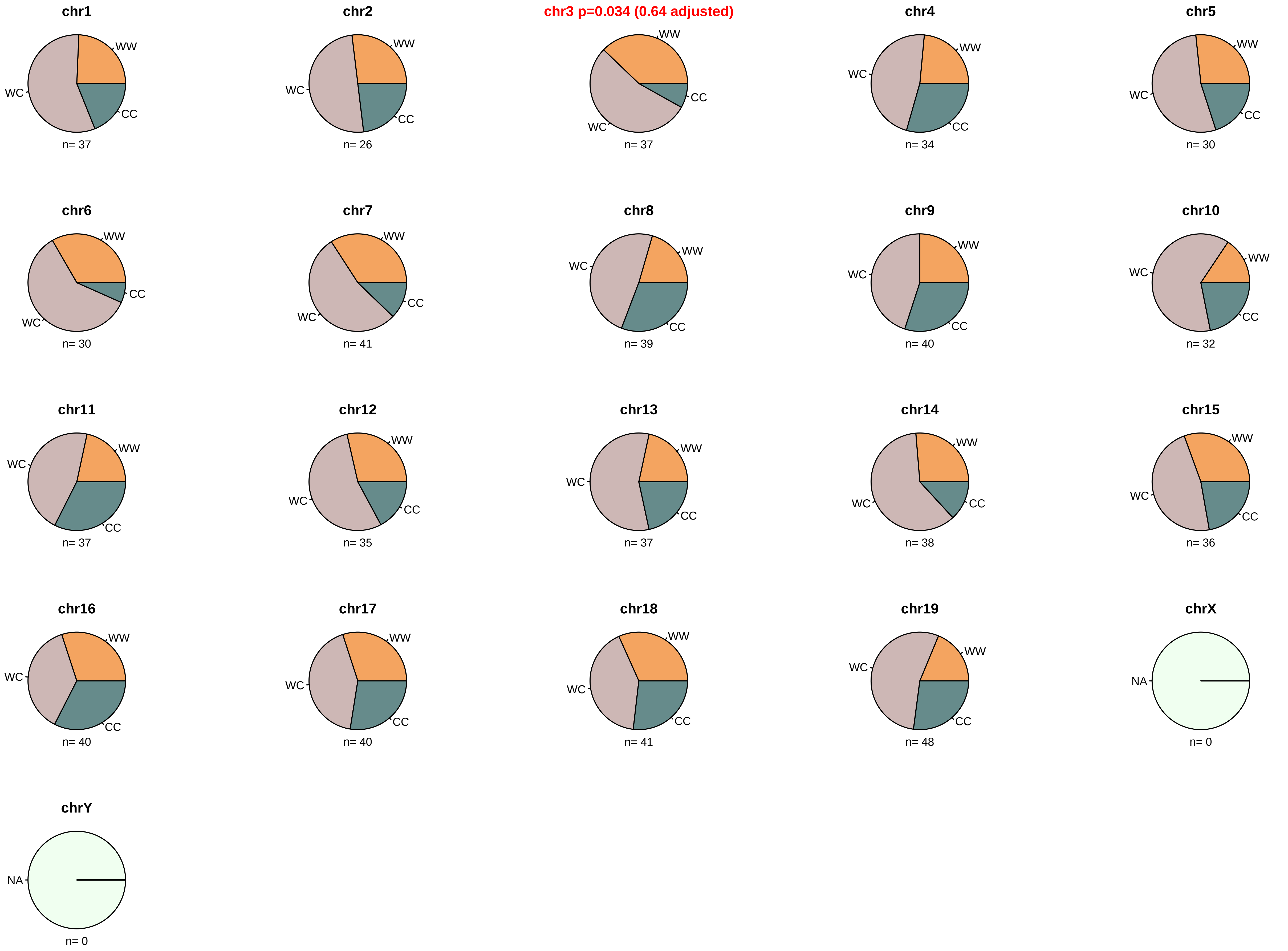
chr1
WW
WC
CC
n= 37
chr2
WW
WC
CC
n= 26
chr3 p=0.034 (0.64 adjusted)
WW
CC
WC
n= 37
chr4
WW
WC
CC
n= 34
chr5
WW
WC
CC
n= 30
chr6
WW
CC
WC
n= 30
chr7
WW
CC
WC
n= 41
chr8
WW
WC
CC
n= 39
chr9
WW
WC
CC
n= 40
chr10
WW
WC
CC
n= 32
chr11
WW
WC
CC
n= 37
chr12
WW
CC
WC
n= 35
chr13
WW
WC
CC
n= 37
chr14
WW
CC
WC
n= 38
chr15
WW
WC
CC
n= 36
chr16
WW
WC
CC
n= 40
chr17
WW
WC
CC
n= 40
chr18
WW
WC
CC
n= 41
chr19
WW
WC
CC
n= 48
chrX
NA
n= 0
chrY
NA
n= 0

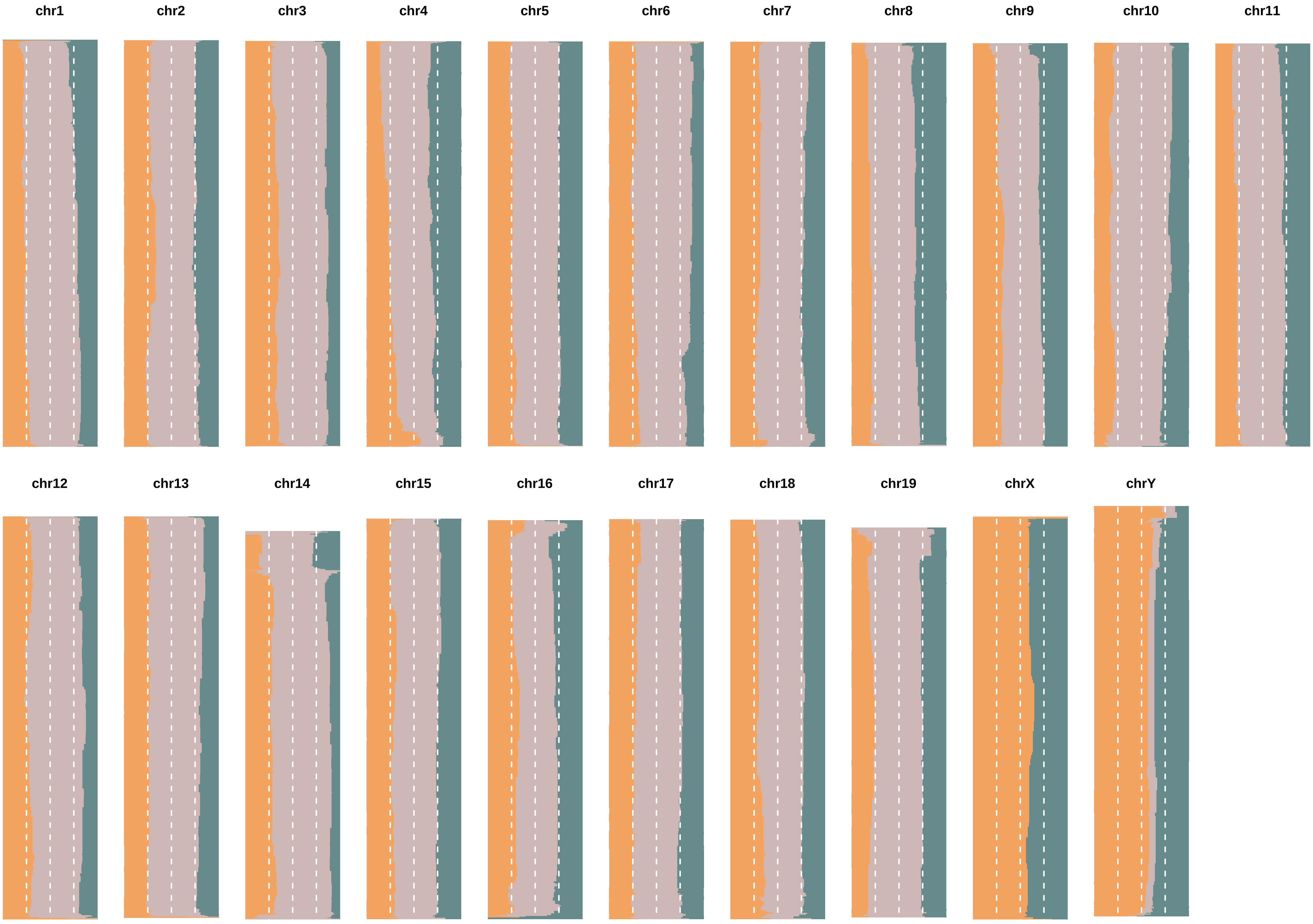
chr1
chr2
chr3
chr4
chr5
chr6
chr7
chr8
chr9
chr10
chr11
chr12
chr13
chr14
chr15
chr16
chr17
chr18
chr19
chrX
chrY
