# Supplementary material for: BAIT: Organizing genomes and mapping rearrangements in single cells
Source: Genome Med. 2013 Sep 13;5(9):82. doi: 10.1186/gm486 (PMC3971352; doi:10.1186/gm486)
Supplement: Additional file 6: Table S1 — Identification of all misoriented fragments in GRCm38/mm10. The genomic regions that were incorrectly oriented in the latest assembly version of the mouse genome were calculated by Bioinformatic Analysis of Inherited Templates (BAIT). The location and lengths of these regions, which should all be present in the reverse complement in the reference assembly, are shown. Misorientations were identified in every informative library. [file gm486-S6.doc]

**Supplemental Table 1: Identification of all mis-oriented fragments in GRCm38/mm10**

| **Fragment** | **Location** | **Size** |
| --- | --- | --- |
| JH584273.1 | chr4:130,516,309-130,579,475 | 63,167 |
| JH584273.1 | chr4:146,708,412-146,752,334 | 43,922 |
| GL456141.2 | chr8:20,257,550-20,443,136 | 185,586 |
| GL456225.2 | chr9:124,248,836-124,476,930 | 228,094 |
| GL456168.2 | chr14:3,000,000-19,419,705 | 16,419,705 |
| GL456186.2 | chrX:3,182,394-3,556,356 | 373,962 |
| GL456186.2 | chrX:4,742,210-4,960,182 | 217,972 |
| GL456190.1 | chrX:27,205,755-27,499,671 | 293,916 |
| GL456192.2 | chrX:27,549,670-29,212,945 | 1,663,275 |
| GL456194.2 | chrX:30,544,569-31,013,339 | 468,770 |
| GL456195.2 | chrX:31,919,956-32,327,544 | 407,588 |
| GL456195.2 | chrX:34,129,361-34,339,520 | 210,159 |
| GL456202.2 | chrX:125,068,866-125,141,139 | 72,273 |
| JH584278.1 | chrX:170,672,643-170,678,055 | 5,412 |
| GL456208.2 | chrY:3,289,416-3,429,742 | 140,326 |
| **Total** |  | **20,785,352** |
