# Supplementary material for: BAIT: Organizing genomes and mapping rearrangements in single cells
Source: Genome Med. 2013 Sep 13;5(9):82. doi: 10.1186/gm486 (PMC3971352; doi:10.1186/gm486)
Supplement: Additional file 7 — Supplemental Data File 2 is the Bioinformatic Analysis of Inherited Templates (BAIT) output of correlative data for all GRCm38/mm10 unlocalized scaffolds. Standard output attempts to localize every genomic orphan scaffold by calculating the concordance of template strand inheritance across all libraries within each dataset (for an example, see Figure 4 for mapping of chrUn_GL456239). Each ideogram plot shows the likeliest location of the scaffold (red region), the percentage concordance (agreement between libraries), and the number of libraries used in the analysis. [file gm486-S7.pdf]

Fragment= chrUn_GL456239 Organism= Mus_musculus **Peak agreement= 91.07 %**

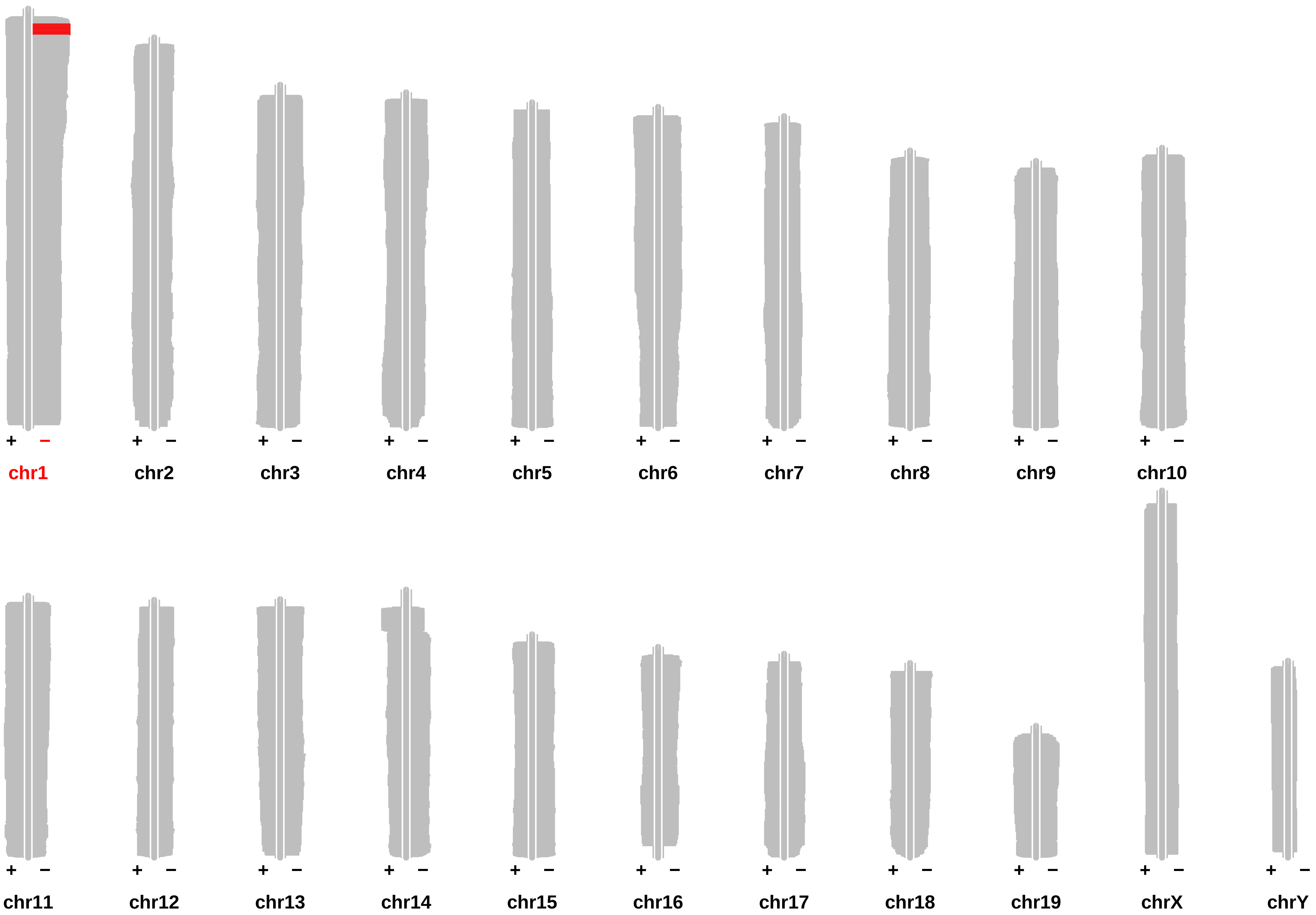

Quality filter=20

Fragment= chrUn_GL456359 Organism= Mus_musculus Peak agreement= 86.79 %

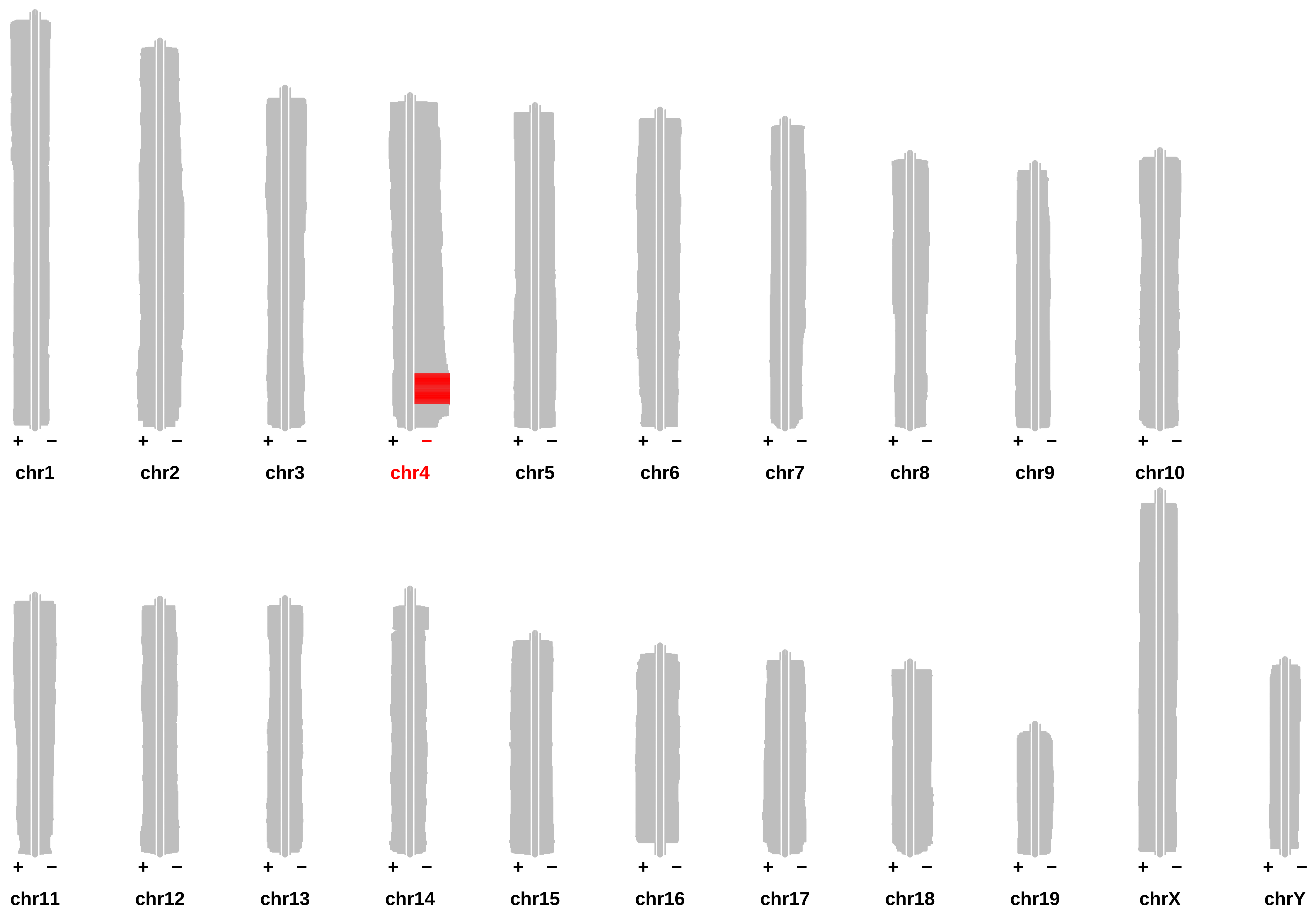

Quality filter=20

Fragment= chrUn_GL456360 Organism= Mus_musculus Peak agreement= 89.09 %

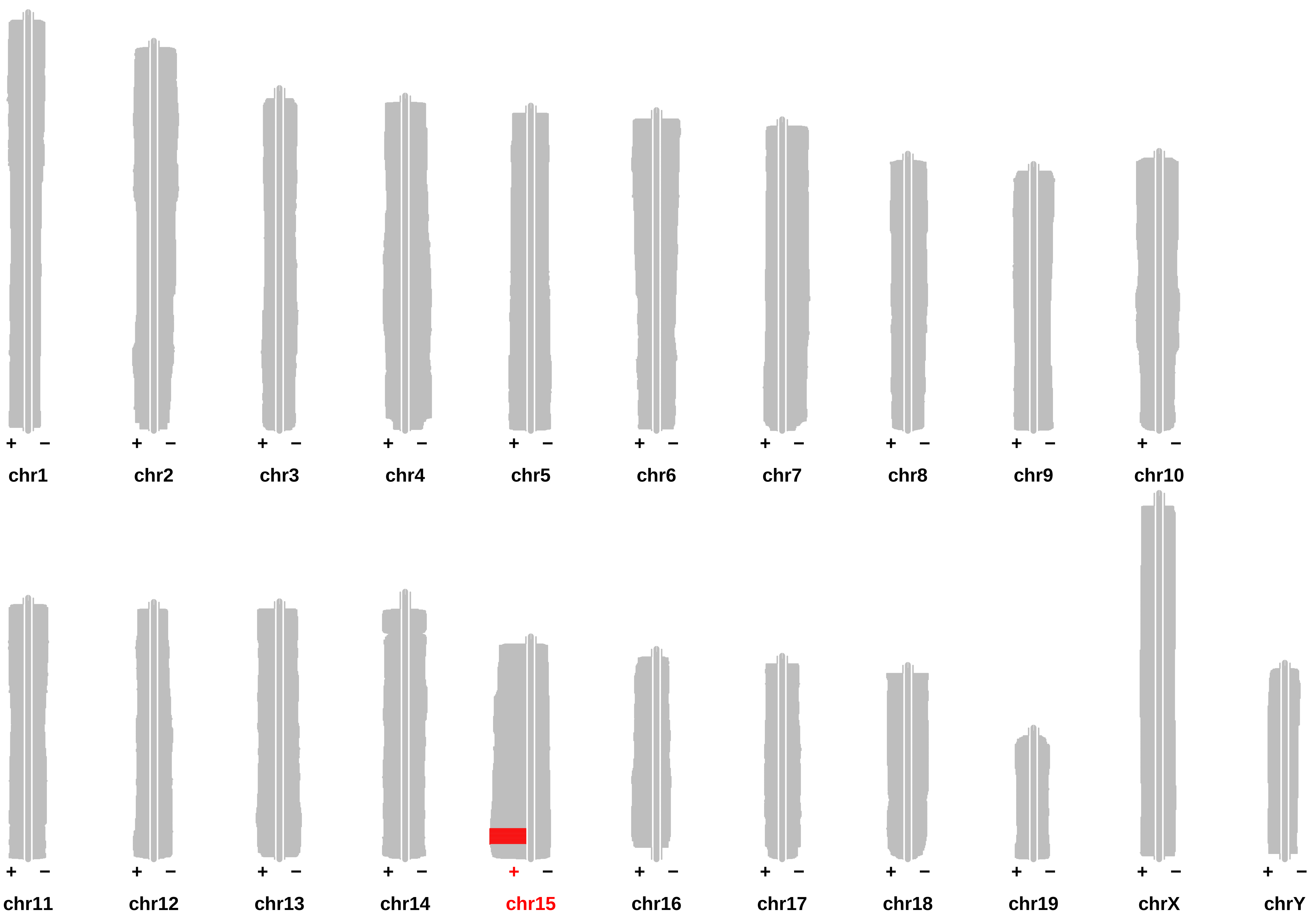

Quality filter=20

Fragment= chrUn_GL456366 Organism= Mus_musculus Peak agreement= 82.14 %

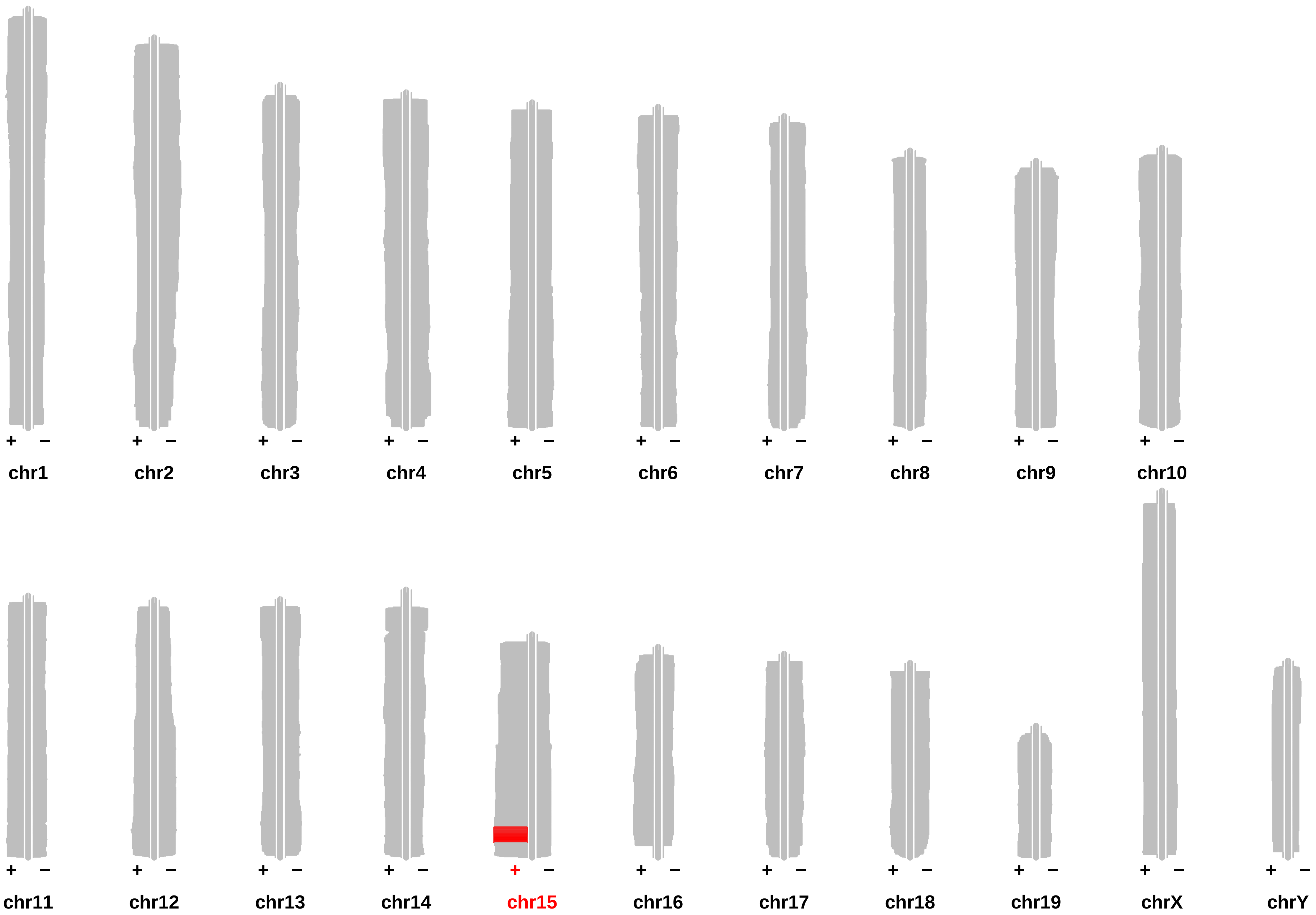

Quality filter=20

Fragment= chrUn_GL456367 Organism= Mus_musculus **Peak agreement= 95 %**

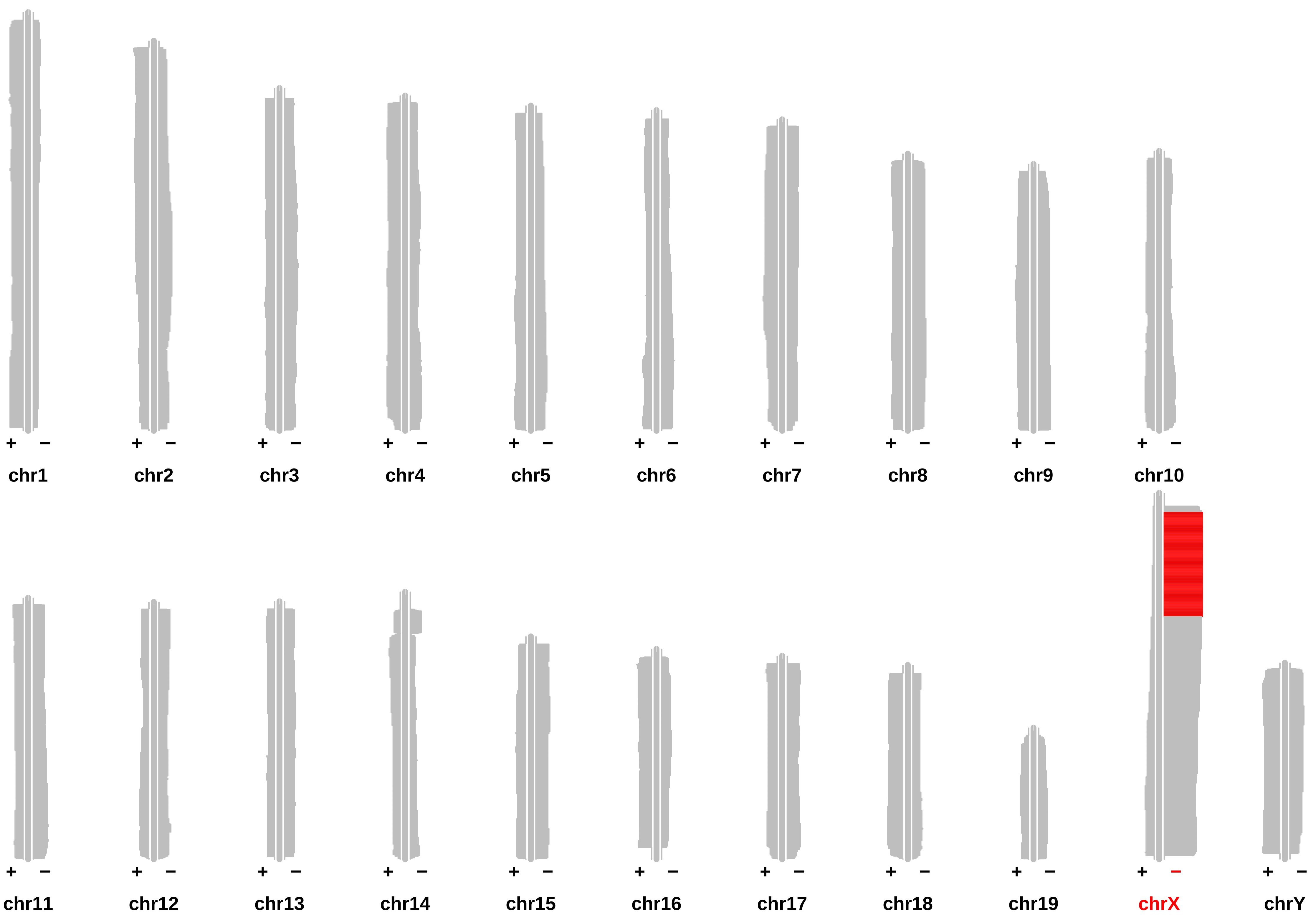

Quality filter=20

Fragment= chrUn_GL456368 Organism= Mus_musculus Peak agreement= 84.09 %

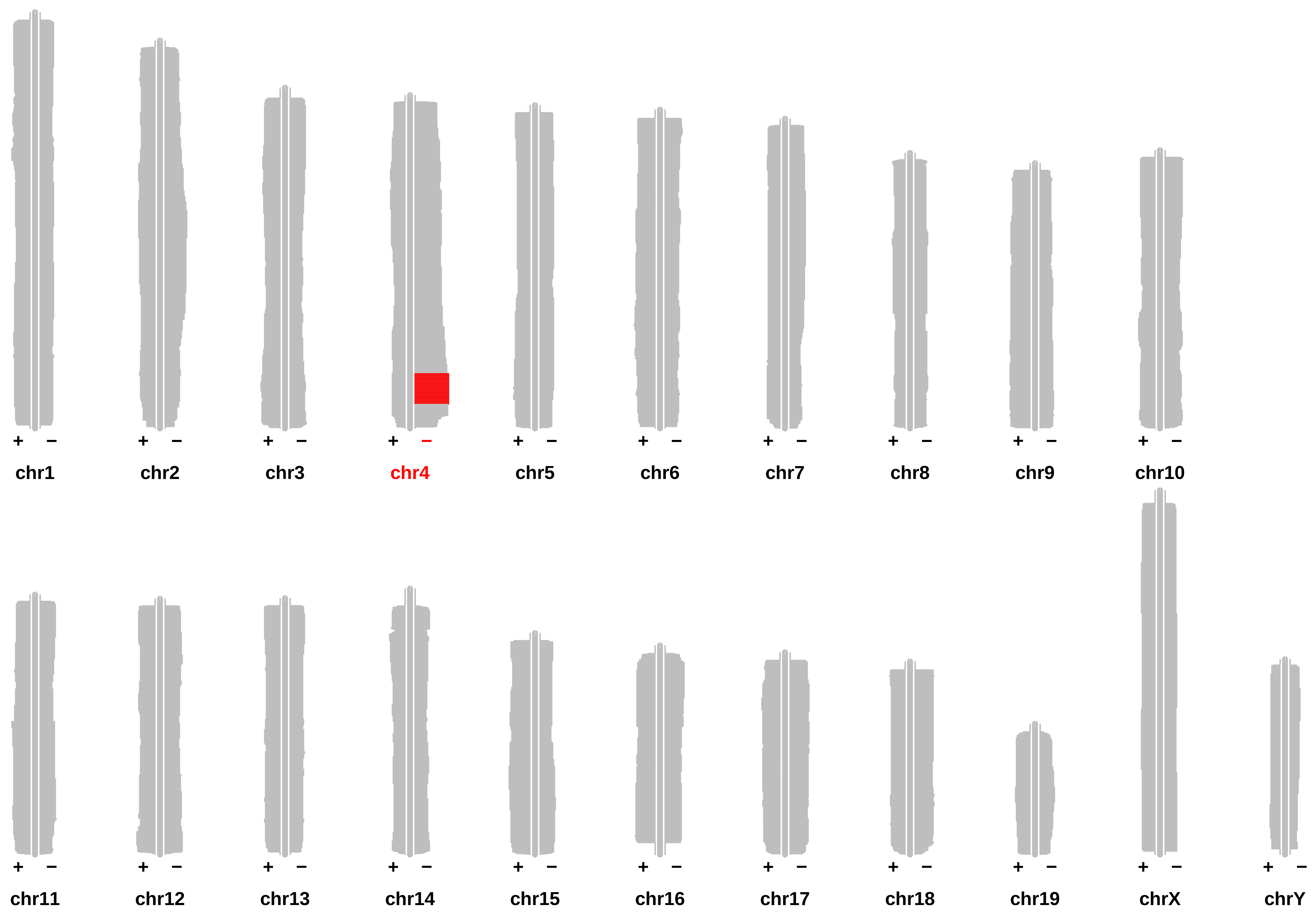

Quality filter=20

Fragment= chrUn_GL456370 Organism= Mus_musculus Peak agreement= 52.38 %

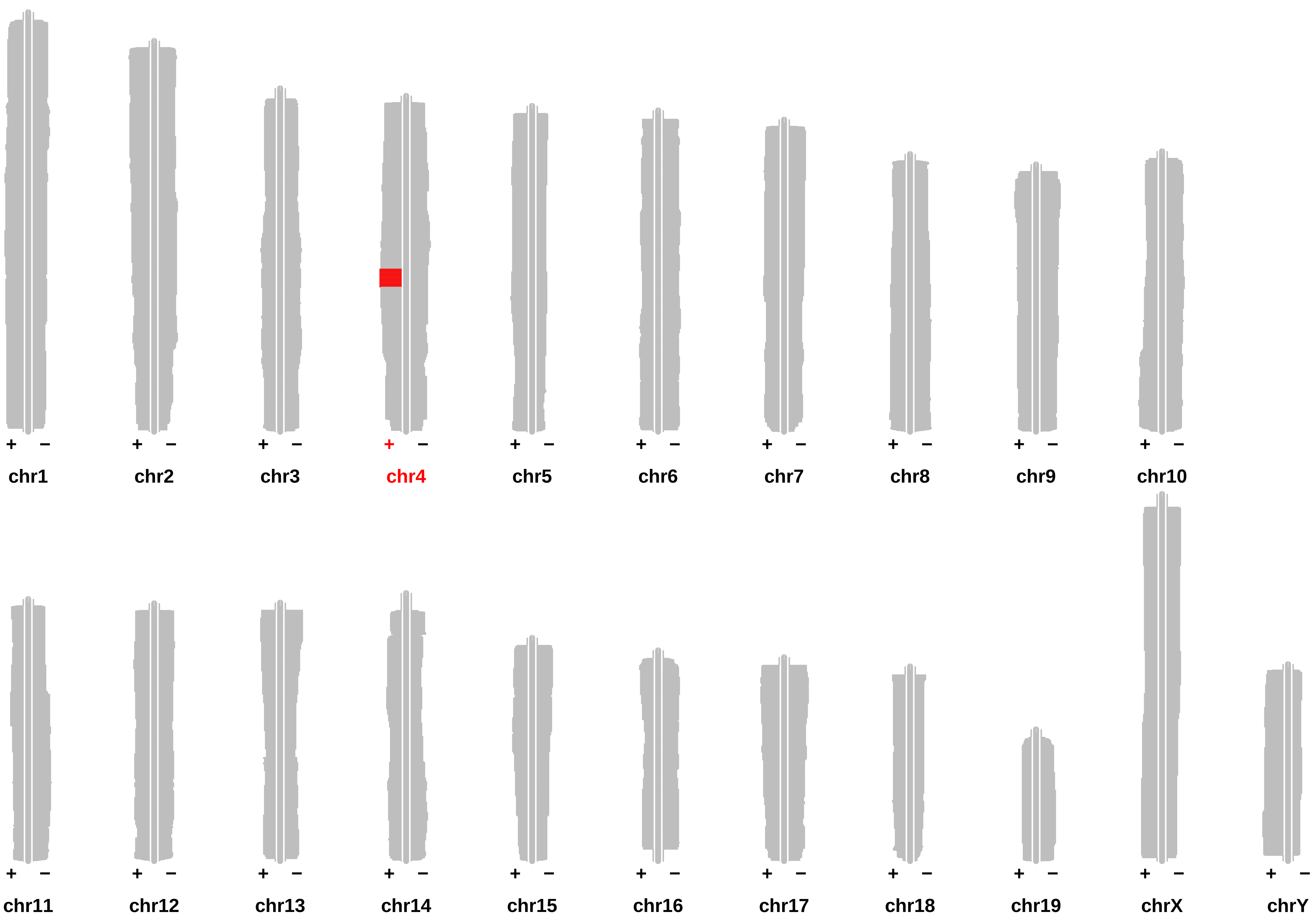

Quality filter=20

Fragment= chrUn_GL456372 Organism= Mus_musculus Peak agreement= 69.81 %

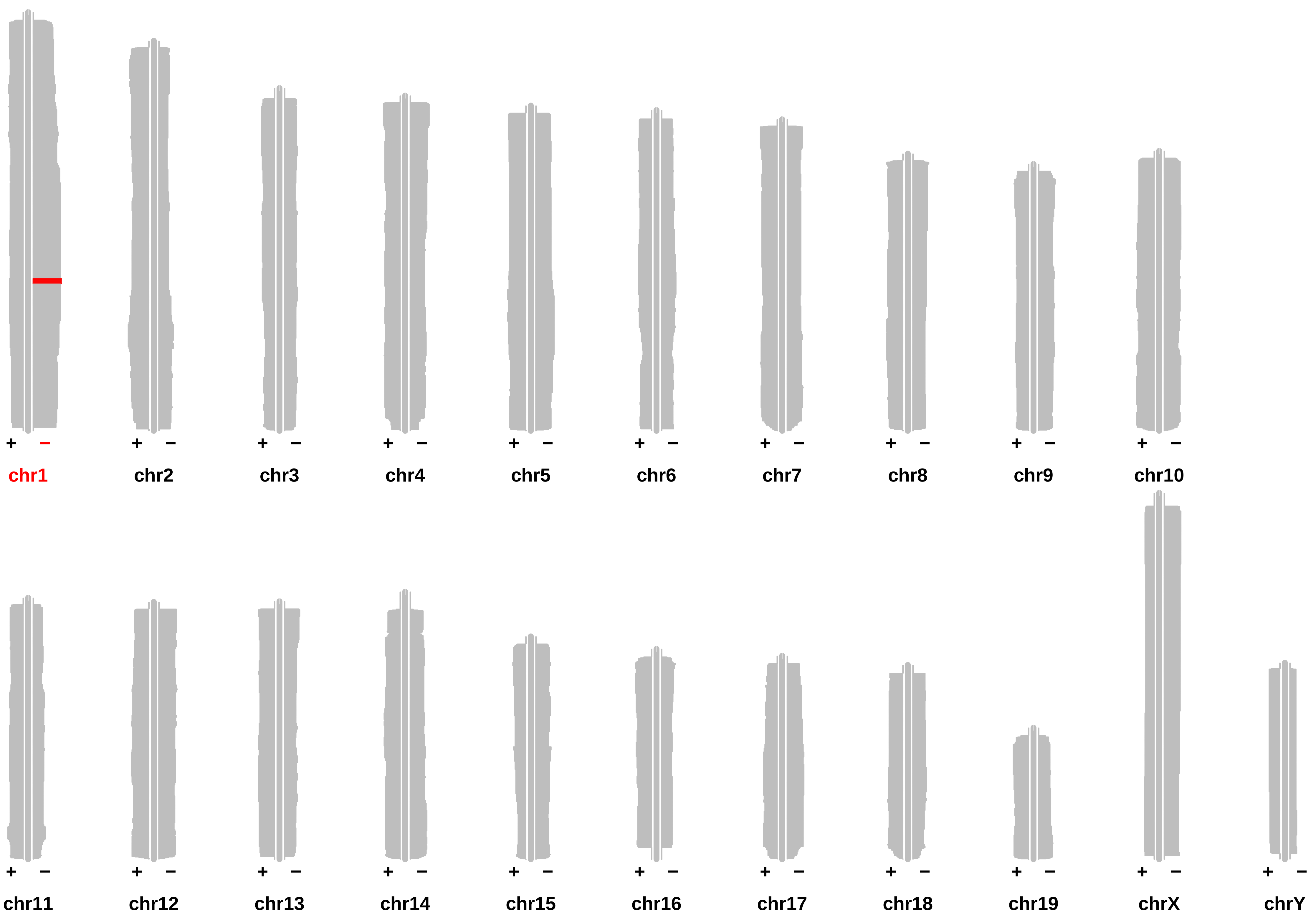

Quality filter=20

Fragment= chrUn_GL456378 Organism= Mus_musculus Peak agreement= 63.16 %

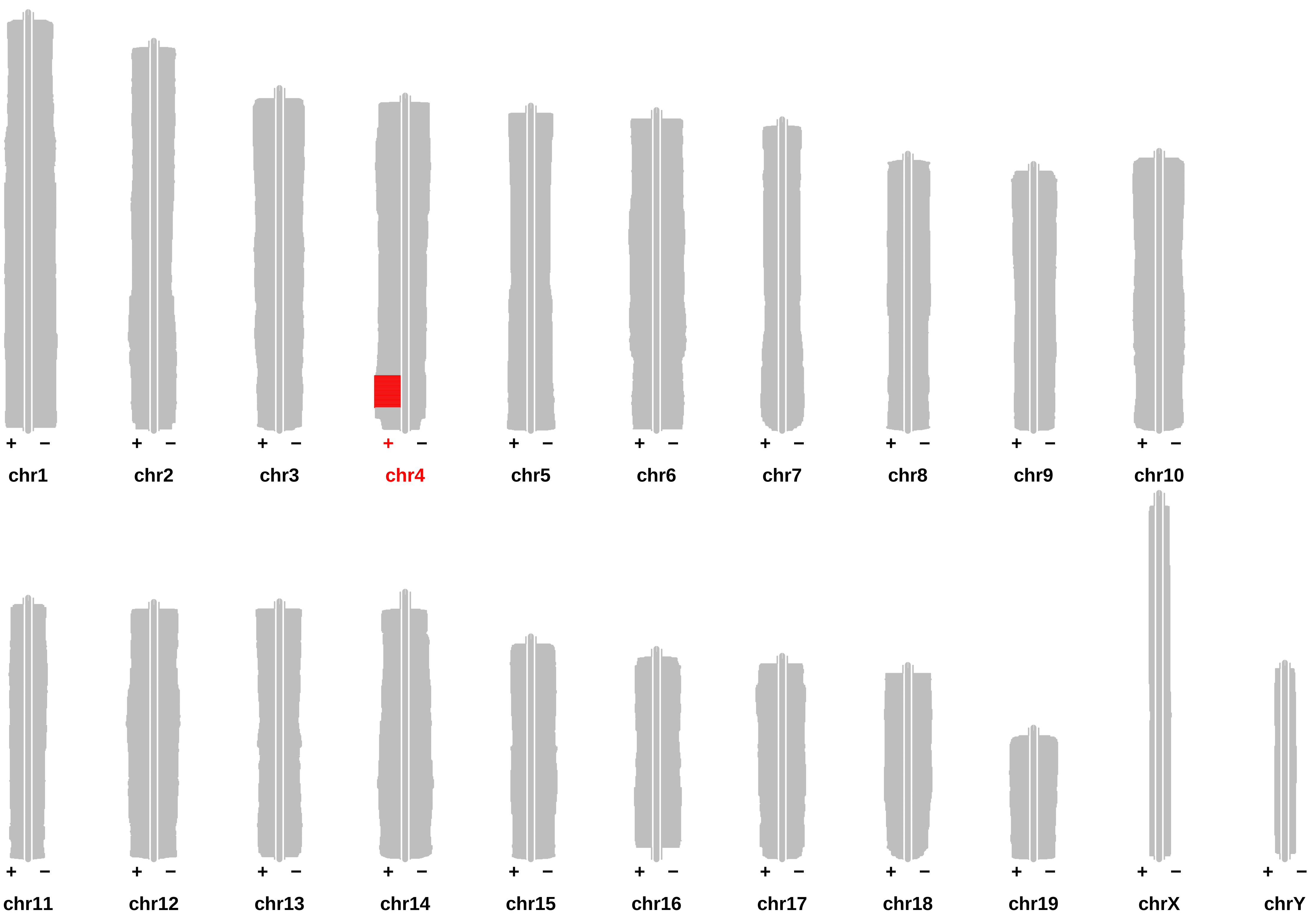

Quality filter=20

Fragment= chrUn_GL456379 Organism= Mus_musculus **Peak agreement= 97.83 %**

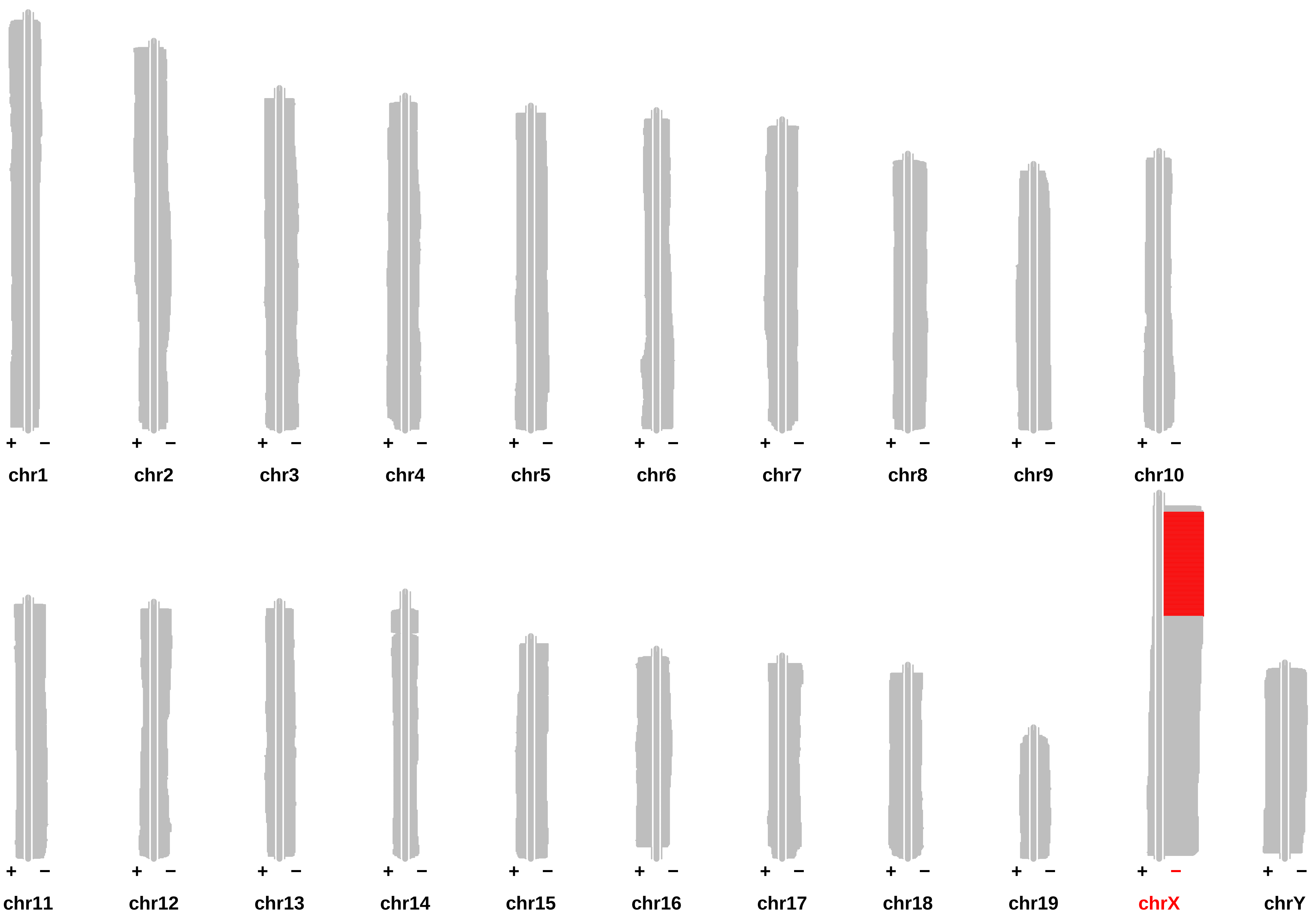

Quality filter=20

Fragment= chrUn_GL456381 Organism= Mus_musculus Peak agreement= 93.88 %

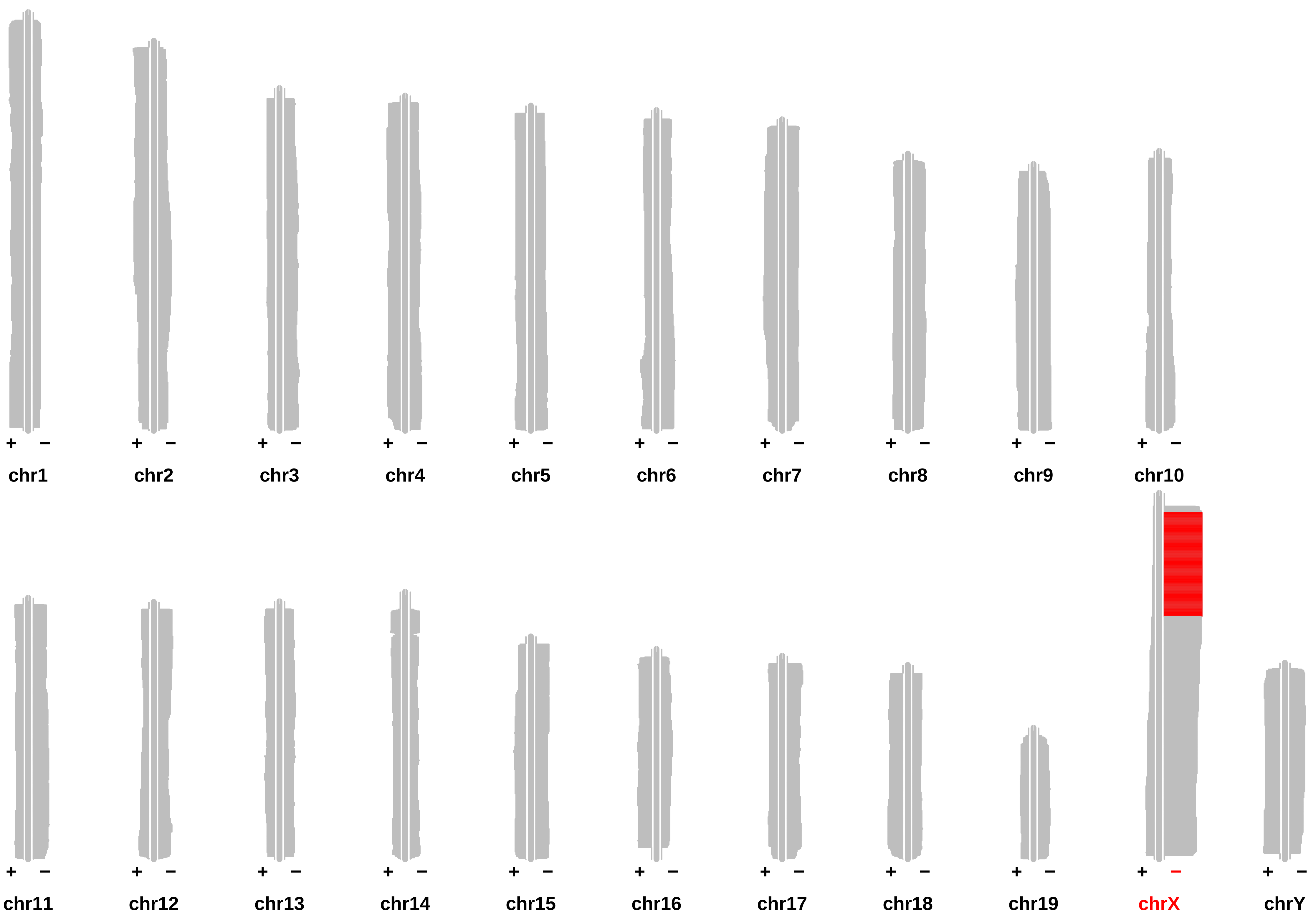

Quality filter=20

Fragment= chrUn_GL456382 Organism= Mus_musculus Peak agreement= 95.24 %

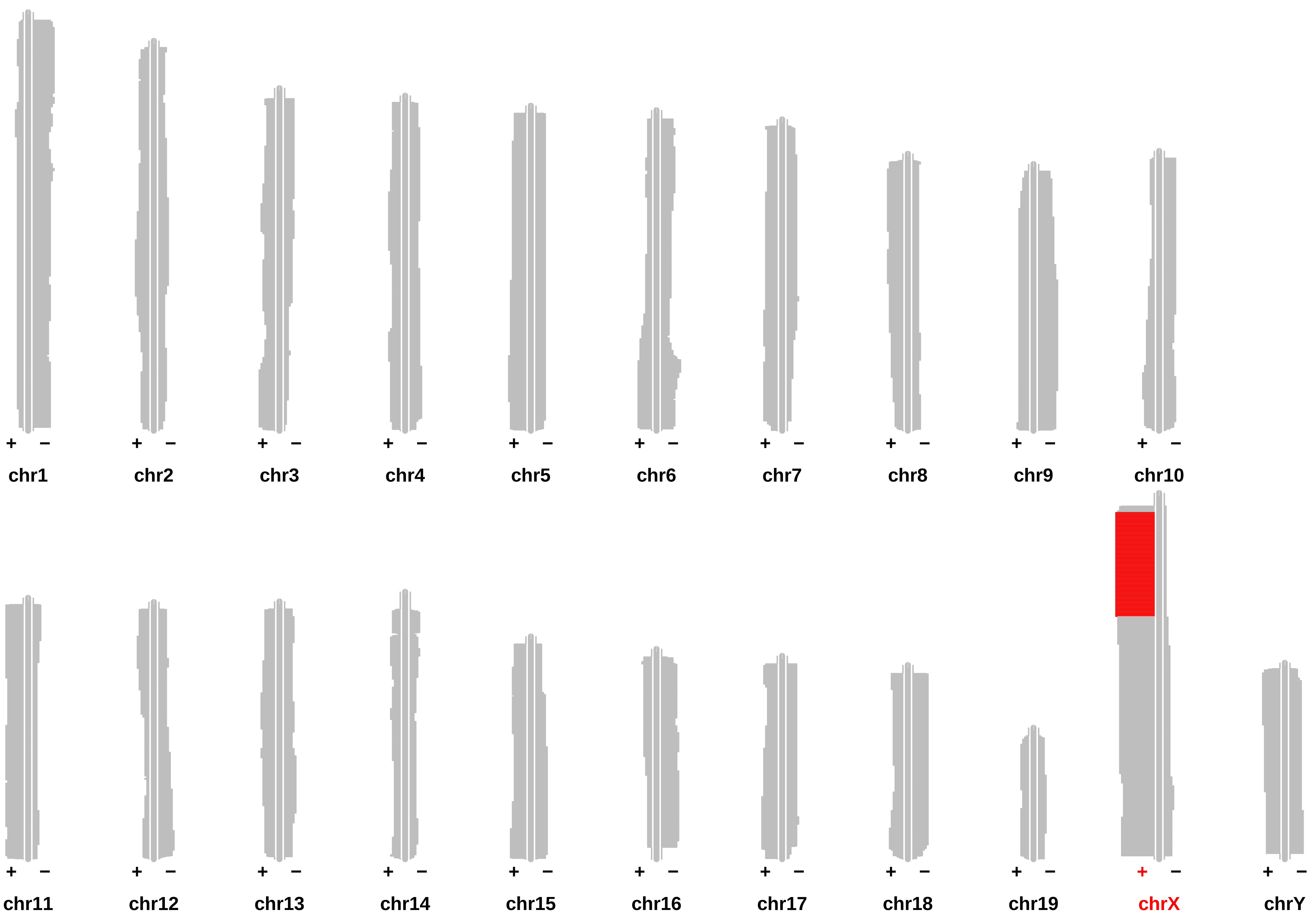

Quality filter=20

Fragment= chrUn_GL456385 Organism= Mus_musculus Peak agreement= 82.69 %

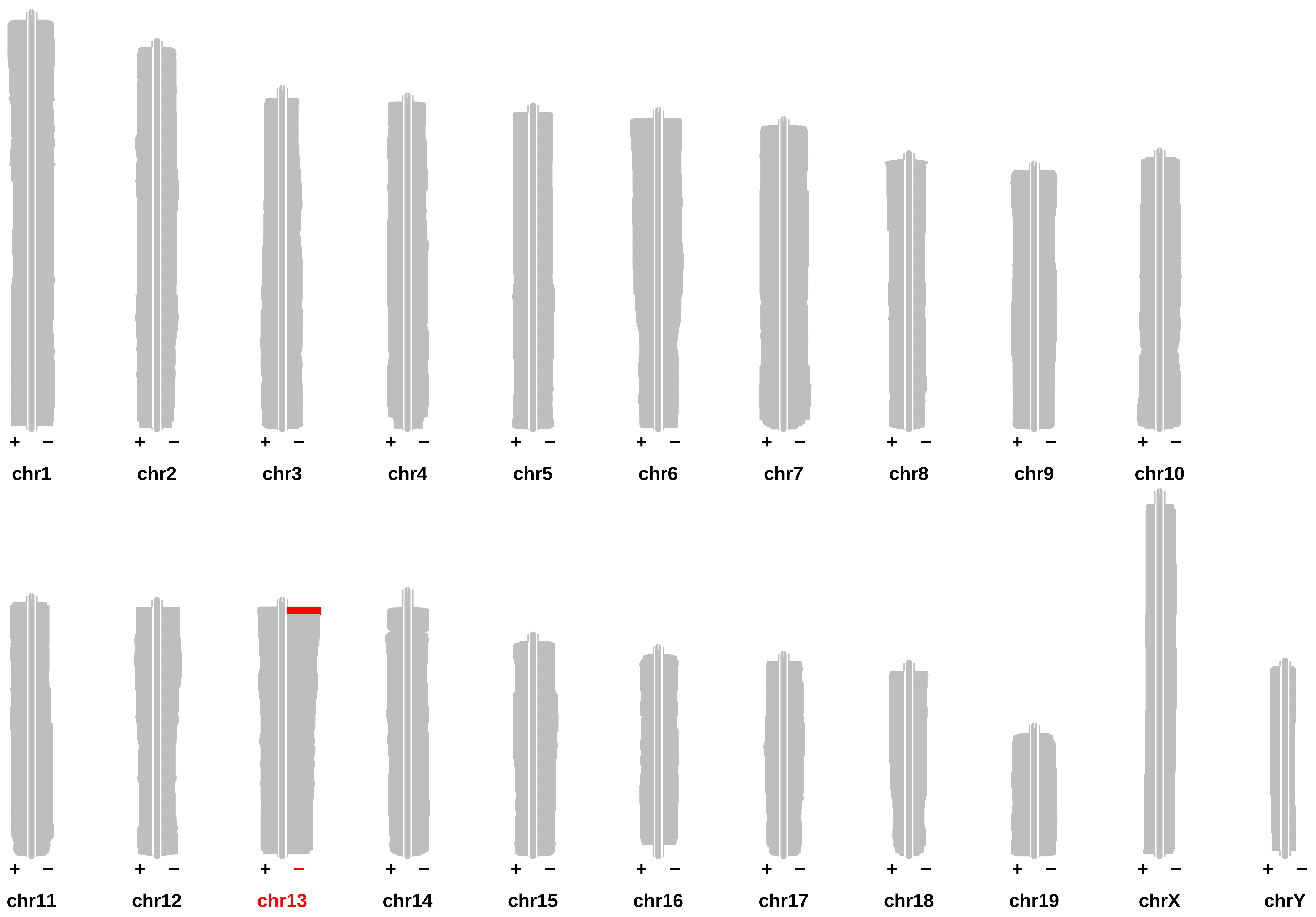

Quality filter=20

Fragment= chrUn_GL456387 Organism= Mus_musculus Peak agreement= 71.93 %

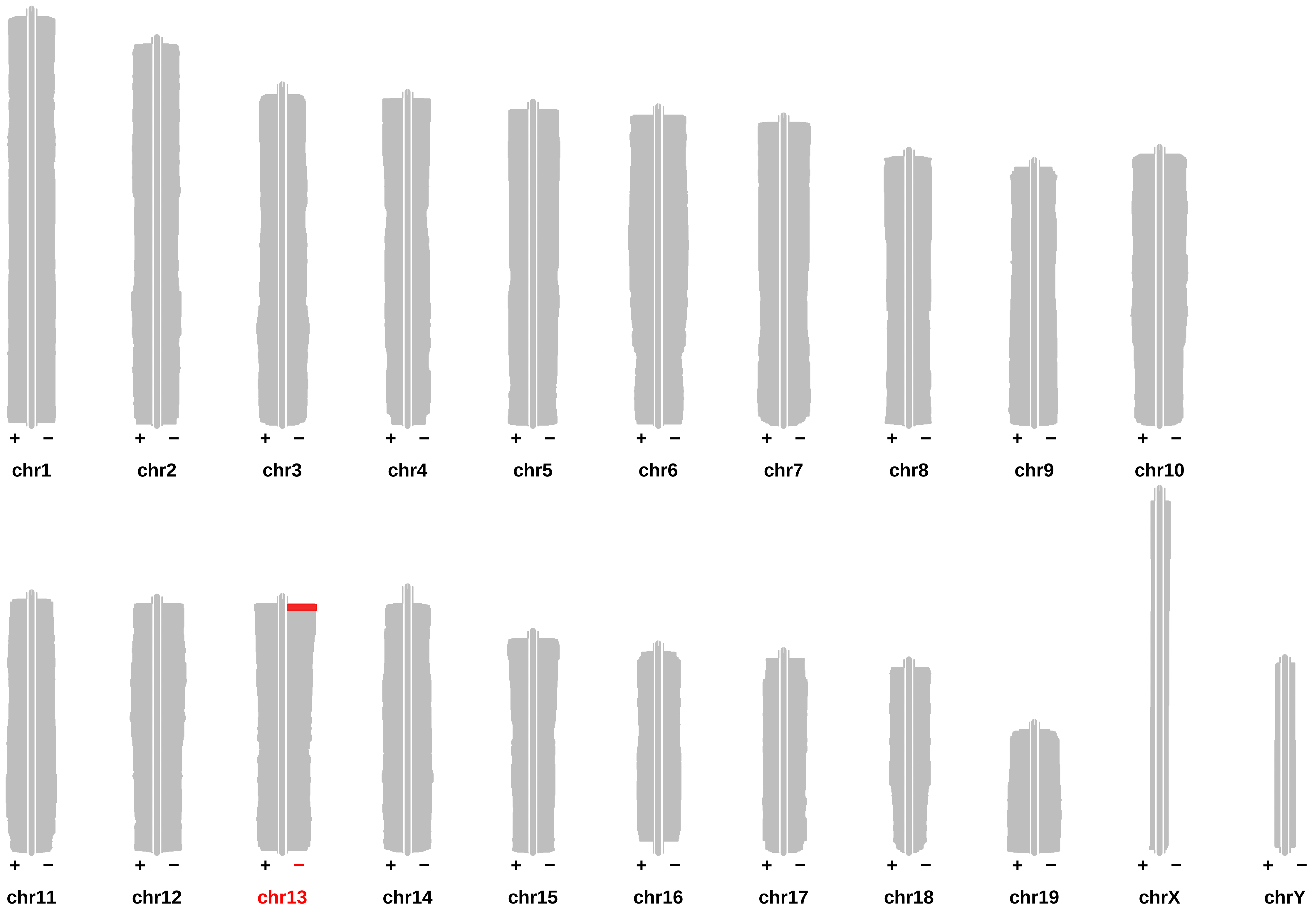

Quality filter=20

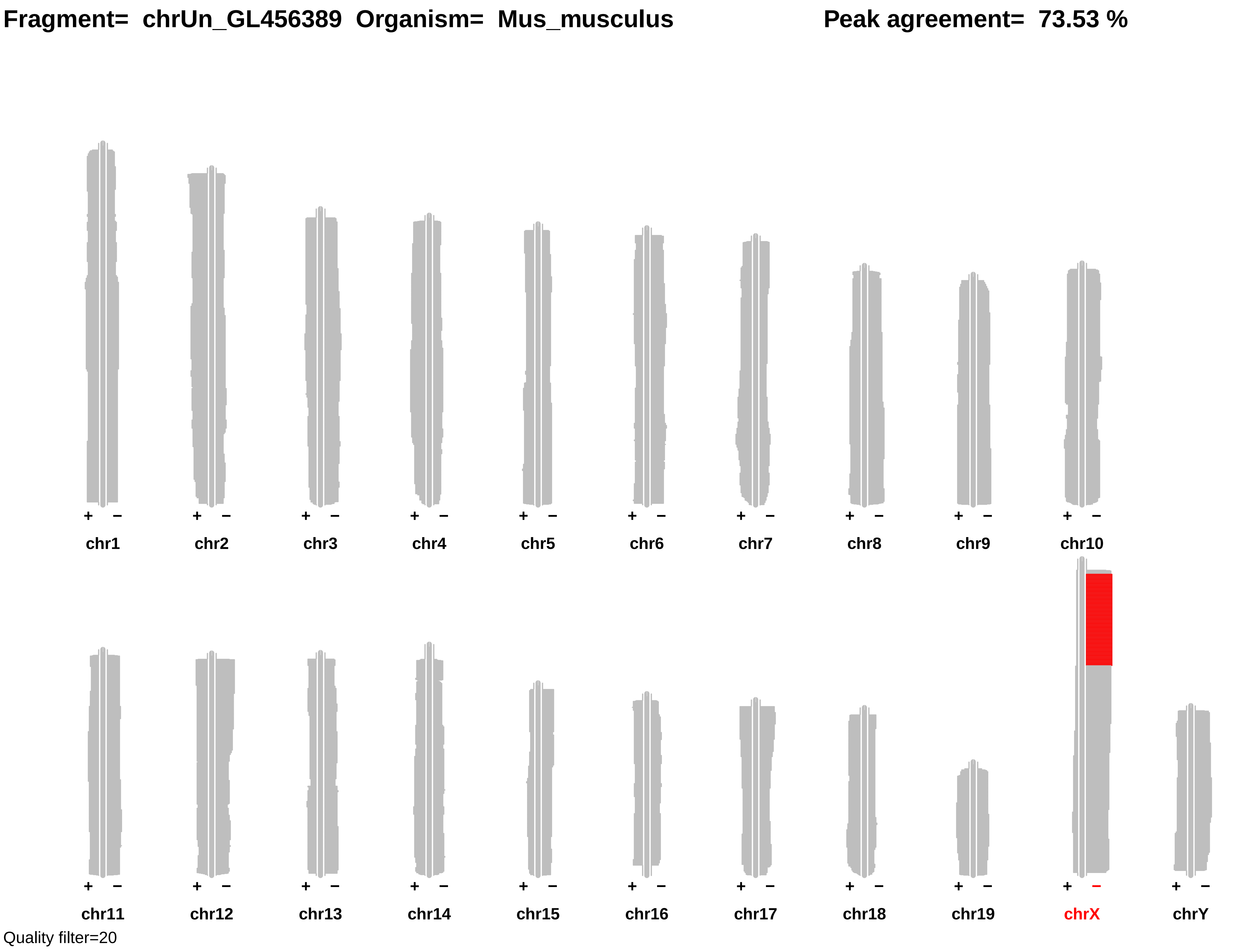

Fragment= chrUn_GL456389 Organism= Mus_musculus
Peak agreement= 73.53 %
+ −
chr1
chr2
chr3
chr4
chr5
chr6
chr7
chr8
chr9
chr10
chr11
chr12
chr13
chr14
chr15
chr16
chr17
chr18
chr19
chrX
chrY
Quality filter=20

Fragment= chrUn_GL456392 Organism= Mus_musculus Peak agreement= 79.41 %

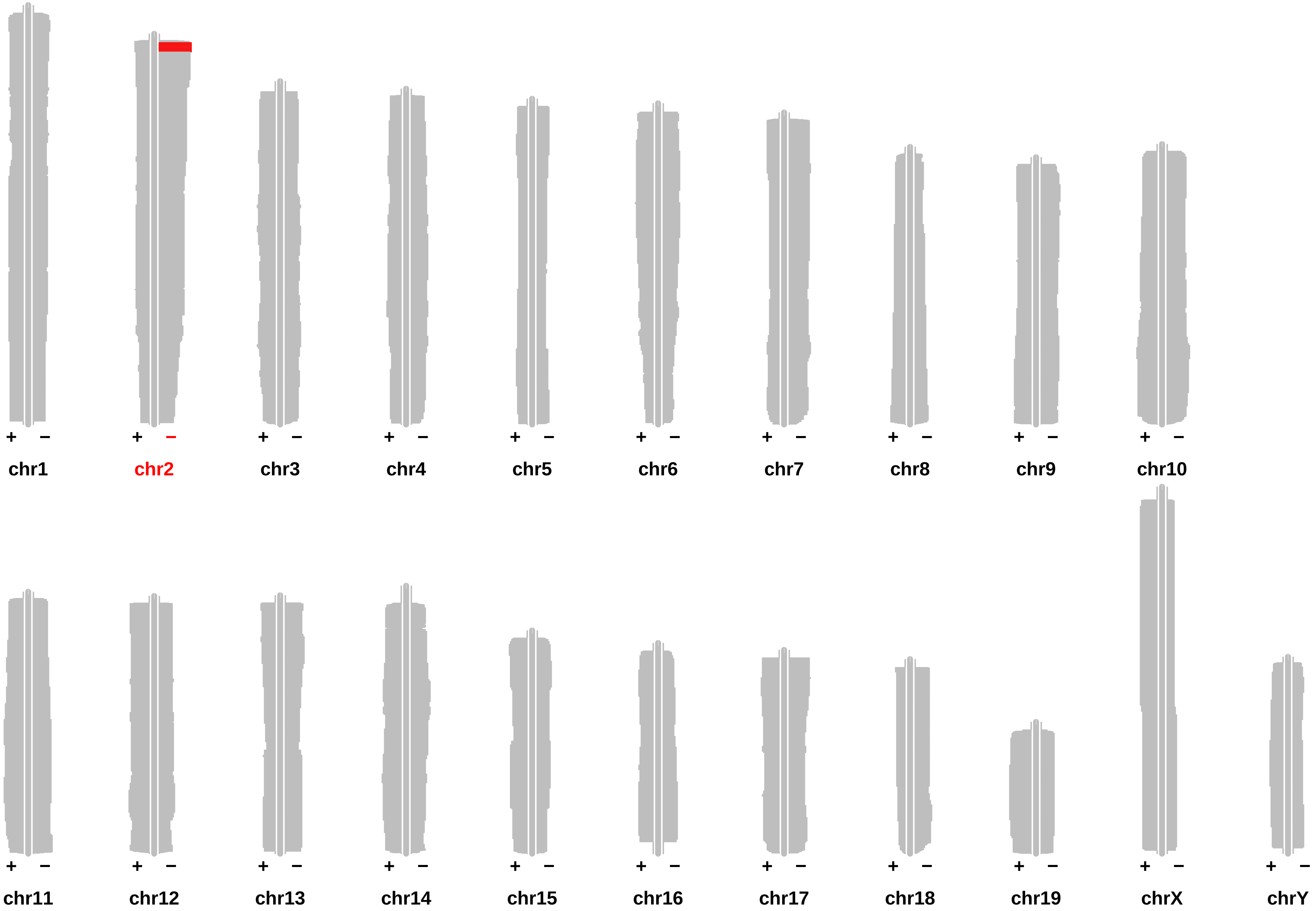

Quality filter=20

Fragment= chrUn_GL456393 Organism= Mus_musculus Peak agreement= 89.29 %

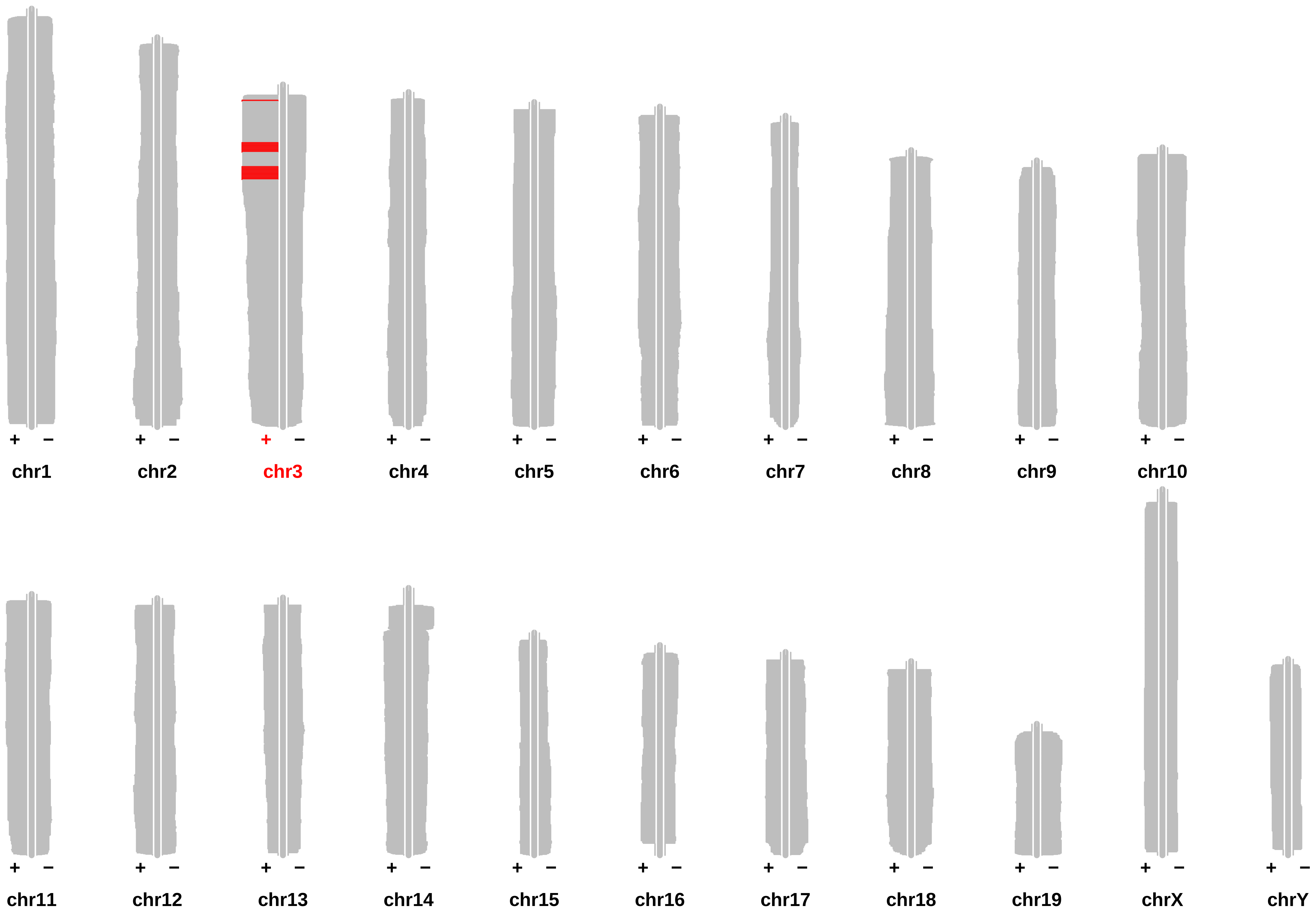

Quality filter=20

Fragment= chrUn_GL456394 Organism= Mus_musculus Peak agreement= 77.78 %

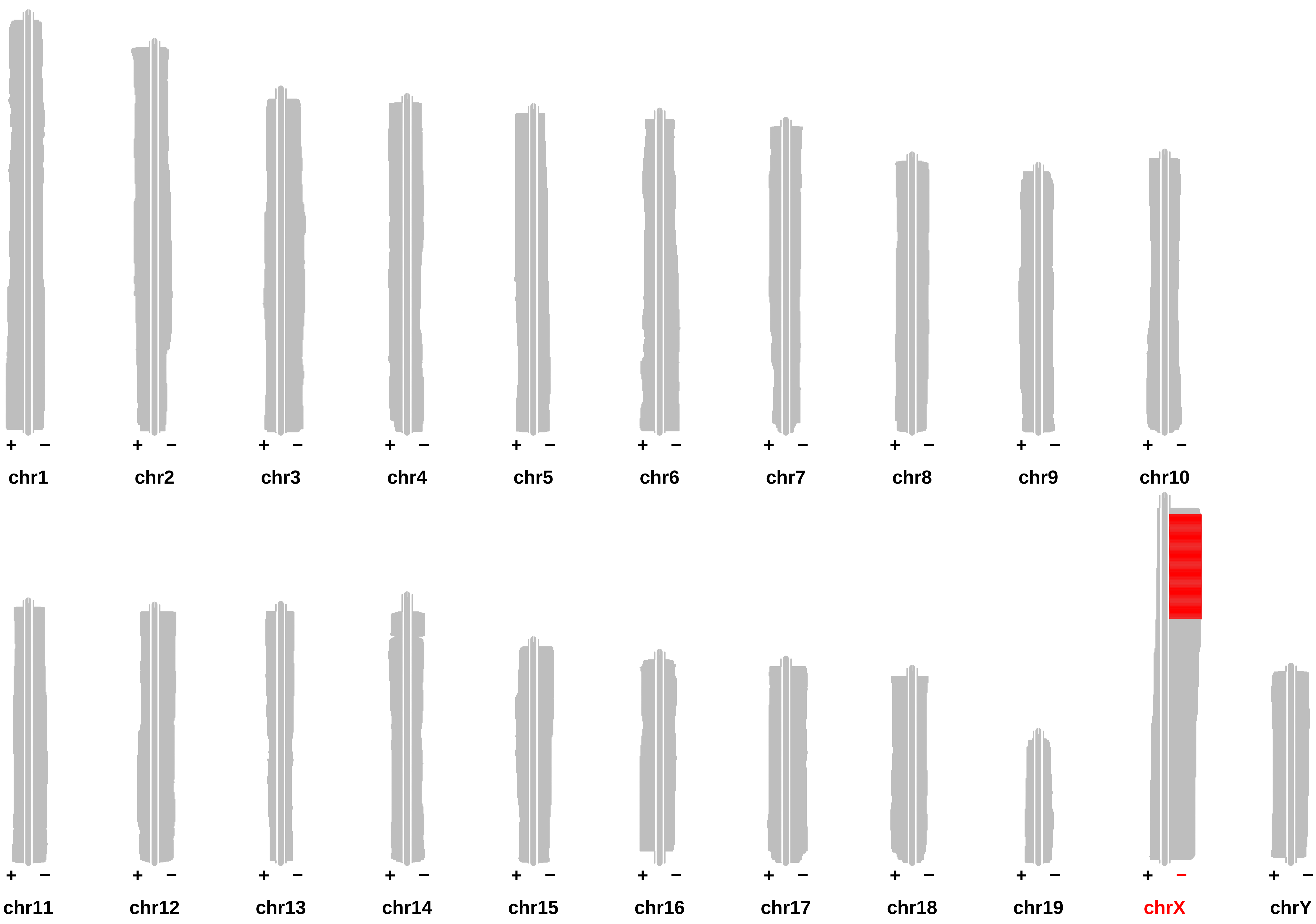

Quality filter=20

Fragment= chrUn_GL456396 Organism= Mus_musculus Peak agreement= 90.48 %

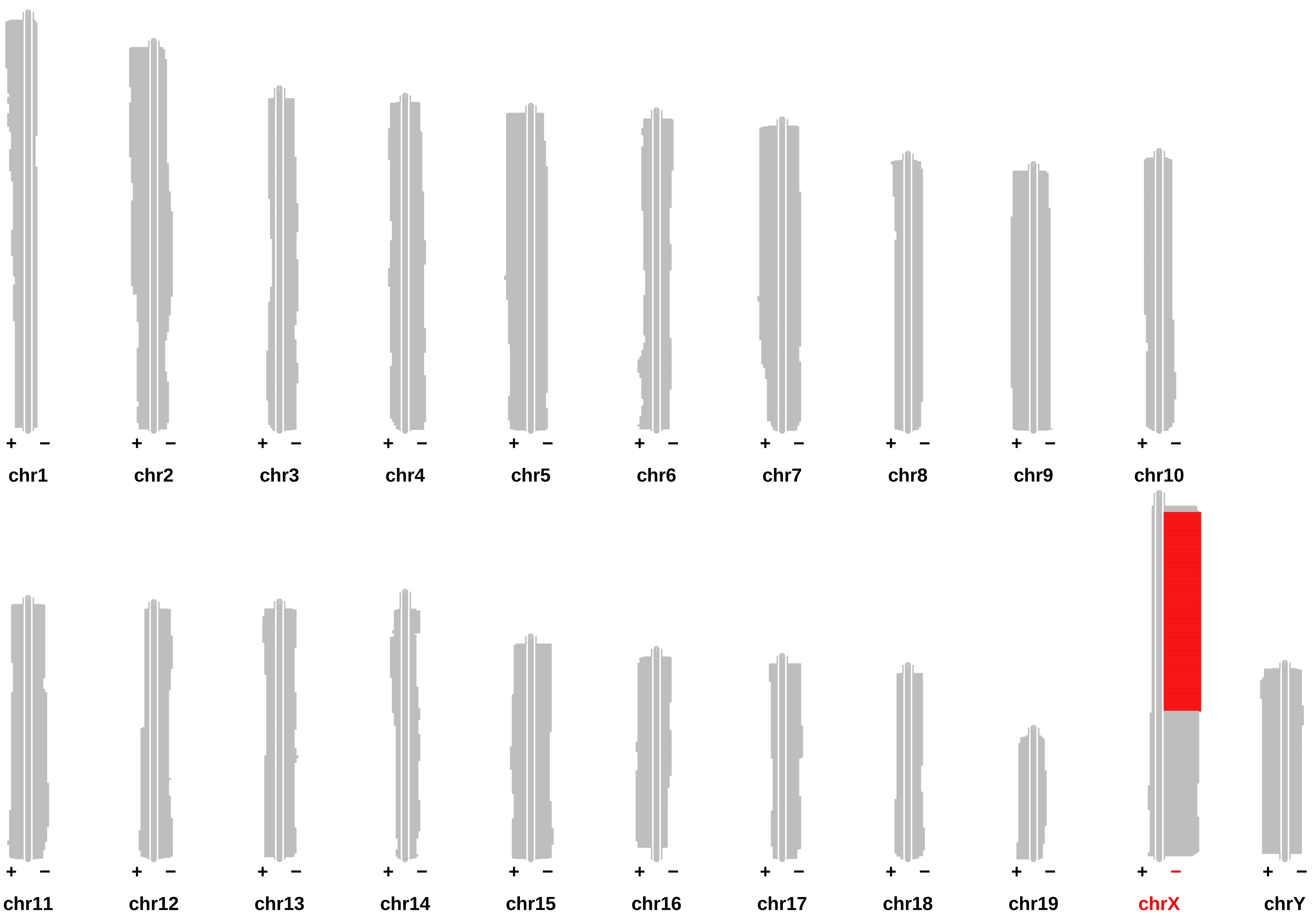

Quality filter=20

Fragment= chrUn_JH584304 Organism= Mus_musculus Peak agreement= 64.91 %

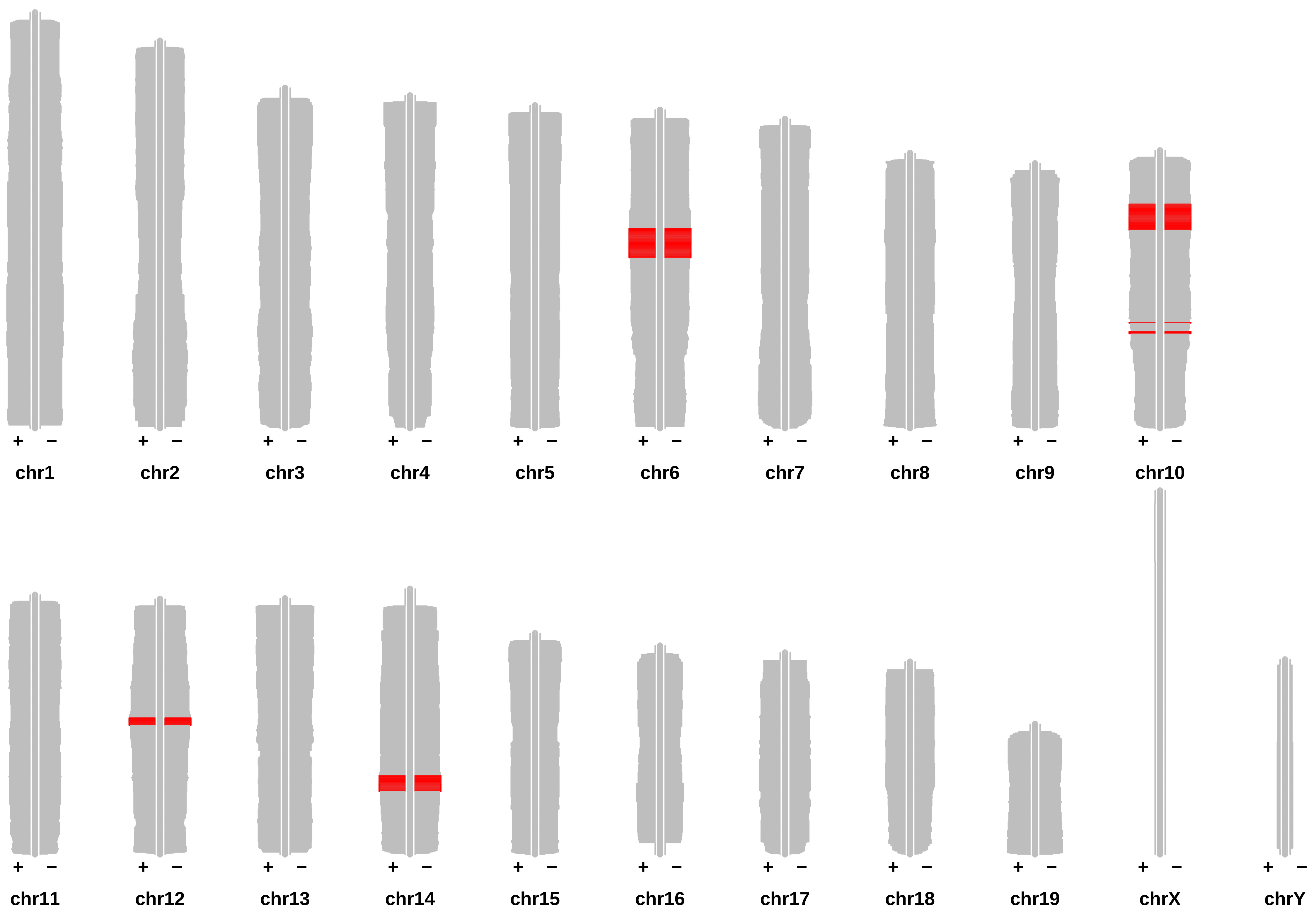

Quality filter=20

Fragment= chrX_GL456233_random Organism= Mus_musculus Peak agreement= 96.08 %

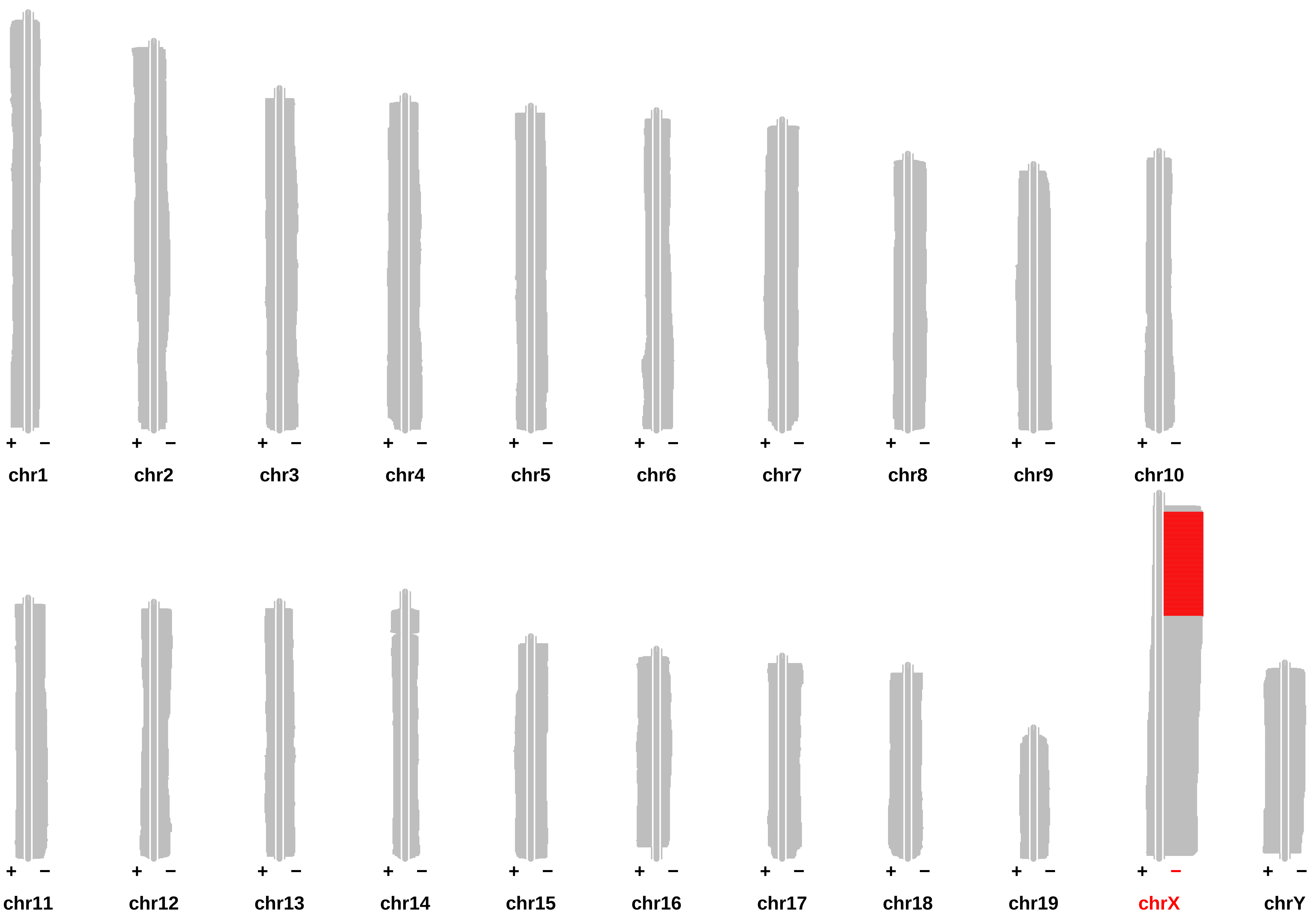

Quality filter=20

Fragment= chr1_GL456210_random Organism= Mus_musculus Peak agreement= 77.36 %

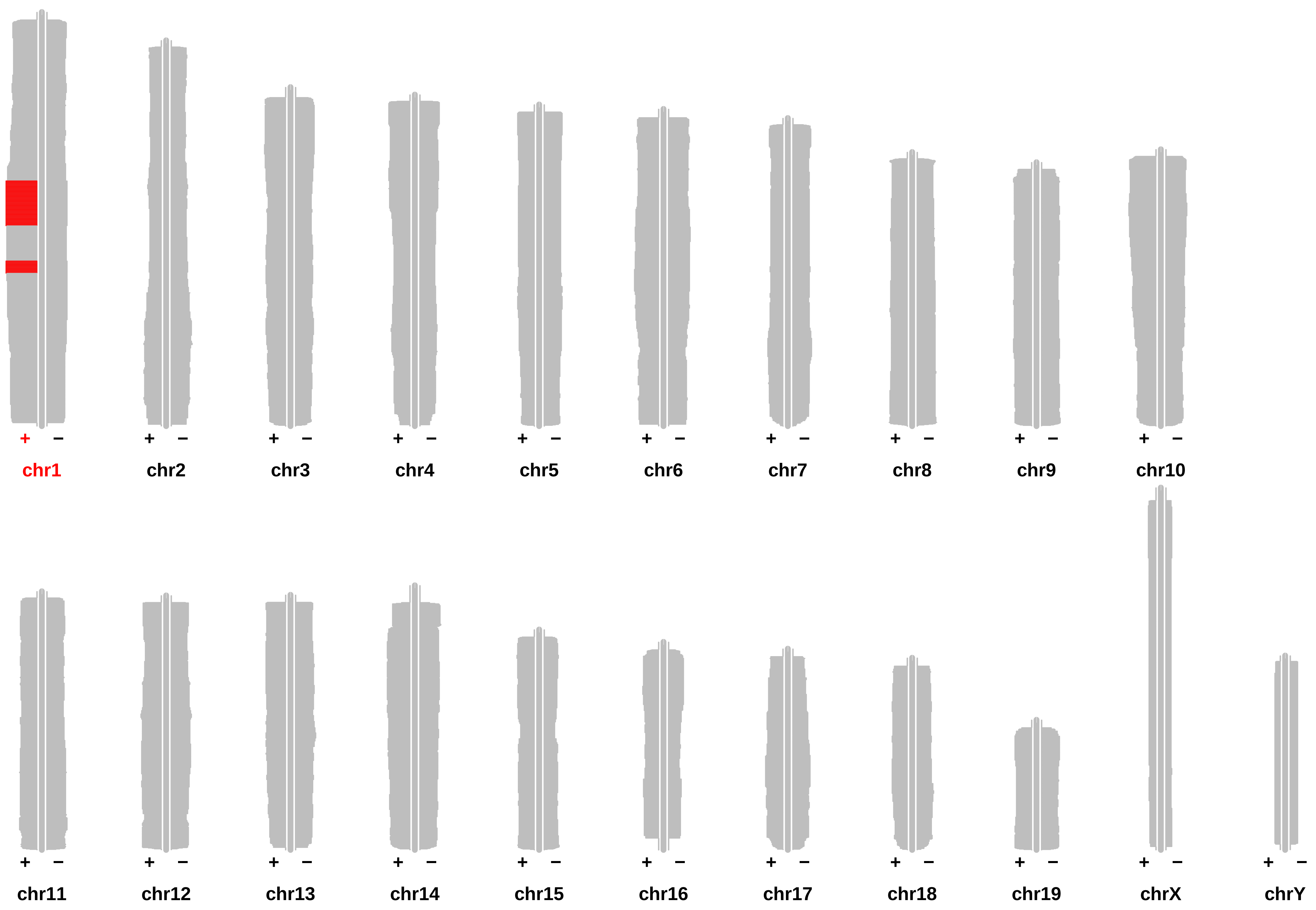

Quality filter=20

Fragment= chr1_GL456211_random Organism= Mus_musculus Peak agreement= 63.16 %

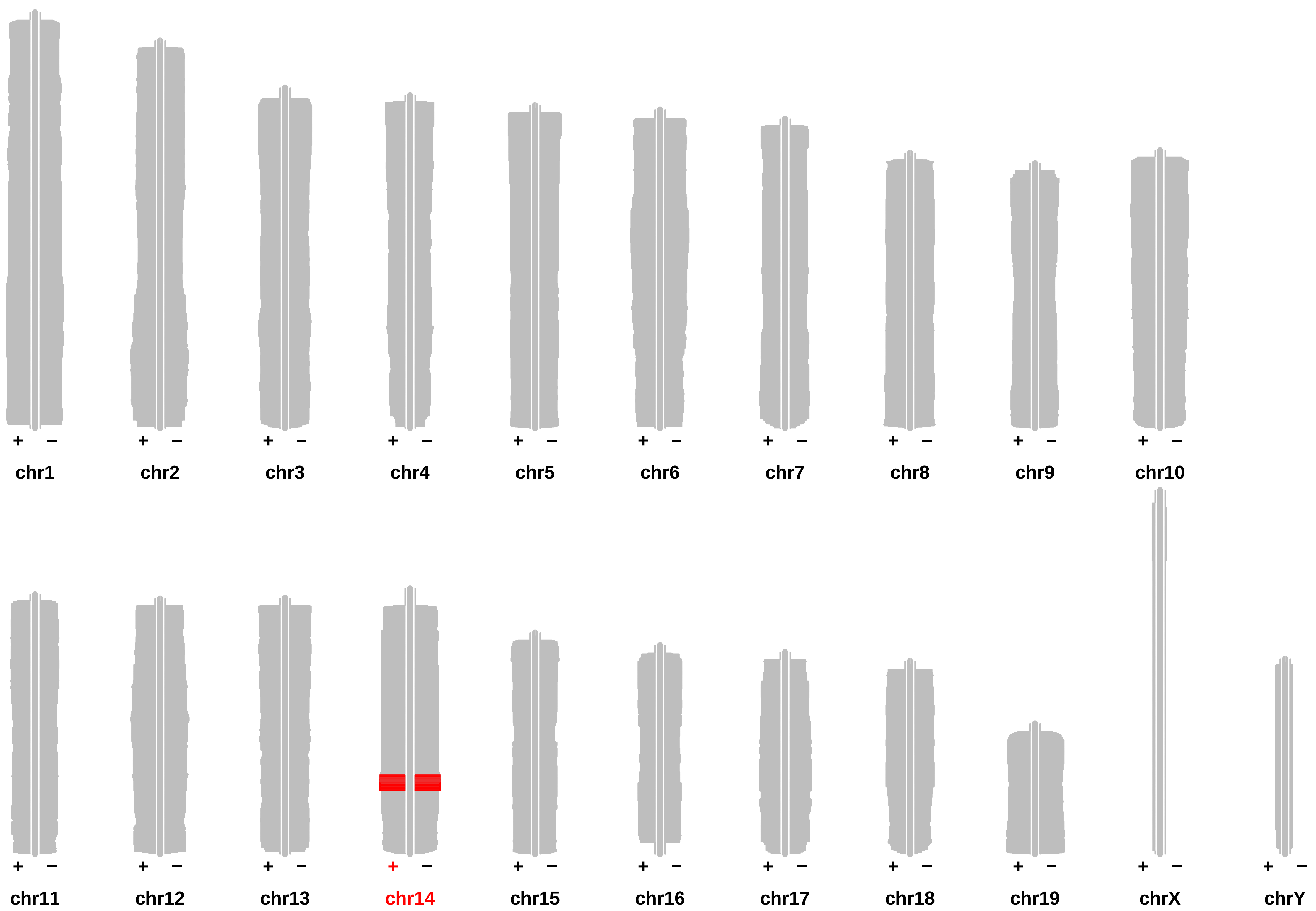

Quality filter=20

Fragment= chr1_GL456212_random Organism= Mus_musculus Peak agreement= 64.81 %

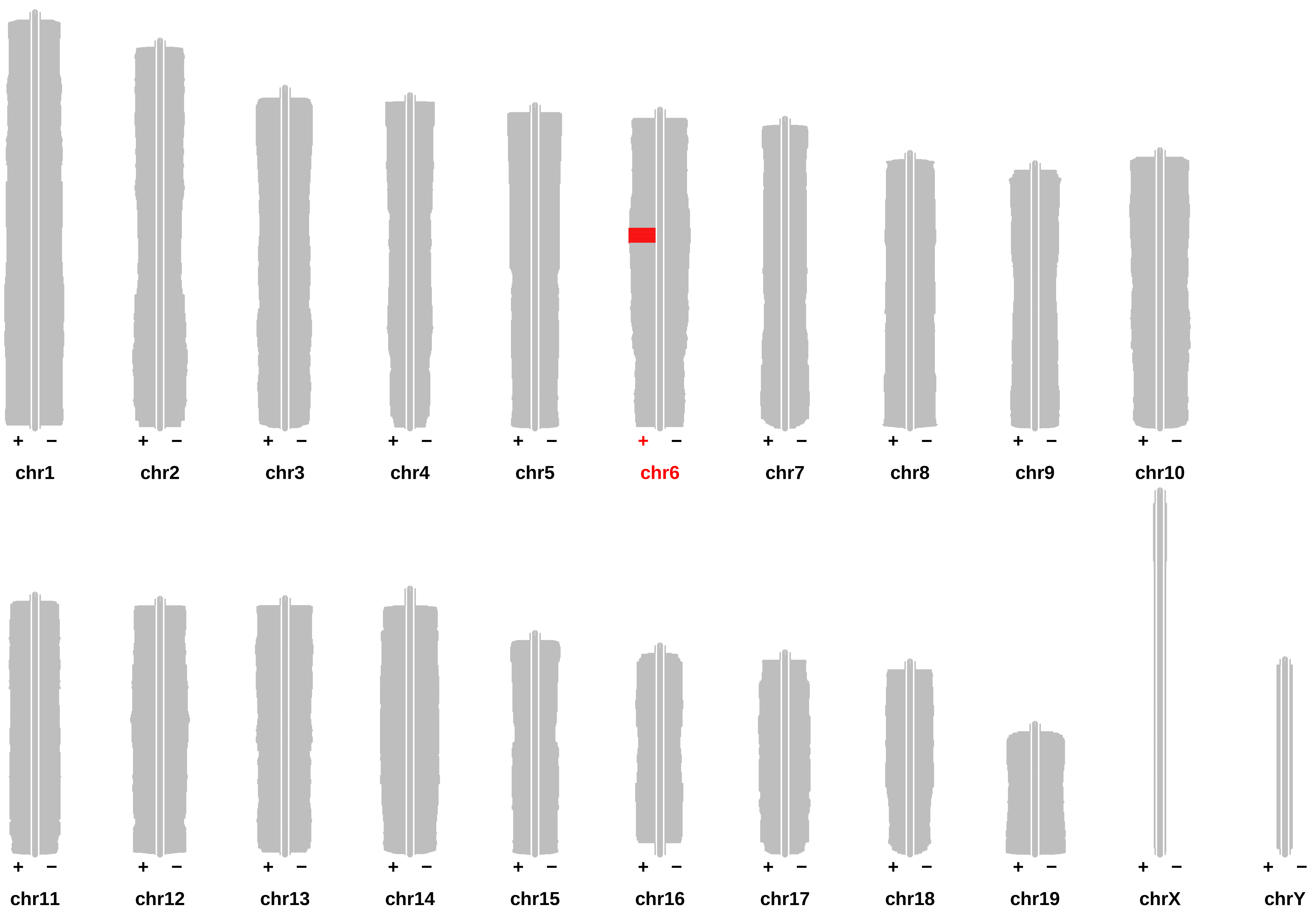

Quality filter=20

Fragment= chr1_GL456221_random Organism= Mus_musculus Peak agreement= 78.57 %

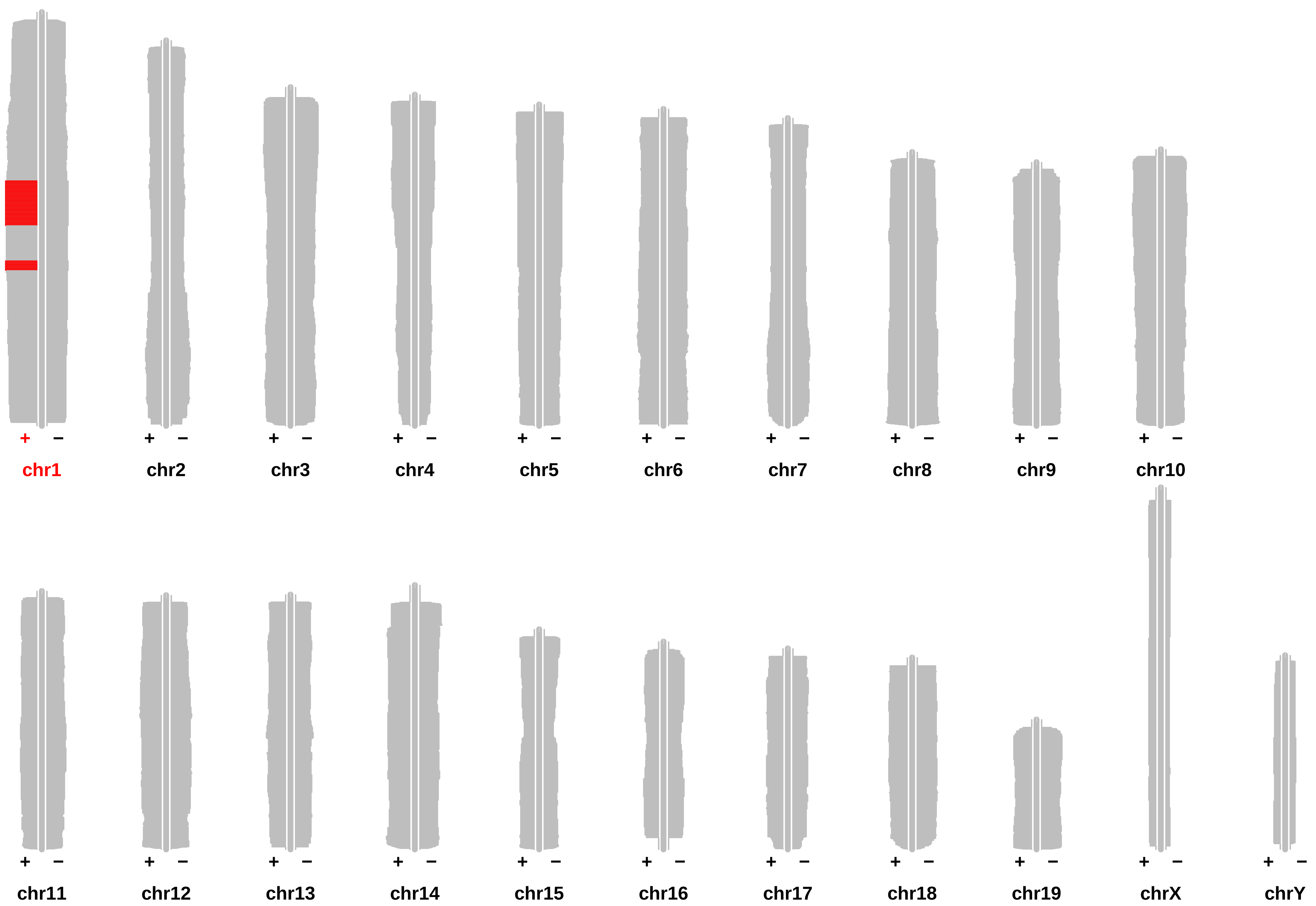

Quality filter=20

Fragment= chr4_JH584292_random Organism= Mus_musculus Peak agreement= 72.55 %

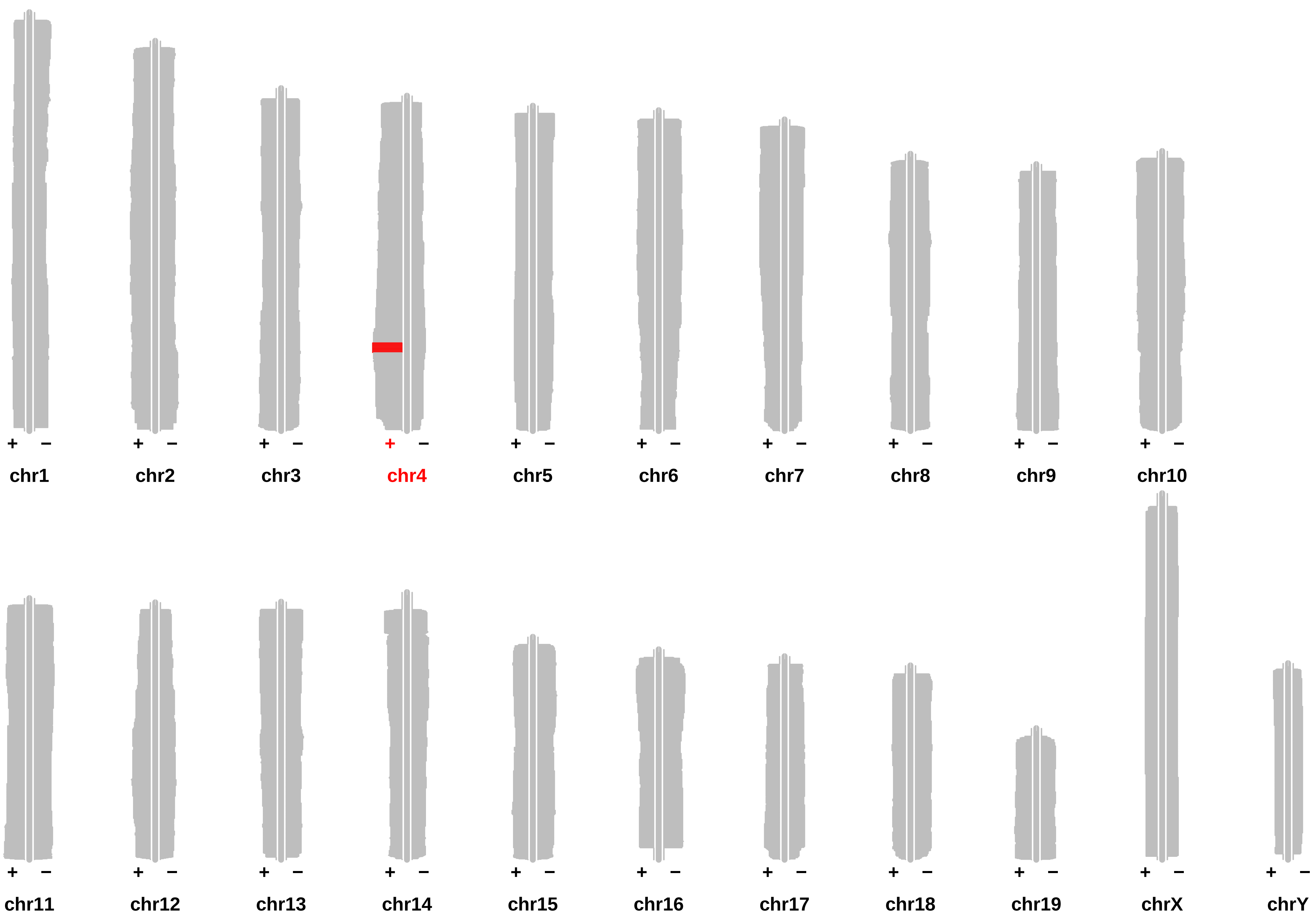

Quality filter=20

Fragment= chr5_GL456354_random Organism= Mus_musculus Peak agreement= 73.08 %

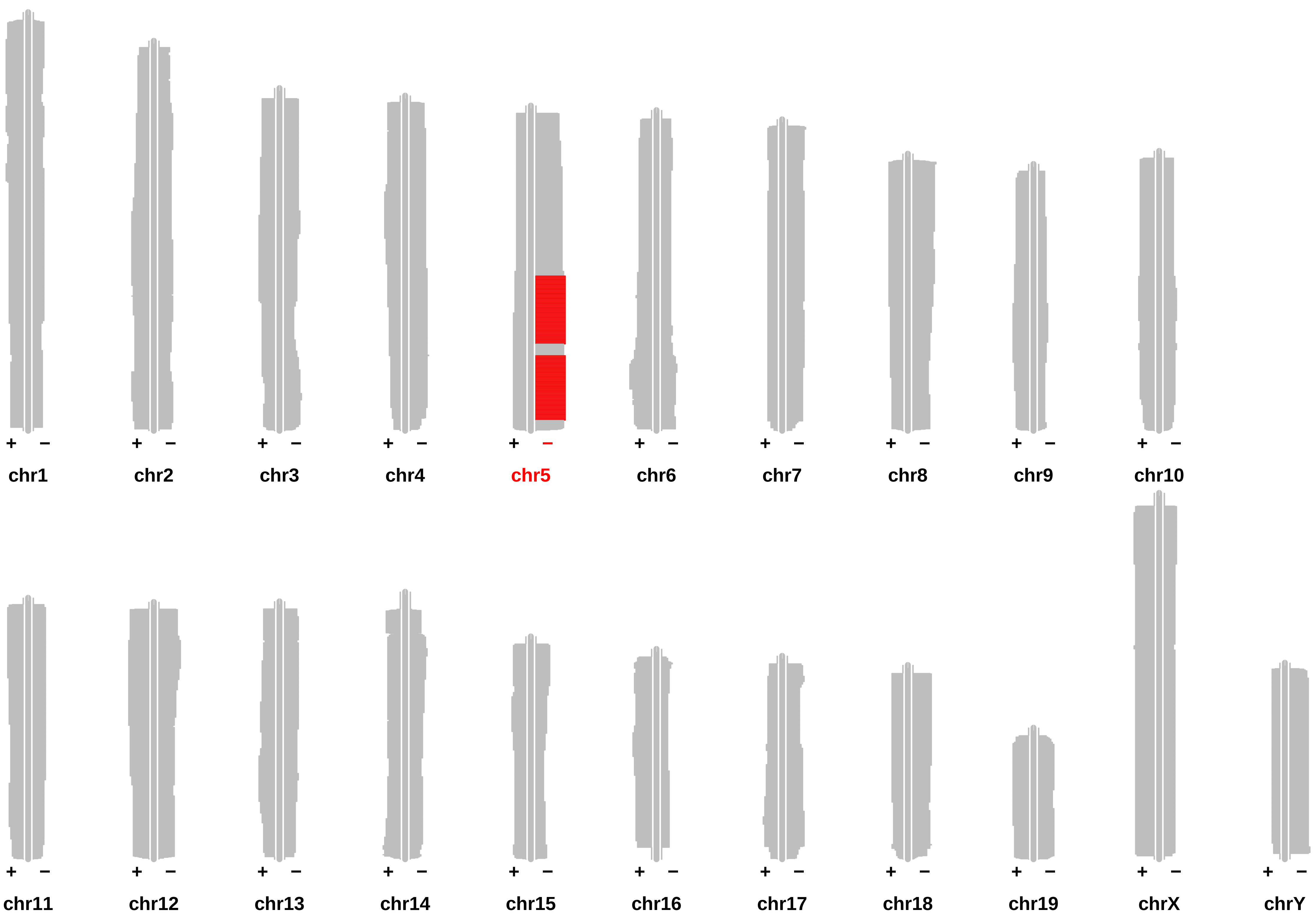

Quality filter=20

Fragment= chr5_JH584296_random Organism= Mus_musculus Peak agreement= 80 %

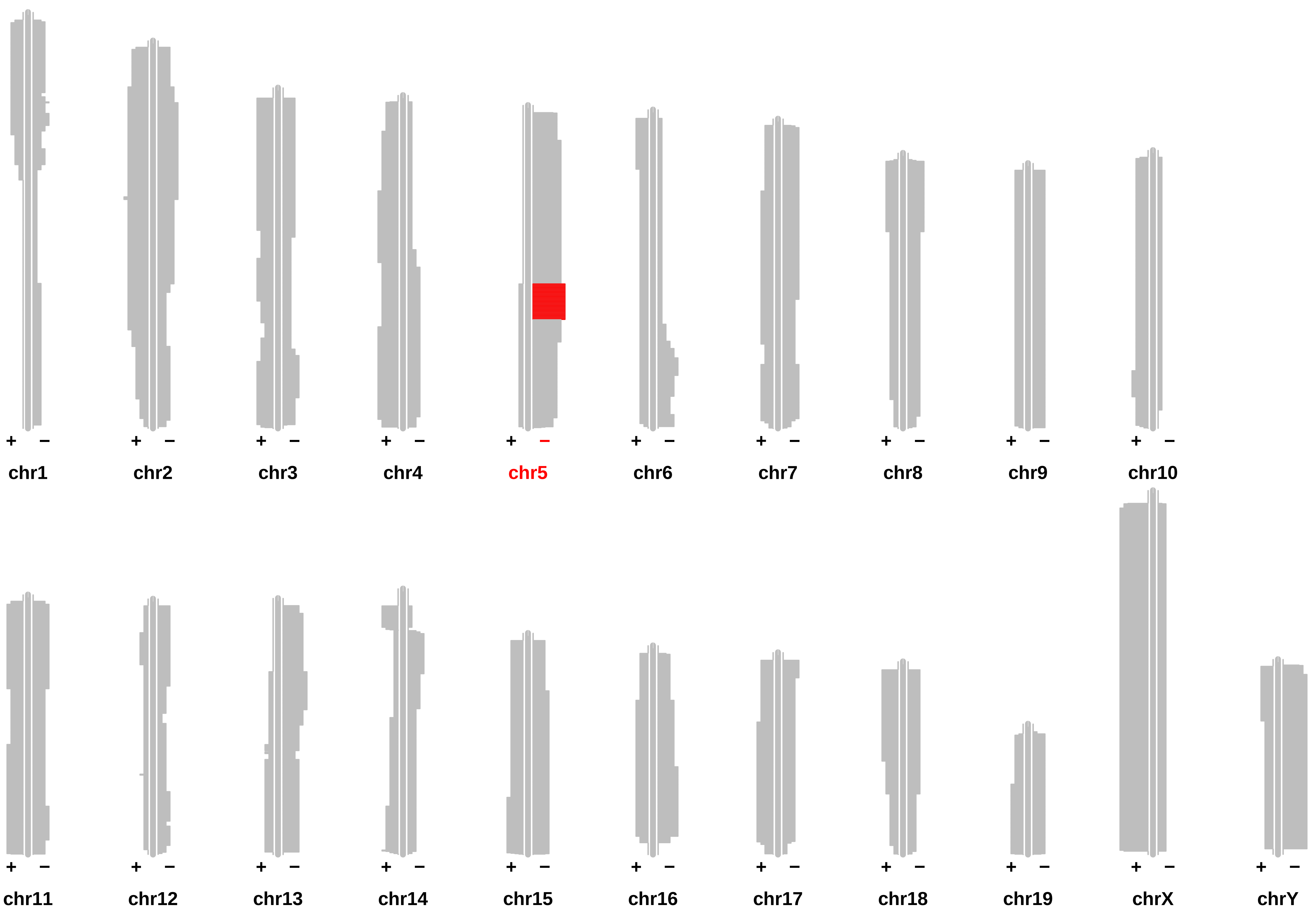

Quality filter=20

Fragment= chr5_JH584297_random Organism= Mus_musculus Peak agreement= 100 %

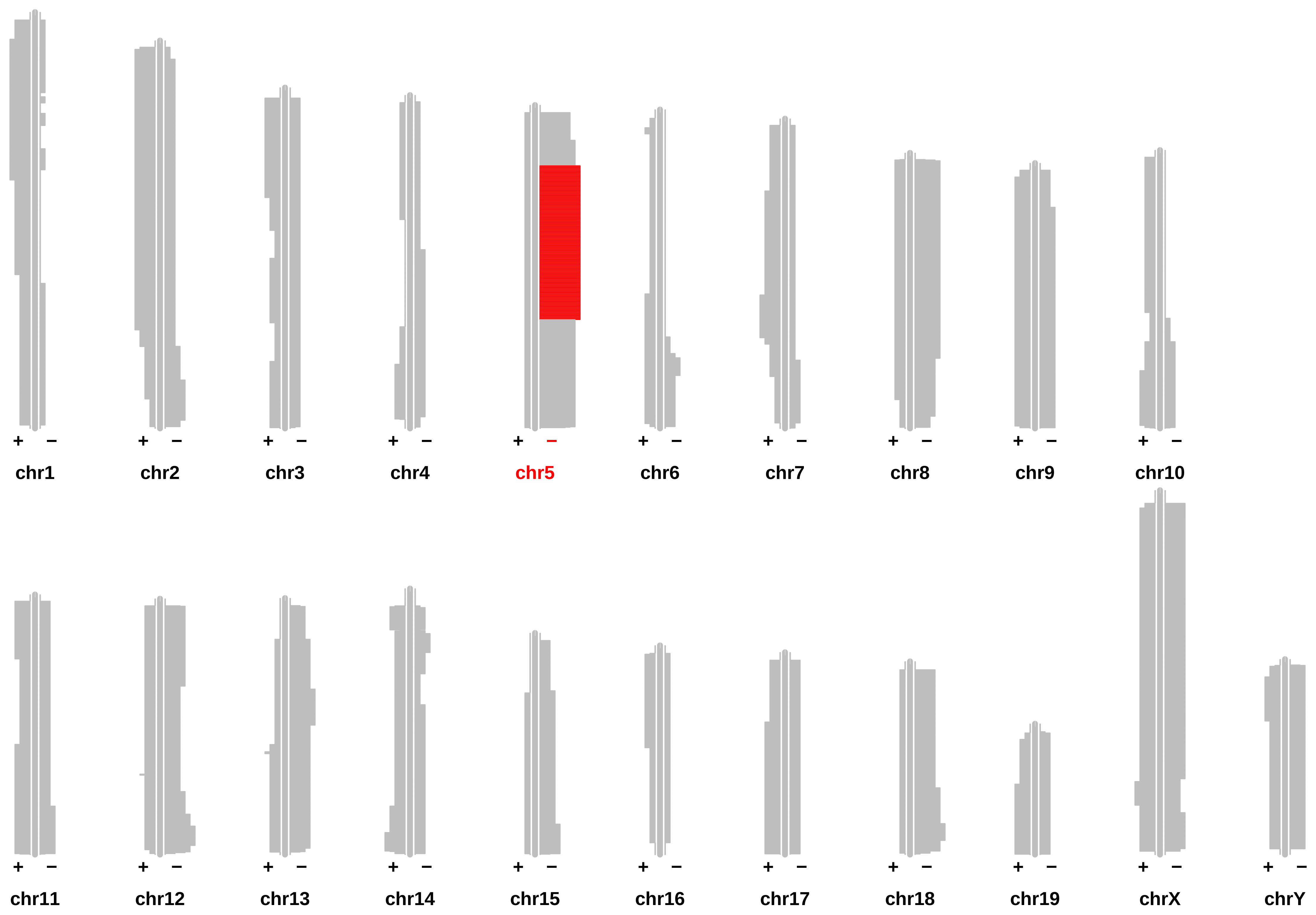

Quality filter=20

Fragment= chr5_JH584299_random Organism= Mus_musculus Peak agreement= 88.24 %

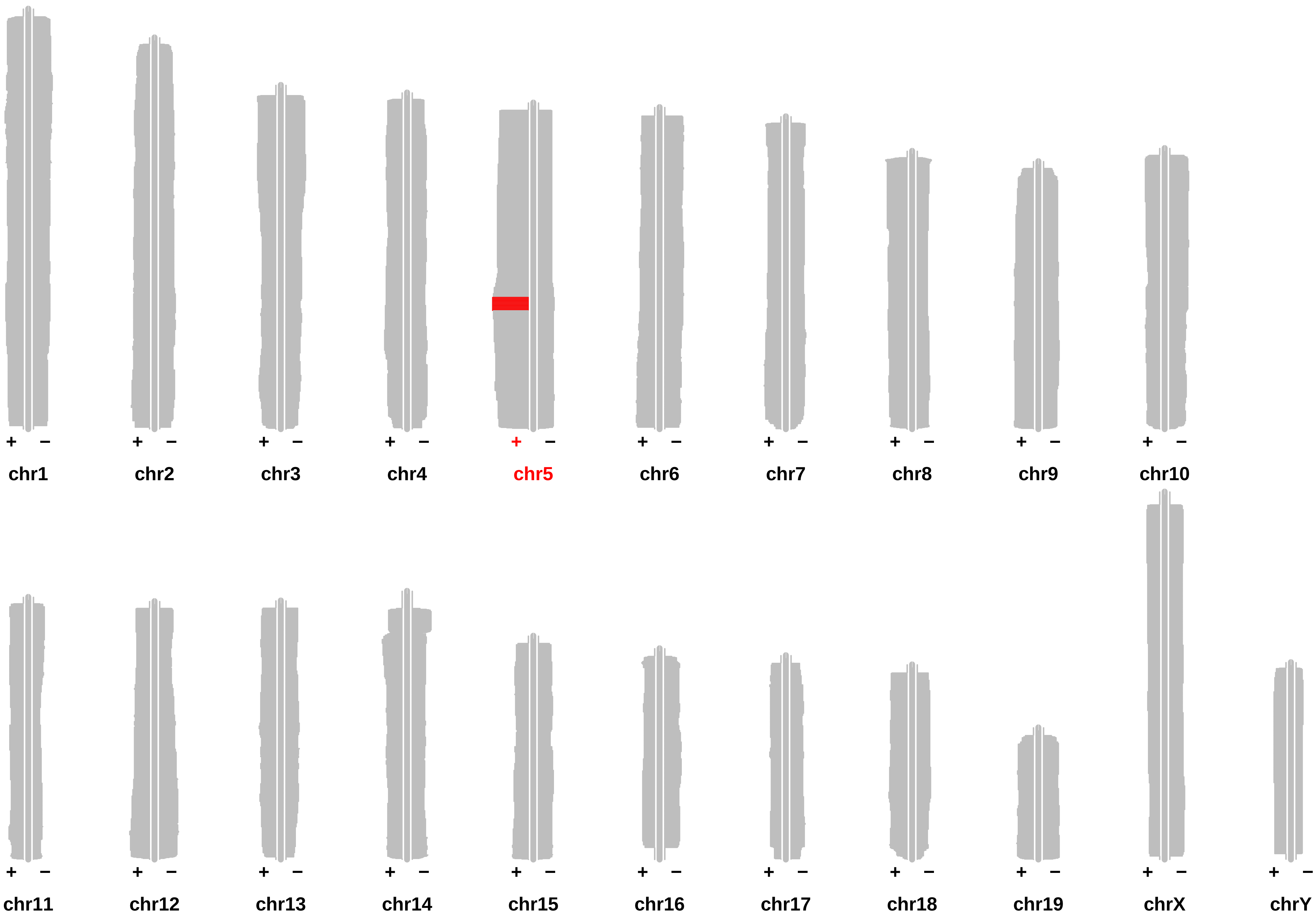

Quality filter=20

Fragment= chr4_GL456216_random Organism= Mus_musculus Peak agreement= 78 %

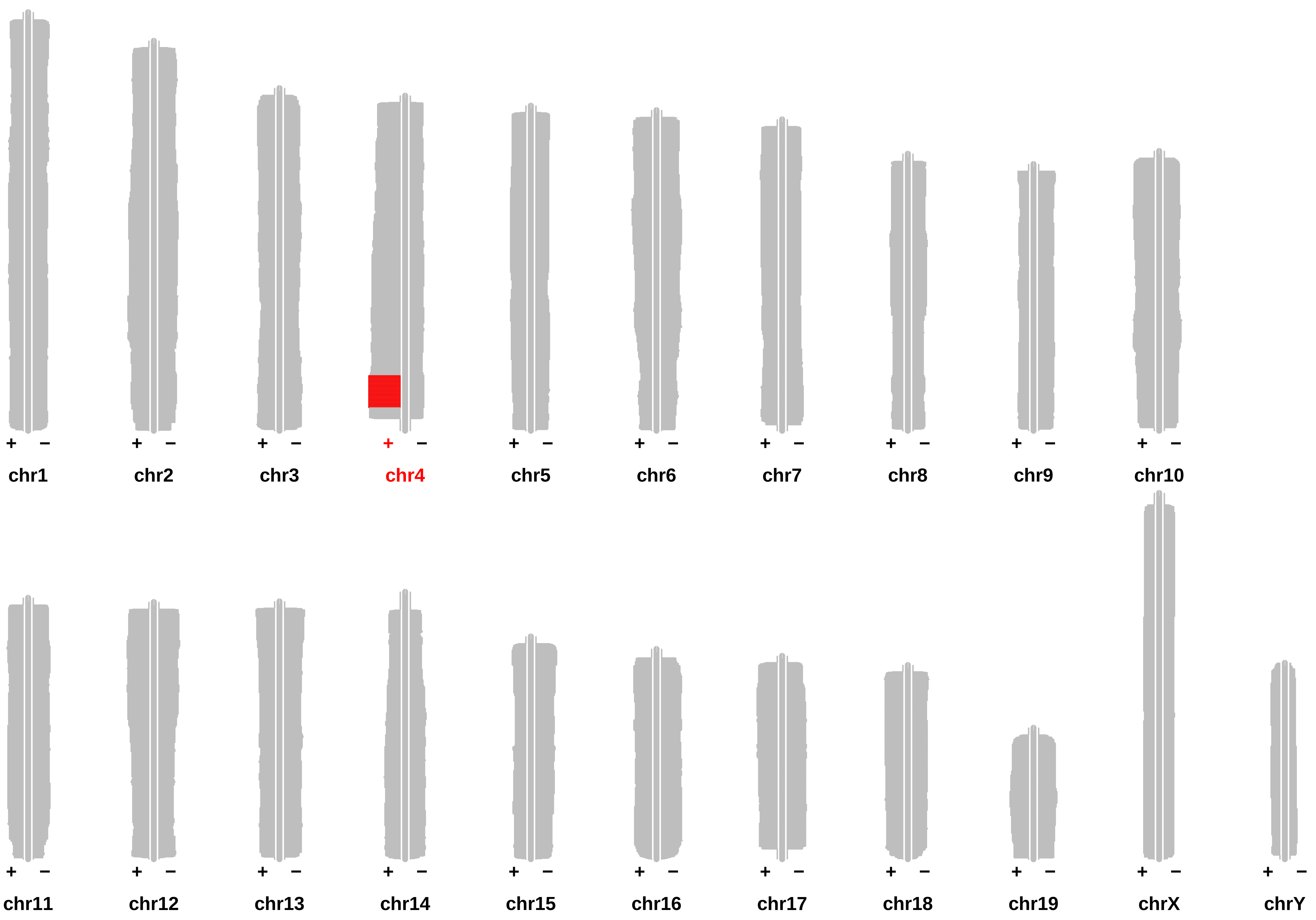

Quality filter=20

Fragment= chrUn_GL456383 Organism= Mus_musculus **Peak agreement= 81.82 %**

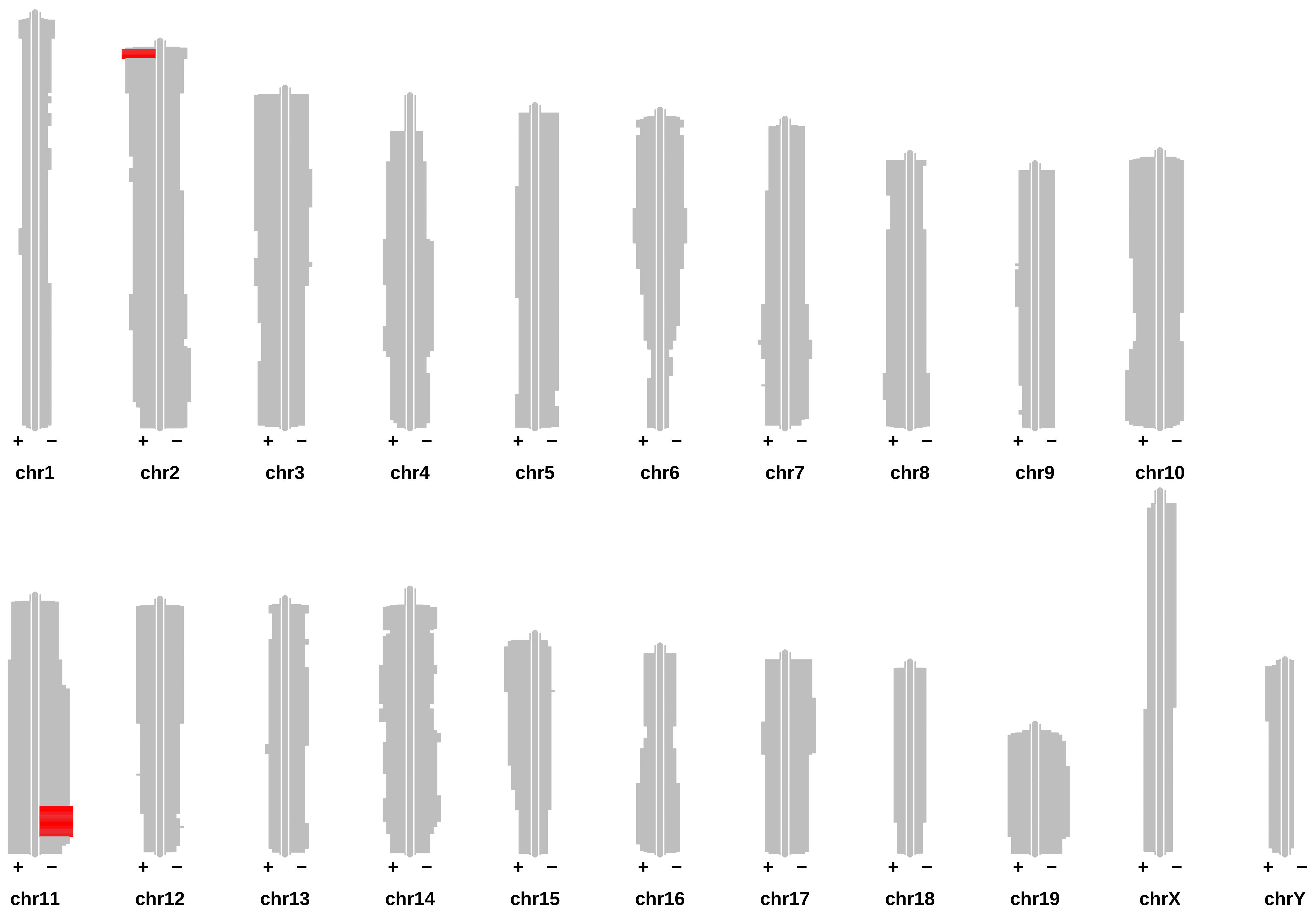

Quality filter=20

Fragment= chrUn_GL456390 Organism= Mus_musculus Peak agreement= 75 %

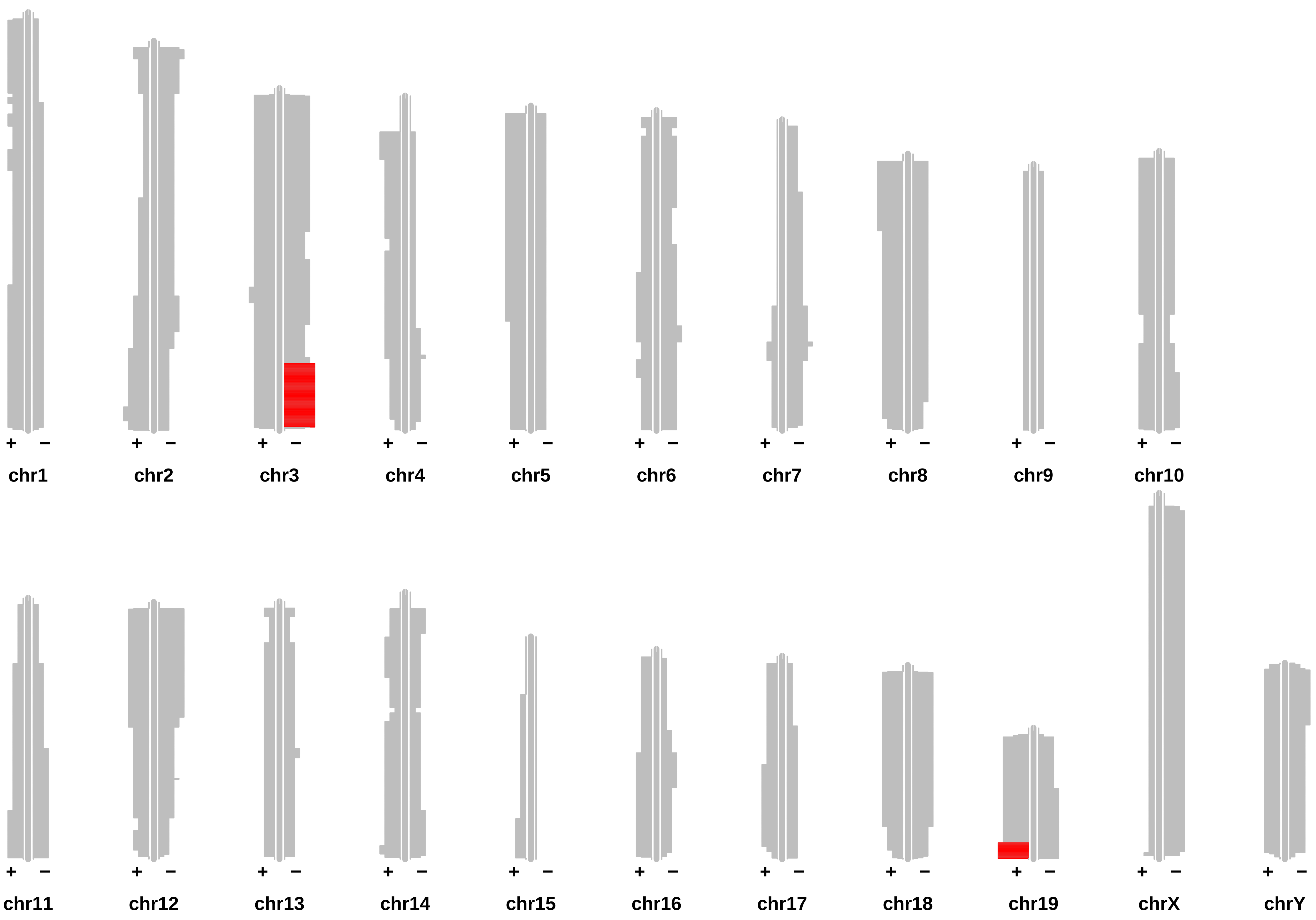

Quality filter=20

Fragment= chr4_JH584295_random Organism= Mus_musculus Peak agreement= 100 %

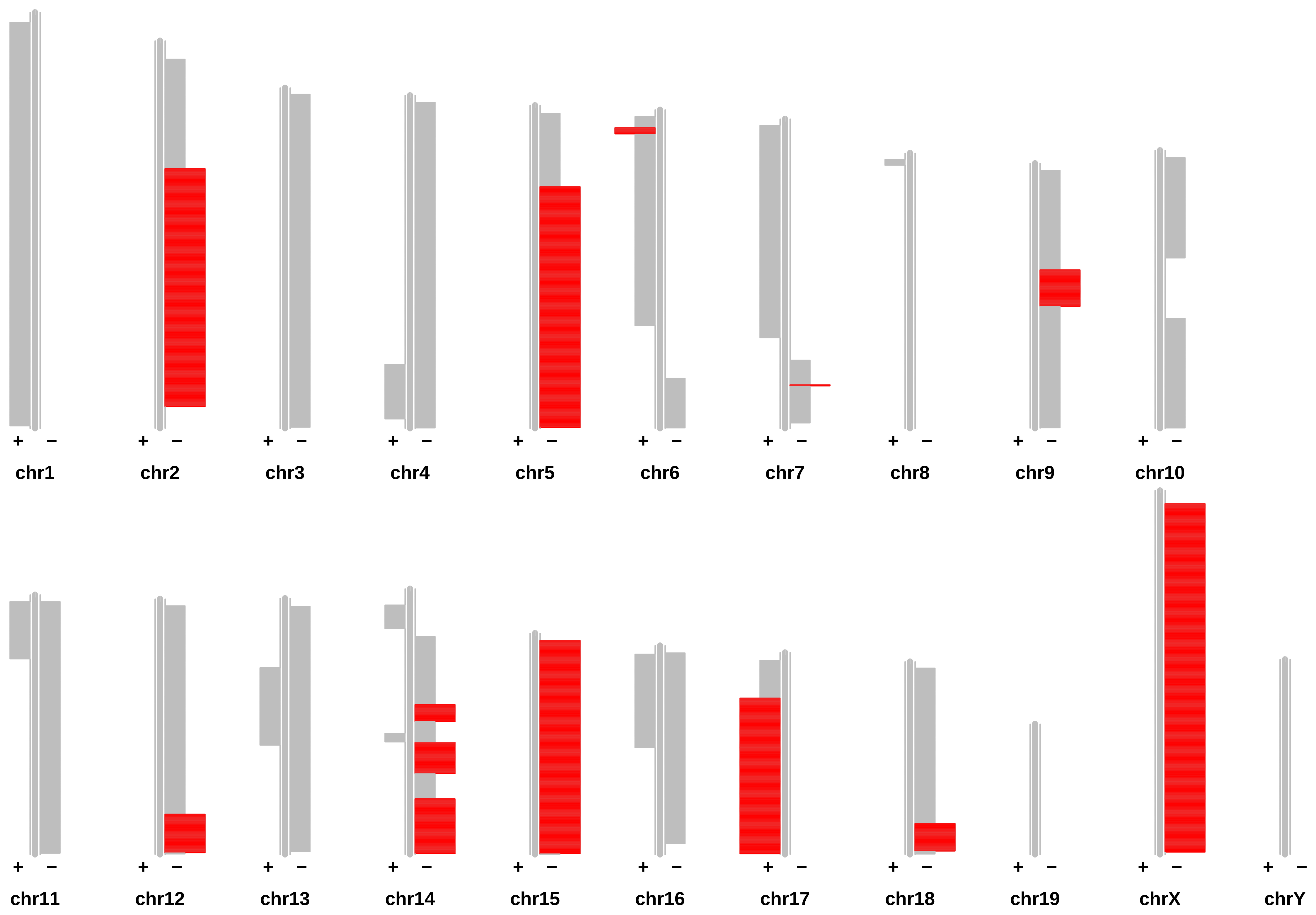

Quality filter=20

Fragment= chr4_GL456350_random Organism= Mus_musculus Peak agreement= 100 %

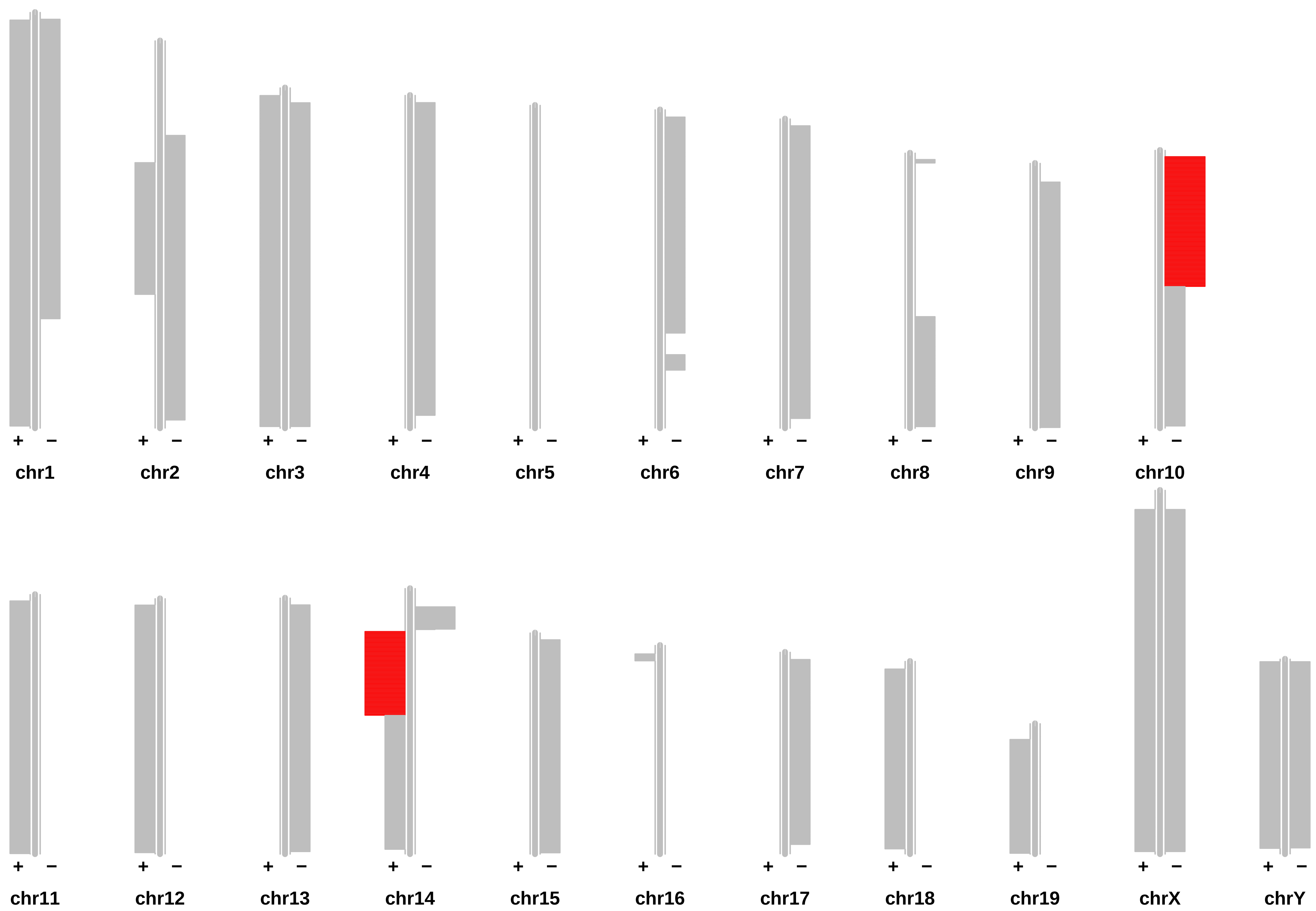

Quality filter=20
